# Predictive Performance of Cardiovascular Risk Scores in Cancer Survivors From the UK Biobank

**DOI:** 10.1016/j.jaccao.2024.05.015

**Published:** 2024-07-23

**Authors:** Celeste McCracken, Dorina-Gabriela Condurache, Liliana Szabo, Hussein Elghazaly, Fiona M. Walter, Adam J. Mead, Ronjon Chakraverty, Nicholas C. Harvey, Charlotte H. Manisty, Steffen E. Petersen, Stefan Neubauer, Zahra Raisi-Estabragh

**Affiliations:** a Division of Cardiovascular Medicine, Radcliffe Department of Medicine, University of Oxford, National Institute for Health Research Oxford Biomedical Research Centre, Oxford University Hospitals NHS Foundation Trust, Oxford, United Kingdom; b Barts Heart Centre, St Bartholomew’s Hospital, Barts Health NHS Trust, West Smithfield, London, United Kingdom; c William Harvey Research Institute, National Institute for Health and Care Research Barts Biomedical Research Centre, Queen Mary University of London, Charterhouse Square, London, United Kingdom; d Heart and Vascular Centre, Semmelweis University, Budapest, Hungary; e Department of Medicine, Imperial College London and Imperial College NHS Trust, South Kensington, London, United Kingdom; f Wolfson Institute of Population Health, Queen Mary University of London, Charterhouse Square, London, United Kingdom; g Department of Public Health and Primary Care, University of Cambridge, Cambridge, United Kingdom; h Medical Research Council Weatherall Institute of Molecular Medicine, National Institute for Health Research Oxford Biomedical Research Centre, Oxford University Hospitals NHS Foundation Trust, Oxford, United Kingdom; i MRC Lifecourse Epidemiology Centre, University of Southampton, Southampton, United Kingdom; j National Institute for Health and Care Research Southampton Biomedical Research Centre, University of Southampton, University Hospital Southampton NHS Foundation Trust, Southampton, United Kingdom; k Institute of Cardiovascular Science, University College London, London, United Kingdom; l Health Data Research UK, London, United Kingdom

**Keywords:** cancer survivors, cardio-oncology, cardiovascular risk score, cohort studies, preventive medicine

## Abstract

**Background:**

Cardiovascular preventive strategies are guided by risk scores with unknown validity in cancer cohorts.

**Objectives:**

This study aimed to evaluate the predictive performance of 7 established cardiovascular risk scores in cancer survivors from the UK Biobank.

**Methods:**

The predictive performance of QRISK3, Systematic Coronary Risk Evaluation 2 (SCORE2)/Systematic Coronary Risk Evaluation for Older Persons (SCORE-OP), Framingham Risk Score, Pooled Cohort equations to Prevent Heart Failure (PCP-HF), CHARGE-AF, QStroke, and CHA_2_DS_2_-VASc was calculated in participants with and without a history of cancer. Participants were propensity matched on age, sex, deprivation, health behaviors, family history, and metabolic conditions. Analyses were stratified into any cancer, breast, lung, prostate, brain/central nervous system, hematologic malignancies, Hodgkin lymphoma, and non-Hodgkin lymphoma. Incident cardiovascular events were tracked through health record linkage over 10 years of follow-up. The area under the receiver operating curve, balanced accuracy, and sensitivity were reported.

**Results:**

The analysis included 31,534 cancer survivors and 126,136 covariate-matched controls. Risk score distributions were near identical in cases and controls. Participants with any cancer had a significantly higher incidence of all cardiovascular outcomes than matched controls. Performance metrics were significantly worse for all risk scores in cancer cases than in matched controls. The most notable differences were among participants with a history of hematologic malignancies who had significantly higher outcome rates and poorer risk score performance than their matched controls. The performance of risk scores for predicting stroke in participants with brain/central nervous system cancer was very poor, with predictive accuracy more than 30% lower than noncancer controls.

**Conclusions:**

Existing cardiovascular risk scores have significantly worse predictive accuracy in cancer survivors compared with noncancer comparators, leading to an underestimation of risk in this cohort.

Cancer survivors are a growing cohort with distinct disease susceptibilities and health care needs who are often overlooked in existing models of care.^1^, ^2^, ^3^ The heightened risk of cardiovascular disease (CVD) in cancer patients is widely recognized and attributed to shared risk factors, the toxicity of cancer therapies, and biologic pathways related to the cancer itself.^4^ Recent reports highlight a persistently elevated CVD risk extending beyond the duration of cancer treatment into long-term survivorship.^5^^,^^6^

Cardio-oncology has emerged as a subspecialty dedicated to the cardiovascular care of cancer patients.^4^ However, a stronger evidence base is needed to guide clinical care. In clinical practice, CVD prevention strategies are guided by risk prediction tools such as the QRISK3^7^ and the Framingham Risk Score (FRS).^8^ These instruments, developed and validated in the general population, do not include cancer-specific predictors. As such, they may underestimate the risk of CVD in cancer survivors, leading to undertreatment of this vulnerable population.

This study compared the predictive performance of 7 established cardiovascular risk scores in cancer survivors compared with matched noncancer controls from the UK Biobank, considering a range of cardiovascular outcomes and differential relationships by cancer type.

## Methods

### Ethical approval

This study complies with the Declaration of Helsinki. Ethical approval for UK Biobank studies was granted by the NHS National Research Ethics Service on June 17, 2011 (reference 11/NW/0382) and extended on June 18, 2021 (reference 21/NW/0157). Written informed consent was obtained from all participants.

### Setting and study population

The UK Biobank is a prospective cohort of over 500,000 participants. UK residents aged 40 to 69 years and living within 25 miles of 1 of 22 assessment centers in urban and rural areas were identified through NHS registers and recruited between 2006 and 2010. Baseline assessment included medical, social, demographic, lifestyle, environmental, and physical parameters.^9^ Individuals who were unable to consent or complete baseline assessment because of illness or discomfort were not recruited. Extensive health record linkage has been established for the entire cohort, allowing prospective tracking of incident health events.

### Ascertainment of cancer status

Cancer status at baseline recruitment was ascertained based on International Classification of Disease codes in linked Hospital Episode Statistics and National Cancer Registration and Analysis Service records (Supplemental Table 1). The date of the first occurrence and the main cancer site were identified by the first cancer record in any linked database. We began with a set of 25 cancer types, following the work of Strongman et al,^5^ and identified subsets with sufficient outcome counts for analysis. The final cancer groups were as follows: any cancer, breast, lung, prostate, brain/central nervous system (CNS), hematologic, Hodgkin lymphoma, and non-Hodgkin lymphoma. Hematologic cancer included all lymphomas, leukemia, multiple myeloma, polycythemia vera, myelodysplastic syndrome, and rarer blood cancers. Individuals without any record of cancer were considered as potential noncancer comparators.

### Cardiovascular risk scores

We considered 7 risk scores currently used in standard clinical practice (Supplemental Table 2). The QRISK3,^7^ FRS,^8^ and SCORE2/SCORE-OP^10^^,^^11^ scores all have a composite endpoint of myocardial infarction, stroke, or cardiovascular mortality, which we labeled CVD 1. We created a second enriched composite cardiovascular endpoint by adding incident heart failure, atrial fibrillation (AF), nonischemic cardiomyopathies, and valvular heart disease to CVD 1, which we labeled CVD 2. The CVD 2 outcome does not match the original intended outcome of the tested risk scores but is presented to provide a broader category of CVDs to which cancer survivors may be more susceptible (eg, nonischemic CVDs).

We implemented CHARGE-AF^12^ for the risk of AF, PCP-HF^13^ to assess the risk of heart failure, and 2 scores (QStroke^14^ and CHA_2_DS_2_-VASc^15^) for the risk of stroke. Although the CHA_2_DS_2_-VASc^15^ score was originally developed for estimating the risk of stroke in AF cohorts, its predictive utility has also been demonstrated in patients without AF.^16^ Cardiovascular risk scores were calculated for each participant based on data available at the baseline visit using equations reported in published sources as detailed in Supplemental Table 2.

### Ascertainment of covariates

Age, self-reported sex, systolic blood pressure, and anthropomorphic measurements were recorded at baseline along with self-reported information about ethnicity, education, family history, smoking, alcohol use, and physical activity. We calculated the average and SD of 2 blood pressure readings recorded at baseline. The Townsend deprivation index at baseline was assigned based on participant postcode. Ethnic groups and smoking categories were coded to match QRISK3/QStroke specifications. Physical activity was coded as binary; “active” was ≥600 summed metabolic equivalent task minutes per week based on aggregation of physical activity fields according to published guidance. Family history was aggregated from self-reported illnesses of mother, father, and siblings when age of diagnosis was not recorded. Serum total cholesterol, high-density lipoprotein cholesterol, and glycated hemoglobin levels were measured at the baseline assessment. Missing values in risk score variables were imputed using either replacement with the mean (Townsend deprivation index), replacement with the majority category (ethnicity and smoking), or multiple imputation with chained equations (Supplemental Table 3).

### Ascertainment of outcomes

Cardiovascular outcome definitions were matched to those of each risk score, referring to the original development of each risk score. The following incident outcomes were considered: AF, stroke, heart failure, CVD 1, and CVD 2. The record of diagnosis and the date of the first occurrence were extracted by searching across all available linked records. Incident events were those occurring after recruitment. Individuals with record of an outcome at baseline were excluded from the study sample. The latest censor date was October 2022, giving a median follow-up time of 13.6 years (Q1-Q3: 12.8-14.3 years) across the whole sample. The main analyses assessed outcomes at 10 years to be congruent with the 10-year risk predicted by candidate risk scores. Ninety-four percent of the study sample had at least 10 years of follow-up.

### Case-control matching

Each cancer-exposed participant was matched to 4 noncancer controls using propensity score matching with a caliper width of 0.2, as per published recommendations,^17^ using the following predefined covariates: age, sex, ethnicity, body mass index (BMI), deprivation, family history of heart disease or stroke, systolic blood pressure, hypertension, diabetes, high cholesterol, total cholesterol, high-density lipoprotein cholesterol, glycated hemoglobin, current smoking, physical activity, and alcohol intake frequency. A summary of the matched and unmatched standardized mean differences is provided in Supplemental Figure 1.

### Statistical analysis

Analysis was performed using R version 4.1.2 (The R Foundation for Statistical Computing, Posit Software, PBC) and RStudio 2022.07.1. Descriptive statistics are presented as counts and percentages for categoric variables and the mean ± SD or median with 25th and 75th percentiles (Q1-Q3). Cardiovascular risk score values were calculated for all participants as per published equations. The 10-year cumulative outcome incidence and 95% CIs were calculated using a Fine-Gray model to account for the competing risk of death for cancer survivors and matched controls. Calibration statistics were calculated by assessing 10-year cumulative incidence across each decile of the risk score distribution, again accounting for competing risk.

The discriminative performance of each risk score in predicting its intended outcome was assessed in cancer and control groups separately using time-dependent area under the receiver operating curve (AUC) in the presence of competing risks.^18^^,^^19^ The AUC reflects the chance that the risk score correctly assigns a higher value to the person with the disease compared with the one without it.^20^ In addition to AUC, sensitivity (the proportion of true outcomes correctly identified) and balanced accuracy (the average of sensitivity and specificity) were assessed as static performance metrics on the 10-year outcomes.

Although AUC is agnostic to the score cutoff threshold, sensitivity and balanced accuracy are not. Therefore, for each cancer index–outcome combination, the cutoff threshold was set at the point where balanced accuracy was highest in the noncancer control group (80% of the data) and was then applied to the cancer group. In other words, both cancer and matched controls were scored for accuracy with the same threshold value. Standard errors and CIs for static performance metrics were derived from bootstrapping and permutation testing with 1,000 replicates.

### Data sharing statement

This project was performed under UK Biobank access application 59867. UK Biobank will make the data available to all bona fide researchers for all types of health-related research that is in the public interest without preferential or exclusive access for any persons. All researchers will be subject to the same application process and approval criteria as specified by UK Biobank. For more details on the access procedure, see the UK Biobank website.

## Results

### Baseline characteristics

From the initial set of 501,072 participants, 3,926 (0.8%) were excluded because of missing height/weight, sex incongruity (eg, prostate cancer in women), or cancer ambiguity (eg, a chemotherapy record with no cancer registered). Participants with a record of carcinoma in situ (4,197 [0.8%]) or nonmelanoma skin lesion (1,327 [0.3%]) who had not progressed to another cancer diagnosis by baseline were excluded. Participants with pre-existing heart disease (n = 44,117 [8.8%]) or stroke (n = 6,075 [1.2%]) at baseline were excluded. From the remaining 441,433 participants, 31,534 (7.1%) had a record of cancer at baseline. Among those without any record of cancer (n = 409,899), 126,136 (30.8%) were propensity matched to the cancer participants with a ratio of 4 to 1 (Supplemental Figure 2).

Table 1 provides baseline characteristics of participants with any cancer (n = 31,534) and their 126,136 matched controls and the following specific cancer subgroups: breast (n = 10,167), lung (n = 246), prostate (n = 2,845), brain/CNS (n = 254), hematologic (n = 2,538), non-Hodgkin lymphoma (n = 884), and Hodgkin lymphoma (n = 374). Full comparative summaries are available in Supplemental Tables 4 and 5 along with rarer hematologic conditions for further reference.

**Table 1:** Cancer Cases and Matched Controls Characteristics

	Cancer Cases (n = 31,534)	Matched Controls (4:1) (n = 126,136)	Breast (n = 10,167)	Lung (n = 246)	Prostate (n = 2,845)	Brain/CNS (n = 254)	Hematologic (n = 2,538)	Non-Hodgkin’s Lymphoma (n = 884)	Hodgkin’s Lymphoma (n = 374)
Average age, y	59.4 ± 7.2	59.4 ± 7.2	59.6 ± 6.7	60.6 ± 6.5	63.7 ± 4.4	54.9 ± 8.5	58.2 ± 7.9	58.4 ± 7.7	53.9 ± 8.1
Age <50 y	3,760 (11.9)	15,040 (11.9)	956 (9.4)	19 (7.7)	22 (0.8)	80 (31.5)	442 (17.4)	145 (16.4)	126 (33.7)
Age 50-59 y	9,332 (29.6)	37,328 (29.6)	3,244 (31.9)	74 (30.1)	422 (14.8)	83 (32.7)	740 (29.2)	263 (29.8)	138 (36.9)
Age 60-69 y	9,468 (30.0)	37,872 (30.0)	3,276 (32.2)	70 (28.5)	943 (33.1)	52 (20.5)	702 (27.7)	246 (27.8)	64 (17.1)
Age 65 y or older	8,974 (28.5)	35,896 (28.5)	2,691 (26.5)	83 (33.7)	1,458 (51.2)	39 (15.4)	654 (25.8)	230 (26.0)	46 (12.3)
Women	20,963 (66.5)	83,852 (66.5)	10,167 (100.0)	113 (45.9)		143 (56.3)	1,154 (45.5)	410 (46.4)	192 (51.3)
Men	10,571 (33.5)	42,284 (33.5)		133 (54.1%)	2,845 (100.0%)	111 (43.7%)	1,384 (54.5%)	474 (53.6%)	182 (48.7%)
Ethnicity other than White	1,055 (3.3)	4,330 (3.4)	340 (3.3)	13 (5.3)	111 (3.9)	17 (6.7)	105 (4.1)	32 (3.6)	16 (4.3)
Above UK median deprivation	9,554 (30.3)	38,045 (30.2)	2,971 (29.2)	116 (47.2)	763 (26.8)	85 (33.5)	806 (31.8)	275 (31.1)	117 (31.3)
Townsend deprivation index	−2.2 (−3.7 to 0.4)	−2.2 (−3.7 to 0.4)	−2.3 (−3.7 to 0.2)	−0.7 (−3.1 to 2.6)	−2.5 (−3.8 to −0.1)	−1.9 (−3.6 to 0.9)	−2.1 (−3.7 to 0.6)	−2.0 (−3.7 to 0.7)	−2.0 (−3.7 to 0.5)
Postsecondary education	17,735 (56.2)	70,966 (56.3)	5,620 (55.3)	117 (47.6)	1,726 (60.7)	163 (64.2)	1,468 (57.8)	551 (62.3)	232 (62.0)
Family history of heart disease	14,012 (44.4)	55,898 (44.3)	4,763 (46.8)	100 (40.7)	1,164 (40.9)	93 (36.6)	1,075 (42.4)	385 (43.6)	137 (36.6)
Family history of stroke	8,872 (28.1)	35,596 (28.2)	3,050 (30.0)	56 (22.8)	765 (26.9)	57 (22.4)	665 (26.2)	231 (26.1)	76 (20.3)
Systolic blood pressure, mm Hg	139.6 ± 9.1	139.5 ± 19.1	138.3 ± 19.3	138.5 ± 20.8	145.5 ± 17.5	136.9 ± 18.6	138.3 ± 19.0	137.7 ± 18.5	135.9 ± 20.5
Total cholesterol, mmol/L	5.8 ± 1.2	5.8 ± 1.1	6.0 ± 1.1	5.7 ± 1.2	5.5 ± 1.1	5.9 ± 1.2	5.6 ± 1.2	5.7 ± 1.2	5.8 ± 1.1
HDL cholesterol, mmol/L	1.5 ± 0.4	1.5 ± 0.4	1.6 ± 0.4	1.4 ± 0.4	1.3 ± 0.3	1.4 ± 0.4	1.4 ± 0.4	1.4 ± 0.4	1.5 ± 0.4
HbA1c, mmol/mol	35.6 (33.2-38.2)	35.5 (33.2-38.1)	35.9 (33.5-38.4)	36.5 (34.1-39.6)	35.7 (33.3-38.3)	35.2 (32.0-37.1)	35.3 (32.5-38.1)	35.0 (32.5-37.7)	35.2 (32.6-38.0)
Current smoker	3,100 (9.8)	12,413 (9.8)	783 (7.7)	37 (15.0)	233 (8.2)	26 (10.2)	264 (10.4)	79 (8.9)	45 (12.0)
Body mass index, kg/m^2^	26.6 (24.0-29.9)	26.6 (24.0-29.9)	26.2 (23.6-29.5)	26.6 (24.0-29.9)	27.3 (25.0-29.8)	27.4 (24.2-30.9)	26.6 (24.1-29.9)	26.6 (23.9-29.4)	25.8 (23.5-29.3)
Obesity	7,713 (24.5)	30,620 (24.3)	2,267 (22.3)	59 (24.0)	672 (23.6)	80 (31.5)	621 (24.5)	201 (22.7)	79 (21.1)
Alcohol intake daily, yes	6,416 (20.3)	25,661 (20.3)	1,778 (17.5)	47 (19.1)	799 (28.1)	35 (13.8)	492 (19.4)	184 (20.8)	65 (17.4)
Physically active, yes^a^	22,722 (72.1)	91,011 (72.2)	7,287 (71.7)	162 (65.9)	2,181 (76.7)	183 (72.0)	1,777 (70.0)	651 (73.6)	261 (69.8)
Hypertension	10,120 (32.1)	40,348 (32.0)	2,837 (27.9)	72 (29.3)	1,178 (41.4)	70 (27.6)	833 (32.8)	261 (29.5)	84 (22.5)
High cholesterol	5,599 (17.8)	22,292 (17.7)	1,451 (14.3)	52 (21.1)	795 (27.9)	42 (16.5)	427 (16.8)	147 (16.6)	52 (13.9)
Diabetes	1,718 (5.4)	6,686 (5.3)	421 (4.1)	15 (6.1)	192 (6.7)	8 (3.1)	154 (6.1)	45 (5.1)	15 (4.0)
10-y outcomes									
CVD 1	1,542 (4.9)	5,423 (4.3)	302 (3.0)	20 (8.1)	218 (7.7)	25 (9.8)	227 (8.9)	55 (6.2)	45 (12.0)
CVD 2	3,636 (11.5)	12,087 (9.6)	847 (8.3)	52 (21.1)	464 (16.3)	35 (13.8)	533 (21.0)	145 (16.4)	89 (23.8)
Atrial fibrillation	1,707 (5.4)	5,693 (4.5)	397 (3.9)	29 (11.8)	234 (8.2)	13 (5.1)	230 (9.1)	65 (7.4)	29 (7.8)
Heart failure	789 (2.5)	2,204 (1.7)	188 (1.8)	17 (6.9)	97 (3.4)	2 (0.8)	165 (6.5)	50 (5.7)	25 (6.7)
Stroke	664 (2.1)	2,072 (1.6)	151 (1.5)	7 (2.8)	84 (3.0)	19 (7.5)	86 (3.4)	20 (2.3)	12 (3.2)
Full follow-up time, y	13.5 (12.6-14.3)	13.6 (12.9-14.4)	13.5 (12.6-14.3)	12.4 (5.7-13.9)	13.4 (12.5-14.2)	13.1 (12.4-14.0)	13.2 (12.3-14.1)	13.3 (12.4-14.1)	13.4 (12.5-14.3)

Values are mean ± SD, n (%), or median (Q1-Q3).

CNS = central nervous system; CVD = cardiovascular disease; HbA1c = glycated hemoglobin; HDL = high-density lipoprotein.

a Physically active is defined as >600 summed metabolic equivalent task minutes per week. Median UK Townsend deprivation index = −0.35 as per 2011 UK Census. CVD 1 = combined endpoint including nonfatal myocardial infarction, nonfatal stroke, or cardiovascular mortality where cardiovascular mortality is defined as any death with a primary cause from International Classification of Diseases-Tenth Revision codes I00-I80. CVD 2 = combined endpoint including everything from CVD 1 plus incident atrial fibrillation, heart failure, nonischemic cardiomyopathies and valvular heart disease.

In both the cancer and control groups, the average age was 59.4 ± 7.2 years, with 66.5% being women and 33.5% men. Thirty percent were above the UK median Townsend deprivation score, and 9.8% were current smokers. Among the cancer cohort, the prevalence of hypertension, diabetes, and high cholesterol was 32.1%, 5.4%, and 17.8%, respectively. Participants with past prostate cancer were the oldest, with an average age of 63.7 ± 4.4 years, whereas those with brain/CNS cancers were the youngest, with an average age of 54.9 ± 8.5 years.

### Predicted risk and observed outcomes

For all cancer index–outcome combinations, the matching procedure produced case and control groups that had near identical risk score profiles, with no significant differences in risk score estimates (Supplemental Figure 3, Supplemental Table 6). Over 10 years of follow-up, cardiovascular events (CVD 2) were observed in 11.5% of participants with a record of any cancer compared with 9.6% of noncancer comparators (Table 1, Supplemental Table 6).

#### Any cancer

**Figure 1:**
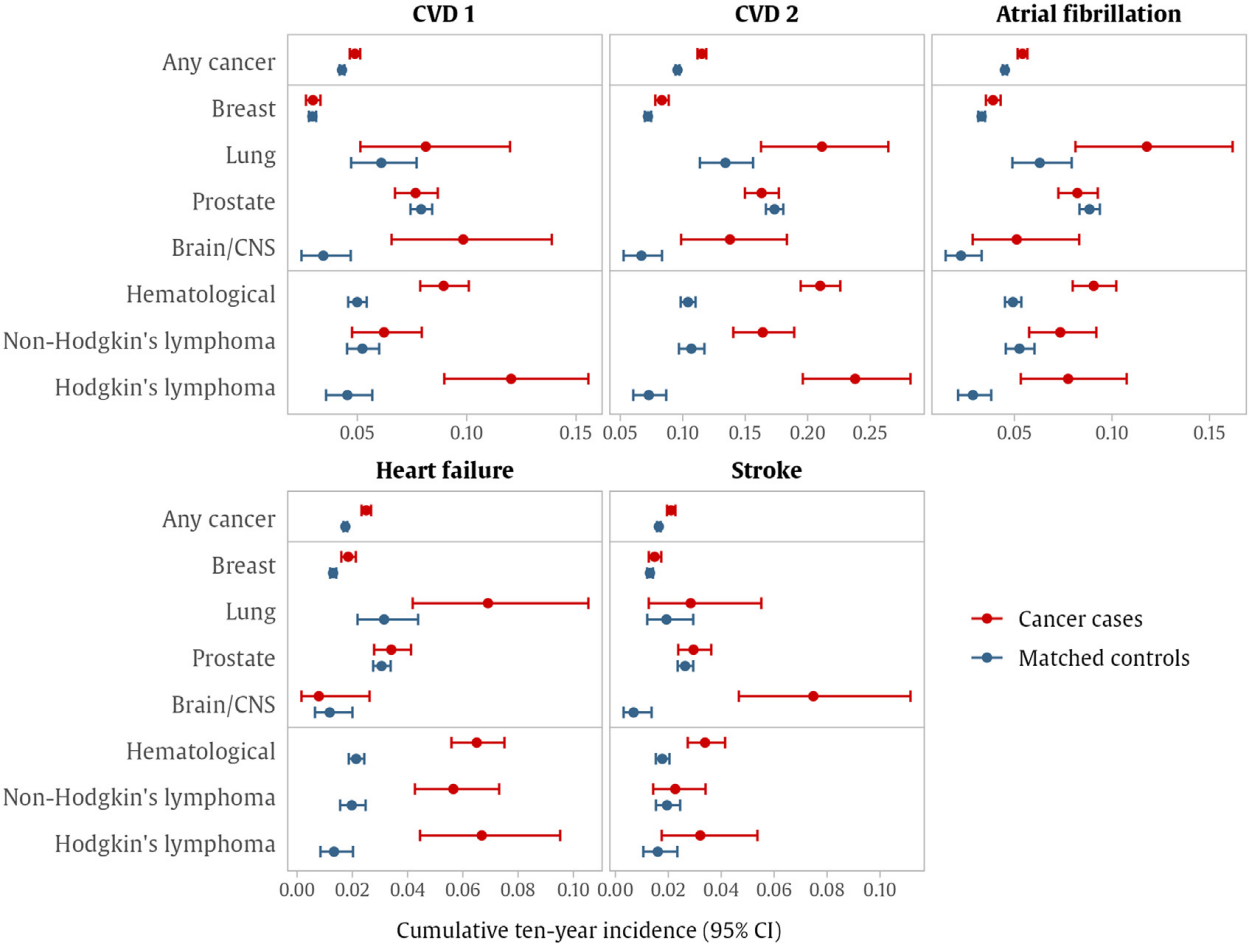
Observed 10-Year Outcome Rates Points represent the cumulative 10-year incidence observed in cancer groups (red) and their matched control cohorts (blue), derived using Fine-Gray models accounting for the competing risk of death, with 95% exact CIs. Cardiovascular disease (CVD) endpoint (CVD 1) includes nonfatal myocardial infarction, nonfatal stroke, or cardiovascular mortality, defined as any death with a primary cause from International Classification of Diseases-10th Revision codes I00-I80. CVD 2 includes everything from CVD 1 plus incident atrial fibrillation, heart failure, nonischemic cardiomyopathies, and valvular heart disease. CNS = central nervous system.

Within any cancer group, the 10-year cumulative incidence was higher in cancer cases than in matched controls for all outcomes considered (Supplemental Table 7, Figure 1, Supplemental Figure 4). In terms of overall calibration and performance, SCORE2/OP had the best calibration and sensitivity for predicting CVD 1 in both cancer cases and controls, whereas QRISK3 had the best performance for CVD 2 in terms of calibration and AUC in both groups. When reporting for the cancer subgroups, only differences from this pattern will be mentioned.

**Figure 2:**
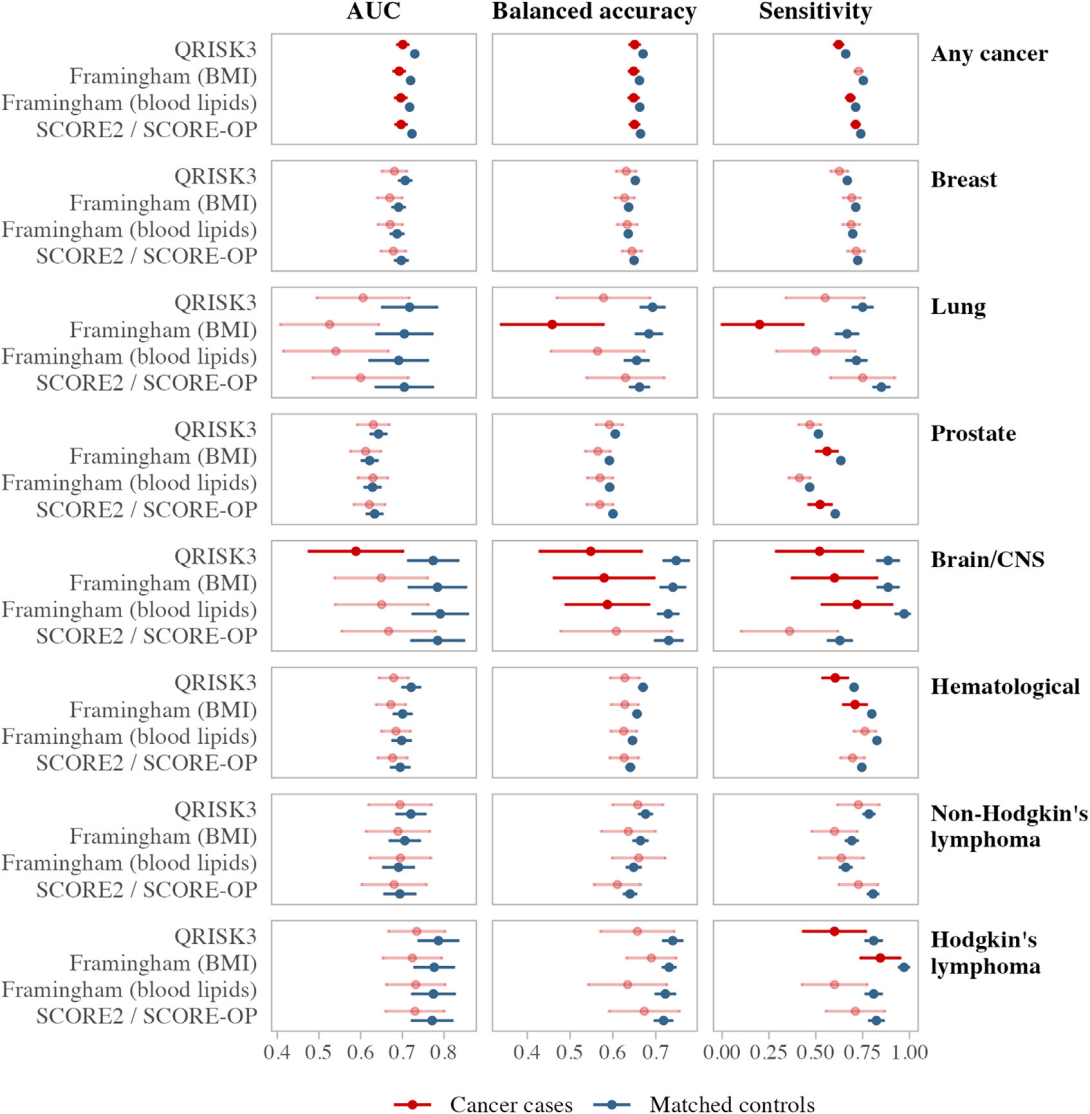
Standard CVD Risk Score Predictive Performance in Cancer Groups vs Matched Controls for CVD 1 The area under the receiver operating curve (AUC), balanced accuracy, and sensitivity for the standard cardiovascular disease endpoint (CVD 1) are compared between cancer groups and their matched control cohorts. Different cancer groups are listed on the right-hand side, whereas candidate CVD risk scores are shown on the left-hand side. Predictive performance results for cancer survivors are displayed in red, with matched controls in dark blue. Cancer groups not significantly different from their noncancer control group are shown in the lighter color. AUC results are from time-dependent analyses accounting for the competing risk of death at the 10-year follow-up. Balanced accuracy and sensitivity are from static performance scoring of 10-year outcomes, with 95% CIs derived from bootstrapping and permutation testing with 1,000 replicates. CVD 1 includes nonfatal myocardial infarction, nonfatal stroke, or cardiovascular mortality, where cardiovascular mortality is defined as any death with a primary cause from International Classification of Diseases-10th Revision codes I00-I80. Abbreviations as in Figure 1.

**Figure 3:**
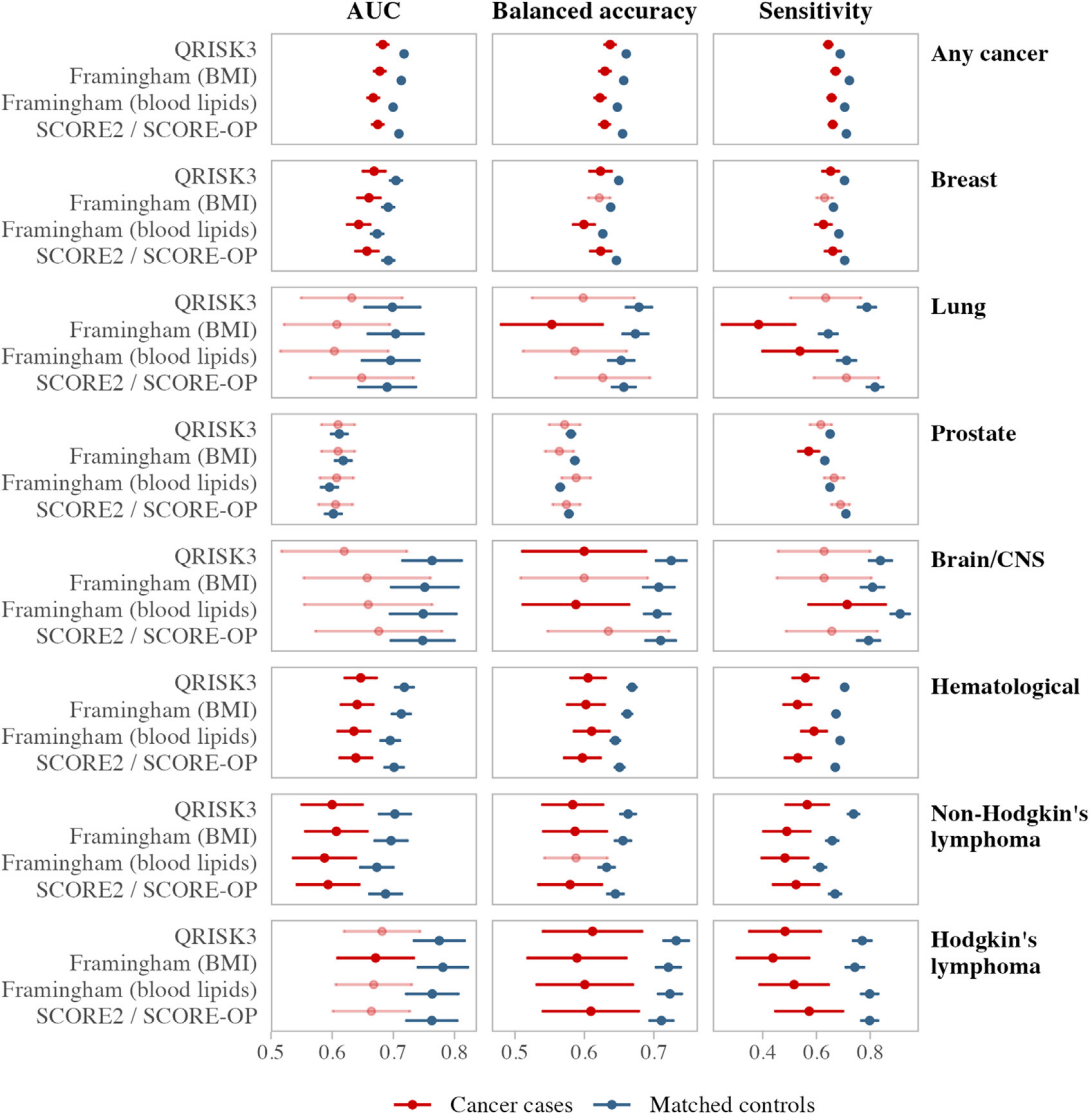
Extended CVD Risk Score Predictive Performance in Cancer Groups vs Matched Controls for CVD 2 The AUC, balanced accuracy, and sensitivity for the broader CVD endpoint (CVD 2) are compared between cancer groups and their matched control cohorts. Different cancer groups are listed on the right-hand side, whereas candidate CVD risk scores are shown on the left-hand side. Predictive performance results for cancer survivors are displayed in red, with matched controls in dark blue. Cancer groups not significantly different from their noncancer control group are shown in the lighter color. AUC results are from time-dependent analyses accounting for the competing risk of death at the 10-year follow-up. Balanced accuracy and sensitivity are from static performance scoring of 10-year outcomes, with 95% CIs derived from bootstrapping and permutation testing with 1,000 replicates. CVD 2 includes nonfatal myocardial infarction, nonfatal stroke, cardiovascular mortality, atrial fibrillation, heart failure, nonischemic cardiomyopathies, and valvular heart disease. Abbreviations as in Figures 1 and 2.

**Figure 4:**
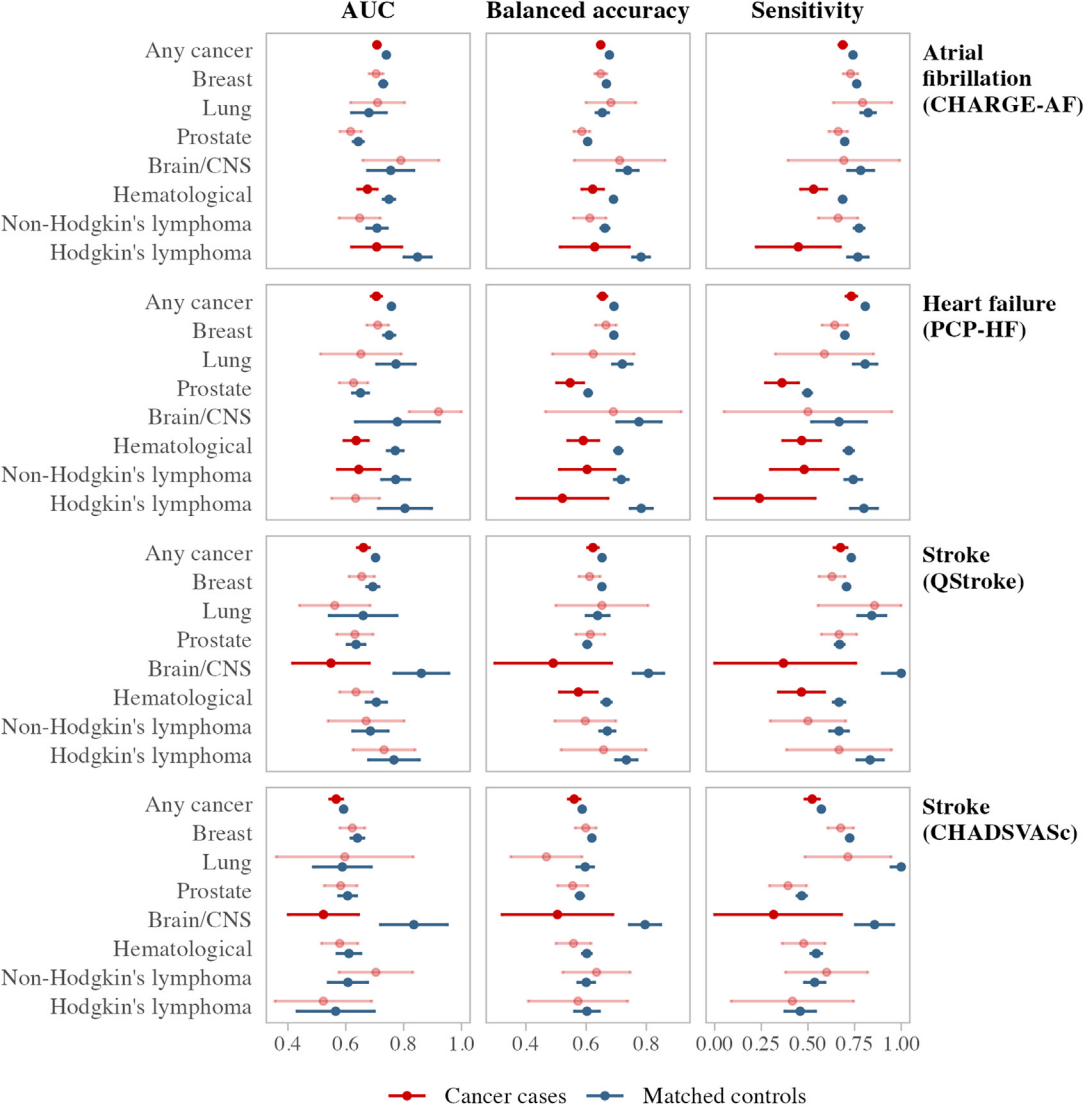
Risk Score Predictive Accuracy in Cancer Groups vs Matched Controls for Specific Outcomes The AUC, balanced accuracy, and sensitivity for atrial fibrillation, heart failure, and stroke are compared between cancer groups and their matched control cohorts. Different outcome–risk score pairs are shown down the right-hand side, and different cancer groups are shown on the left-hand side. Predictive performance results for cancer survivors are displayed in red, with matched controls in dark blue. Cancer groups not significantly different from their noncancer control group are shown in the lighter color. AUC results are from time-dependent analyses accounting for the competing risk of death at the 10-year follow-up. Balanced accuracy and sensitivity are from static performance scoring of 10-year outcomes, with 95% CIs derived from bootstrapping and permutation testing with 1,000 replicates. Abbreviations as in Figures 1 and 2.

Across all risk scores and outcomes, participants with cancer had significantly lower AUC than matched controls, even though this difference was small (2-5 percentage points) (Supplemental Table 7, Figure 2, Figure 3, Figure 4, Central Illustration). These differences were also present in balanced accuracy and sensitivity for all outcomes. The sensitivity of PCP-HF in predicting heart failure was 8 percentage points lower in the cancer group compared with matched controls (73% vs 81%).

**Central Illustration:**
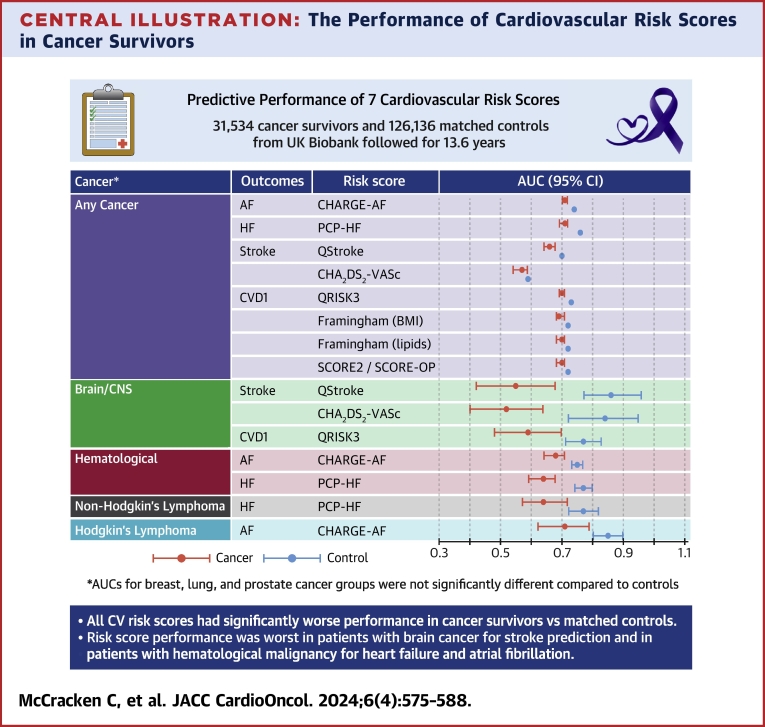
The Performance of Cardiovascular Risk Scores in Cancer Survivors The area under the curve (AUC) for the best-performing risk score in cancer survivors. AF = atrial fibrillation; BMI = body mass index; CNS = central nervous system; CV = cardiovascular; CVD = cardiovascular disease; HF = heart failure.

Cumulative incidence plots (Supplemental Figure 4) confirmed the small but significant differences in cumulative outcome incidence over time. Calibration plots (Supplemental Figure 5) confirm that, in general, SCORE2/OP had the best calibration for CVD 1 in our sample, whereas QRISK3 had the best calibration for CVD 2. Risk score values for CHARGE-AF tended to underestimate the risk for AF, whereas PCP-HF and QStroke tended to overestimate heart failure and stroke outcomes.

#### Breast cancer

In the breast cancer group (Supplemental Table 8, Figure 1), there were no significant differences in 10-year cumulative incidence or risk score accuracy between cases and controls for CVD 1 and stroke. Breast cancer cases had slightly higher incidences of CVD 2, AF, and heart failure than controls (<1.5 percentage points). When predicting CVD 2, the AUCs for breast cancer cases were consistently lower than their matched controls for all risk scores considered, with lower balanced accuracy and sensitivity observed with QRISK3, FRS (blood), and SCORE/OP (Figure 3, Supplemental Table 8). SCORE2/OP and QRISK3 were the best performing risk scores in breast cancer survivors (for CVD 1 and CVD 2, respectively), although AUC failed to reach the 0.70 mark.

#### Lung cancer

In the lung cancer group (Supplemental Table 9), we observed significantly higher 10-year incidences of CVD 2, AF, and heart failure in cancer cases compared with controls, but there were no significant differences in the time-dependent AUC. Although FRS (with BMI) provided the best absolute CVD 2 calibration for lung cancer cases, it provided significantly lower balanced accuracy and sensitivity for CVD 1 and CVD 2 than controls. SCORE2/OP had the highest sensitivity and AUC for discriminating CVD 2 among lung cancer; however, calibration results showed that the absolute SCORE2/OP value underestimated the observed risk by 15 percentage points. Other risk scores had overall comparable performances in lung cancer cases and matched controls (Figure 2, Figure 3, Figure 4).

#### Prostate cancer

In the prostate cancer group (Supplemental Table 10), there were no significant differences in cumulative incidence rates between cases and controls (Figure 1), and there were no significant differences in AUC between cancer cases and matched controls in predicting the outcomes considered (Supplemental Table 10, Figure 2, Figure 3, Figure 4). Although SCORE2/OP had the best calibration for predicting CVD 1 in prostate cancer, sensitivity in cancer cases was significantly lower than in matched controls. Sensitivity for prostate cancer cases was also significantly lower when predicting heart failure with PCP-HF.

#### Brain/CNS cancer

We observed significantly higher rates of CVD 1 and CVD 2 (6.4 and 7.1 percentage points, respectively) in participants with brain/CNS cancer (Central Illustration), even after adjusting for the competing risk of death (Supplemental Table 11). FRS (blood) provided the best overall performance for predicting CVD 2 in brain/CNS cancer cases, with better calibration and sensitivity than SCORE2/OP and QRISK3.

We also observed a significantly higher rate of stroke in brain/CNS cancer cases (7.5% [95% CI: 4.4%-11.2%] vs 0.7% [95% CI: 0.3%-1.4%]). Although both CHA_2_DS_2_VASc and QStroke discriminated stroke well in the control group, AUC for both scores was significantly poorer for brain/CNS cancer cases (AUC ≤55%) along with significantly poorer balanced accuracy and sensitivity. QStroke underestimated the risk for stroke after brain cancer by 4.2 percentage points in our sample.

#### Hematologic malignancies

Among participants with hematologic cancer (Supplemental Table 12, Figure 1), we observed significantly higher cumulative incidence rates for all outcomes considered, even after adjusting for the competing risk of death. Incidences of CVD 1, AF, and heart failure were higher by 4 percentage points, whereas incidence of CVD 2 was 10.6 percentage points higher than in covariate-matched controls. Because of this, even though SCORE2/OP was the best overall score for predicting CVD 1, it significantly underestimated CVD 1 risk by 3.5 percentage points. CVD 2 accuracy for all risk scores and all metrics was significantly poorer for participants with hematologic cancer than their matched controls. This was also the case for AF (CHARGE-AF) and heart failure (PCP-HF) (Figure 4, Central Illustration).

#### Non-Hodgkin lymphoma

Participants with non-Hodgkin lymphoma (Supplemental Table 13) had a higher incidence of CVD 2 (5.7 percentage points) than in matched controls. In addition, there were several significantly lower values for AUC, balanced accuracy, and sensitivity across the various CVD 2 risk scores. The best performing score in our sample profiling CVD 2 in non-Hodgkin lymphoma was FRS (BMI), although AUC is modest at 0.61.

Significant differences were also present in the estimation of heart failure (PCP-HF) in people with non-Hodgkin lymphoma, with significantly poorer AUC, balanced accuracy, and sensitivity than covariate-matched controls (Figure 4, Central Illustration).

#### Hodgkin lymphoma

Participants with Hodgkin lymphoma had higher rates for CVD 1, CVD 2, AF, and heart failure than controls, with the CVD 2 rate 16.5 percentage points higher (Supplemental Table 14). All the scores we tested had very poor calibration in this cancer group, with FRS (BMI) being the best option with an AUC of 0.67 and risk underestimate of 9.4 percentage points. Cancer cases had significantly lower balanced accuracy and sensitivity in all risk scores predicting CVD 2, which was also the case with heart failure. Risk score performance was even poorer when predicting AF (CHARGE-AF), with significantly lower AUC, balanced accuracy, and sensitivity than matched controls.

#### Other hematologic malignancies

We were underpowered to distinguish differences in analyses among more granular hematologic subtypes (Supplemental Tables 15 to 18). Group-specific cumulative death risk curves are provided in Supplemental Figure 6.

## Discussion

### Summary of findings

In this large population-based study, we compared the predictive accuracy of 7 widely validated cardiovascular risk scores in UK Biobank participants with different types of cancer and matched noncancer controls. All the cardiovascular risk scores considered had poorer predictive performance in cancer survivors than in matched noncancer controls. The disparities in performance appeared more important for different cancer types.

There were significant deficits in risk score performance among participants with hematologic malignancies, with the greatest disparities in the prediction of nonischemic outcomes, raising major concerns about the underestimation of risk in these patient groups. For example, there was a 10 percentage point lower prediction accuracy for the prediction of heart failure in hematologic cancer cases compared with the matched controls. Similar poor performance was observed for the outcomes of AF, CVD 2, and stroke in this cohort.

Among participants with past brain/CNS malignancies, the existing risk scores for stroke prediction performed very poorly. Both QStroke and CHA_2_DS_2_-VASc had a more than 30 percentage point lower predictive accuracy in brain/CNS cancer cases than controls, and both demonstrated very poor AUCs (0.52 and 0.55). Overall, standard risk scores appear to be inappropriate for stroke risk estimation in people with a history of brain/CNS cancer.

The performance of risk scores was mostly comparable among participants with prostate cancer and their matched controls, although there was lower sensitivity in the prediction of heart failure and ischemic (CVD 1) outcomes.

### Comparison with existing literature

In an analysis of the National Health and Nutrition Examination Survey, Zhang et al^21^ found significantly higher 10-year risk of atherosclerotic CVD among 1,604 individuals with cancer compared with 13,491 individuals without a history of cancer. These findings were corroborated by So et al,^22^ who demonstrated significantly higher FRS in 1,225 cancer survivors compared with 5,196 noncancer controls. These observations are not surprising and reflect the increased cardiometabolic burden in people with a history of cancer.

The intentional and extensive matching procedure in our study produced near identical risk score distributions for cases and controls across all cancer groups. Therefore, the greater cardiovascular incidence rates observed in our study indicate excess risk conferred by cancer-specific exposures. These findings are consistent with a previous UK Biobank analysis and an England-wide analysis of multiple electronic health record databases, both demonstrating elevated long-term CVD risk in cancer survivors compared with noncancer controls independent of shared risk factors.^5^^,^^6^

Previous studies of breast^23^ and childhood cancer survivors^24^ have reported similar 10-year cardiovascular risk estimates in cases and controls. The present analysis significantly extends these observations by demonstrating that comparable cardiovascular risk scores in cancer and noncancer individuals do not necessarily reflect equivalent long-term risk observed in health records. This is a vitally important conclusion and underscores the need for caution in interpreting traditional cardiovascular risk estimates and their propensity to underestimate risk in cancer survivors. Decisions to initiate preventive cancer therapies in such cohorts must consider these limitations of existing risk scores, and a lower threshold for starting such interventions may be reasonable in particularly vulnerable cohorts (eg, hematologic cancer survivors).

Law et al^25^ found that the FRS significantly underestimated cardiovascular risk in 152 HER2-positive breast cancer patients over 40 months of follow-up. This short-term study did not include a noncancer comparison, and the endpoints considered were poorly defined and lay outside the original remit of the FRS (eg, pericardial disease and enlarged abdominal aortic aneurysm). In our analysis, we found comparable predictive performance for FRS between breast cancer survivors and their matched controls when defining the CVD endpoint as originally intended (CVD 1), excluding participants with CVD events before recruitment, and comparing outcomes over a much longer period of prospective follow-up. However, we demonstrate significant deficits in predicting the extended CVD 2 outcomes (including a broad composite of ischemic and nonischemic CVD) in participants with breast cancer compared with noncancer controls, which appeared consistent across the FRS, QRISK3, and SCORE/SCORE-OP tools.

In a recent analysis, Tawfiq et al^26^ evaluated performance of the New Zealand CVD risk prediction equations in 14,263 cancer survivors, reporting reasonable performance of these risk scores. The authors did not consider other cardiovascular risk scores and did not report performance by cancer subcategories. Furthermore, they do not compare score performance between cancer and noncancer groups. Our analysis adds to this report by providing information about the performance of 7 internationally established cardiovascular risk scores across separate cancer types, with comparison of performance against an extensively matched noncancer group. In doing so, we identify cancer survivors who are most susceptible to cardiovascular risk underestimation and provide widely applicable insights for clinical practice.

The variations in predictive accuracy observed across cancer groups likely reflect differences in specific cardiotoxicity exposures and their relative importance alongside traditional risk factors. For instance, hematologic cancer patients are often treated with anticancer therapies with high cardiotoxicity potential, such as anthracyclines and mediastinal radiotherapy,^4^ driving a higher risk of CVD through mechanisms independent of traditional cardiometabolic factors. The importance of cardiotoxic treatment exposures is likely amplified in hematologic cancers that have a chronic course, such as chronic myeloid leukemia or Hodgkin lymphoma, which typically involve prolonged and repeated exposure to systemic anticancer therapies.

Another potential driver of heightened cardiovascular risk in cancer survivors is clonal hematopoiesis of indeterminate potential sequence variants, which are increasingly recognized as a shared risk factor for both cancer (particularly leukemia) and CVD.^27^ The impact of these exposures on cardiovascular risk is even more significant in cohorts in which traditional risk factors are less prevalent.

In our study, lung and prostate cancer survivors (and their respective matched controls) were older and generally more comorbid compared with other cancer subtypes, leading to higher absolute risks of cardiovascular outcomes. Therefore, although independent cancer-specific risk factors may influence the risk of cardiovascular outcomes, the contribution of these is less apparent given the high baseline risk. In contrast, hematologic cancer patients were younger and less comorbid, and the contribution of cancer-specific risk factors was more pronounced, leading to significant underperformance of most existing cardiovascular risk scores.

The greater disparities in risk score performance for nonischemic outcomes (eg, heart failure and AF) are not unexpected and may reflect differential (treatment-related) etiologies of these outcomes in cancer survivors compared with profile-matched noncancer controls.

Our findings highlight important concerns about the use of standard risk scores for estimating stroke risk in patients with past brain/CNS cancer. This observation likely reflects the high risk of intracerebral bleed as a direct complication of both cancer and its treatments in this cohort, which far exceed in importance the factors that increase the risk of stroke in the general population.

Our findings advocate an urgent need for the development and validation of cardiovascular risk scores in cancer survivors. As a first step toward this, Strongman et al^28^ evaluated the utility of cancer history (solid cancer, hematologic cancer, or cancer free) as a categoric predictor variable in QRISK3, demonstrating that hematologic cancer meets the threshold for inclusion in men and women and solid cancer meets the threshold in men but not in women. Given the considerable heterogeneity in demographics and cardiotoxicity exposures, it is likely that future risk score development would benefit from bespoke handling for each cancer type.

### Clinical take-home messages: choice of risk estimates

In the absence of dedicated risk scores, the following recommendations may be considered based on our findings when estimating risk in people with a history of cancer:

• Any heart disease: when considering the risk of any CVD outcome (ie, CVD 2), the FRS is the best choice for brain/CNS and hematologic malignancies, including Hodgkin lymphoma and non-Hodgkin lymphoma, whereas QRISK3 is the best choice in breast and prostate cancer survivors.
• Ischemic heart disease: for ischemic cardiovascular outcomes (ie, CVD 1), SCORE2/OP represents the best overall performance across most cancer types.
• AF: CHARGE-AF was less accurate in predicting future AF risk in cancer survivors generally, with the greatest deficiencies in hematologic cancer groups, especially Hodgkin lymphoma.
• Stroke: QStroke offers better overall predictive performance for stroke outcomes among breast, prostate, and hematologic malignancies (composite category) and Hodgkin lymphoma. In non-Hodgkin lymphoma, CHA_2_DS_2_-VASc has better calibration than QStroke and may be preferrable. Among brain/CNS cancer patients, although CHA_2_DS_2_-VASc has somewhat better performance than QStroke, both tools had very poor metrics, and their use for stroke prediction in this setting is not recommended based on our results.
• Heart failure: although PCP-HF is a very well-performing score in noncancer controls, it had poorer metrics in cancer survivors (any cancer) and significant underestimation of risk in hematologic cancer survivors, particularly those with Hodgkin lymphoma.

### Strengths and limitations

The detailed participant phenotyping and health record linkage in the UK Biobank enabled faithful replication of a range of cardiovascular risk scores and prospective tracking of incident events. However, healthy participant and survival bias may have influenced our sample and observed risks. Further studies in nationally representative cohorts are needed to evaluate the generalizability of our observations.

The data set did not permit reliable distinction of individuals who may have received active cancer treatment during the study follow-up period for recurrent or secondary malignancies, which may represent a particularly high-risk cohort. Similarly, cardiovascular risk prediction may be worse in people with past exposure to cardiotoxic therapies; the absence of this information in the current data set precludes evaluation of this hypothesis in the current analysis and represents an important priority for future research.

The 94% 10-year survival rate is not consistent with the average 10-year survival of patients with most newly diagnosed cancers among the types included in this paper. This discrepancy suggests potential circumstantial evidence of immortal time bias. It is plausible that this bias favors the performance of the risk scores evaluated in this paper; in other words, these scores may perform even worse in patients with newly diagnosed cancer.

## Conclusions

Our findings underscore the heightened long-term risk of CVD in cancer survivors, which are independent of shared risk factors and incompletely captured by existing risk scores. The deficits in risk assessment of hematologic cancer survivors have important implications for clinical practice and research.

Perspectives **COMPETENCY IN MEDICAL KNOWLEDGE:** Existing established cardiovascular risk scores underestimate risk in cancer survivors, particularly individuals with past hematologic malignancies.

**TRANSLATIONAL OUTLOOK:** The interpretation of risk scores should be made with caution in cancer survivors, mindful of their unknown validity in this cohort and propensity to underestimate risk.

## Funding Support and Author Disclosures

Dr McCracken and Prof Neubauer are supported by the Oxford National Institute for Health and Care Research Biomedical Research Centre (IS-BRC-1215-20008). Prof Neubauer is additionally supported by the Oxford British Heart Foundation Centre of Research Excellence. Drs Condurache (G-002530) and Szabo (G-002389) were supported by the Barts Charity. This work was supported by the National Institute for Health and Care Research Barts Biomedical Research Centre (NIHR203330), a delivery partnership of Barts Health NHS Trust, Queen Mary University of London, St George’s University Hospitals NHS Foundation Trust and St George’s University of London. Dr Szabo and Prof Petersen have received funding from the European Union’s Horizon 2020 research and innovation program under grant agreement no. 825903 (euCanSHare project). Prof Walter is codirector of the CanTest Collaborative, which is funded by Cancer Research UK (CC8640/A23385). Prof Mead is funded by a CRUK Senior Cancer Research Fellowship (grant number C42639/A26988). Prof Manisty is supported directly and indirectly from the National Institute for Health and Care Research Biomedical Research Centres at University College London Hospitals and Barts Health NHS Trusts. Prof Harvey is supported by the UK Medical Research Council (MC_PC_21003; MC_PC_21001) and National Institute for Health and Care Research Southampton Biomedical Research Centre, University of Southampton and University Hospital Southampton NHS Foundation Trust. Dr Raisi-Estabragh recognizes the National Institute for Health and Care Research Integrated Academic Training program (CL-2021-19-00), which supports her academic clinical lectureship post, and was also supported by British Heart Foundation Clinical Research Training Fellowship no. FS/17/81/33318. The funders of the study had no role in the study design, data collection, data analysis, data interpretation, or decision to publish. The authors were not precluded from accessing data in the study, and they accept responsibility to submit for publication. Dr Petersen is as a consultant to Cardiovascular Imaging Inc. All other authors have reported that they have no relationships relevant to the contents of this paper to disclose.
