# Supplementary material for: Predictive Performance of Cardiovascular Risk Scores in Cancer Survivors From the UK Biobank
Source: JACC CardioOncol. 2024 Jul 23;6(4):575–88. doi: 10.1016/j.jaccao.2024.05.015 (PMC11372025; doi:10.1016/j.jaccao.2024.05.015)
Supplement: Supplemental Material [file mmc1.docx]

**Supplemental Material**

**Predictive performance of cardiovascular risk scores in cancer survivors from the UK Biobank**

Supplemental [Table 1: Condition definitions in UK Biobank 2](#_Toc160817150)

Supplemental [Table 2: Risk scores and their components 6](#_Toc160817151)

Supplemental [Table 3: Imputation report 9](#_Toc160817152)

Supplemental [Figure 1: Cohort matching summary 10](#_Toc160817153)

Supplemental [Figure 2: Sample selection 11](#_Toc160817154)

Supplemental [Table 4: Full descriptive statistics for cancer groups and their Controls (solid tumours) 13](#_Toc160817155)

Supplemental [Table 5: Full descriptive statistics for cancer groups and their Controls (haematological) 14](#_Toc160817156)

Supplemental [Table 6: Risk score statistical summaries 15](#_Toc160817157)

Supplemental [Table 7: Any cancer 18](#_Toc160817158)

Supplemental [Figure 3: Cumulative Incidence Function Plot- Any cancer 19](#_Toc160817159)

Supplemental [Figure 4: Calibration plots – Any cancer 20](#_Toc160817160)

Supplemental [Table 8: Breast cancer 21](#_Toc160817161)

Supplemental [Table 9: Lung cancer 22](#_Toc160817162)

Supplemental [Table 10: Prostate cancer 23](#_Toc160817163)

Supplemental [Table 11: Brain/CNS cancer 24](#_Toc160817164)

Supplemental [Table 12: Haematological cancer 25](#_Toc160817165)

Supplemental [Table 13: Non-Hodgkin’s lymphoma 26](#_Toc160817166)

Supplemental [Table 14: Hodgkin’s lymphoma 27](#_Toc160817167)

Supplemental [Table 15: Multiple myeloma 28](#_Toc160817168)

Supplemental [Table 16: Leukaemia 29](#_Toc160817169)

Supplemental [Table 17: Polycythaemia vera 30](#_Toc160817170)

Supplemental [Table 18: Myelodysplastic syndrome 31](#_Toc160817171)

##

## Supplemental Table 1: Condition definitions in UK Biobank

| Condition | Source | UK Biobank Field ID or code |
| --- | --- | --- |
| Venous | Self-report (20002) | 1068, 1093, 1094 |
| thromboembolism | ICD10 | I260, I269, I801, I802, I803, I808, I809, I828, I829, O082, O223, O229, O871, O879, O882 |
|  | ICD9 | 4151, 4511, 4512, 4518, 4519, 4538, 4539, 6346, 6356, 6366, 6376, 6386, 673 |
|  | Diagnosed by doctor (6150, 6152) | 4012, 4022, 5, 7 |
|  | First occurrences | 131396 |
|  | OPCS4 | L124, L131, L712, L791, L794, L795, L901, L902, L908, L909, L961, L962, L968, L969, L994 |
| Erectile dysfunction | Self-report (20002) | 1518 |
|  | ICD10 | F522, N484 |
|  | Medications (20003) | 1140869100, 1140883010, 1141168936, 1141168944, 1141168946, 1141168948, 1141187810, 1141187814, 1141187818, 1141192248, 1141192256, 1141192258, 1141192260 |
| Stroke | Algorithm | 42006 |
|  | Self-report (20002) | 1081, 1086, 1491, 1583 |
|  | ICD10 | I60, I61, I63, I64 |
|  | ICD9 | 430, 431, 434, 436 |
|  | Diagnosed by doctor (6150, 6152) | 3, 4056 |
|  | First occurrences | 131360, 131362, 131366, 131368 |
| Transient ischaemic | Self-report (20002) | 1082 |
| attack | ICD10 | G45 |
|  | ICD9 | 435 |
|  | First occurrences | 131056 |
| Migraine | Self-report (20002) | 1265 |
|  | ICD10 | G43 |
|  | ICD9 | 346 |
|  | First occurrences | 131052 |
| Ischaemic heart | Algorithm | 42000 |
| disease | Self-report (20002) | 1074, 1075 |
|  | Self-report (20004) | 1070, 1095, 1523 |
|  | ICD10 | I20, I21, I22, I23, I24, I25 |
|  | ICD9 | 410, 411, 412, 413, 414, 429 |
|  | Diagnosed by doctor (6150, 6152) | 1, 2, 3627, 3894 |
|  | First occurrences | 131296, 131298, 131300, 131302, 131304, 131306 |
|  | OPCS4 | K40, K41, K42, K43, K44, K45, K46, K47, K48, K49, K50, K51, K75 |
| Myocardial | Algorithm | 42000 |
| infarction | Self-report (20002) | 1075 |
|  | ICD10 | I21, I22, I23, I241, I252 |
|  | ICD9 | 410, 411, 412, 429 |
|  | Diagnosed by doctor (6150, 6152) | 1, 3894 |
|  | First occurrences | 131298, 131300, 131302 |
| Cardiac arrhythmia | Self-report (20002) | 1077, 1471, 1483, 1485, 1486, 1487 |
|  | Self-report (20004) | 1096, 1550, 1551 |
|  | ICD10 | I44, I45, I46, I47, I48, I49 |
|  | ICD9 | 426, 427 |
|  | First occurrences | 131342, 131344, 131346, 131348, 131350, 131352 |
|  | OPCS4 | K60, K61, K73, K74, U31 |
| Atrial fibrillation | Self-report (20002) | 1471 |
|  | ICD10 | I48, I480, I481, I482, I489 |
|  | ICD9 | 4273 |
|  | First occurrences | 131350 |
| Non-ischaemic | Self-report (20002) | 1079, 1588 |
| cardiomyopathies | ICD10 | I11, I13, I42, I43 |
|  | ICD9 | 4020, 4021, 4029, 4040, 4041, 4049, 4250, 4253, 4255 |
|  | First occurrences | 131288, 131292, 131338, 131340 |
| Valvular heart | Self-report (20002) | 1078, 1489, 1490, 1584, 1585, 1586, 1587 |
| disease | ICD10 | I05, I06, I07, I08, I34, I35, I36, I37, I38, I39 |
|  | First occurrences | 131276, 131278, 131280, 131282, 131322, 131324, 131326, 131328, 131330, 131332 |
| Heart failure | Self-report (20002) | 1076 |
|  | ICD10 | I500, I501, I509 |
|  | ICD9 | 4280, 4281, 4289 |
|  | First occurrences | 131354 |
| Chronic kidney | Algorithm | 42026 |
| disease | Self-report (20002) | 1193 |
|  | Self-report (20004) | 1195, 1580, 1581, 1582 |
|  | ICD10 | N165, N180, N183, N184, N185, N188, N189, T824, T861, Y602, Y612, Y622, Y841, Z490, Z491, Z492, Z940, Z992 |
|  | ICD9 | 585, 5859 |
|  | OPCS4 | L741, L742, L743, L744, L745, L746, L748, L749, M012, M013, M014, M015, M018, M019, M023, M084, M172, M174, M178, M179, X401, X402, X403, X404, X405, X406, X407, X408, X409, X411, X412, X418, X419, X421, X428, X429, X431 |
| Severe or recurrent | ICD10 | F322, F330, F331, F332, F334, F338, F339 |
| depression | ICD9 | 3091 |
|  | First occurrences | 130896 |
| Serious mental | Self-report (20002) | 1243, 1289, 1291 |
| illness | ICD10 | F20, F23, F28, F29, F31, F323, F333 |
|  | ICD9 | 295, 296 |
|  | First occurrences | 130874, 130880, 130886, 130888, 130892 |
|  | Self-report (20544) | 10, 2, 3 |
| Atypical antipsychotic medication | Medications (20003) | 1140867420, 1140867432, 1140867444, 1140927956, 1140927970, 1140928916, 1141152848, 1141152860, 1141153490, 1141167976, 1141177762, 1141195974, 1141202024 |
| Cancers |  |  |
| Oral cavity | ICD10 | C00, C000, C001, C002, C003, C004, C005, C006, C009, C01, C02, C020, C021, C022, C023, C024, C028, C029, C03, C030, C031, C039, C04, C040, C041, C048, C049, C05, C050, C051, C052, C058, C059, C06, C060, C061, C062, C068, C069, C07, C08, C080, C081, C088, C089, C09, C090, C091, C098, C099, C10, C100, C102, C103, C104, C108, C109, C11, C110, C111, C112, C113, C119, C12, C13, C130, C131, C132, C138, C139, C14, C140, C148, D000, D370 |
|  | ICD9 | 141, 142, 144, 1413, 1416, 1419, 1420, 1440, 2300 |
|  | Cancer register (40006) | C00, C01, C02, C03, C04, C05, C06, C07, C08, C09, C10, C11, C12, C13, C14 |
|  | Self-report (20001) | 1005, 1010, 1011, 1012, 1077 |
| Oesophageal | ICD10 | C15, C150, C151, C152, C153, C154, C155, C158, C159, D001 |
|  | ICD9 | 150, 1505 |
|  | Cancer register (40006) | C15 |
|  | Self-report (20001) | 1017 |
| Stomach | ICD10 | C16, C160, C161, C162, C163, C164, C165, C166, C168, C169, D002, D371 |
|  | ICD9 | 151, 1519 |
|  | Cancer register (40006) | C16 |
|  | Self-report (20001) | 1018 |
| Small intestine | ICD10 | C17, C170, C171, C172, C173, C178, C179, D372 |
|  | ICD9 | 152, 1521 |
|  | Cancer register (40006) | C17 |
|  | Self-report (20001) | 1019 |
| Colorectal | ICD10 | C18, C180, C181, C182, C183, C184, C185, C186, C187, C188, C189, C19, C20, D010, D011, D012, D374, D375 |
|  | ICD9 | 153, 154, 1530, 1532, 1533, 1534, 1536, 1537, 1539, 1540, 1541 |
|  | Cancer register (40006) | C18, C19, C20 |
|  | Self-report (20001) | 1020, 1022, 1086 |
| Liver | ICD10 | C22, C220, C221, C223, C224, C227, C229, D015, D376 |
|  | ICD9 | 155, 1551 |
|  | Cancer register (40006) | C22 |
|  | Self-report (20001) | 1024 |
| Pancreas | ICD10 | C25, C250, C251, C252, C253, C254, C257, C258, C259 |
|  | ICD9 | 157, 1570, 1574, 1579 |
|  | Cancer register (40006) | C25 |
|  | Self-report (20001) | 1026 |
| Anus/anal canal | ICD10 | C21, C210, C211, C218, D013 |
|  | ICD9 | 1542, 1543 |
|  | Cancer register (40006) | C21 |
|  | Self-report (20001) | 1021, 1023 |
| Lung | ICD10 | C34, C340, C341, C342, C343, C348, C349 |
|  | ICD9 | 162, 1623, 1629 |
|  | Cancer register (40006) | C34 |
|  | Self-report (20001) | 1001, 1027, 1028 |
| Malignant melanoma | ICD10 | C43, C430, C431, C432, C433, C434, C435, C436, C437, C438, C439, D03, D030, D031, D032, D033, D034, D035, D036, D037, D038, D309 |
|  | ICD9 | 172, 1720, 1723, 1725, 1726, 1727, 1729, 2325, 2326, 2327 |
|  | Cancer register (40006) | C43, D03 |
|  | Self-report (20001) | 1003, 1059 |
| Breast | ICD10 | C50, C500, C501, C502, C503, C504, C505, C506, C508, C509, D05, D050, D051, D057, D059, D486 |
|  | ICD9 | 174, 1740, 1743, 1744, 1745, 1748, 1749, 2330 |
|  | Cancer register (40006) | C50, D05 |
|  | Self-report (20001) | 1002 |
| Cervix | ICD10 | C53, C530, C531, C538, C539, D06, D060, D061, D067, D069 |
|  | ICD9 | 180, 1800, 1809, 2331 |
|  | Cancer register (40006) | C53, D06 |
|  | Self-report (20001) | 1041 |
| Uterus | ICD10 | C54, C540, C541, C542, C543, C548, C549, C55, D390 |
|  | ICD9 | 179, 182, 1799, 1820, 2332 |
|  | Cancer register (40006) | C54, C55 |
|  | Self-report (20001) | 1040 |
| Ovary | ICD10 | C56, D391 |
|  | ICD9 | 183, 1830 |
|  | Cancer register (40006) | C56 |
|  | Self-report (20001) | 1039 |
| Prostate | ICD10 | C61, D075, D400 |
|  | ICD9 | 185, 1859 |
|  | Cancer register (40006) | C61 |
|  | Self-report (20001) | 1044 |
| Testis | ICD10 | C62, C620, C621, C629, D401 |
|  | ICD9 | 186, 1860, 1869 |
|  | Cancer register (40006) | C62 |
|  | Self-report (20001) | 1045 |
| Kidney | ICD10 | C64, D410, D411 |
|  | ICD9 | 189, 1890 |
|  | Cancer register (40006) | C64 |
|  | Self-report (20001) | 1034 |
| Bladder | ICD10 | C67, C670, C671, C672, C673, C674, C675, C676, C677, C678, C679, D090, D413, D414 |
|  | ICD9 | 188, 1882, 1889, 2337 |
|  | Cancer register (40006) | C67 |
|  | Self-report (20001) | 1035 |
| Brain/CNS | ICD10 | C71, C710, C711, C712, C713, C714, C715, C716, C717, C718, C719, C72, C720, C721, C722, C724, C725, C728, C729 |
|  | ICD9 | 191, 1913, 1916, 1919 |
|  | Cancer register (40006) | C71, C72 |
|  | Self-report (20001) | 1031, 1032, 1033 |
| Thyroid | ICD10 | C73, D093, D440 |
|  | ICD9 | 193, 1939 |
|  | Cancer register (40006) | C73 |
|  | Self-report (20001) | 1065, 1066 |
| Non-Hodgkin’s  lymphoma | ICD10 | C82, C820, C821, C822, C823, C824, C825, C826, C827, C829, C83, C830, C831, C832, C833, C834, C835, C836, C837, C838, C839, C84, C840, C841, C843, C844, C845, C846, C847, C848, C849, C85, C850, C851, C852, C857, C859, C86, C860, C862, C863, C864, C865, C866 |
|  | ICD9 | 2020, 2028 |
|  | Cancer register (40006) | C82, C83, C84, C85, C86 |
|  | Self-report (20001) | 1053 |
| Hodgkin’s | ICD10 | C81, C810, C811, C812, C813, C814, C817, C819 |
| lymphoma | ICD9 | 201, 2015, 2016, 2017, 2019 |
|  | Cancer register (40006) | C81 |
|  | Self-report (20001) | 1052 |
| Multiple | ICD10 | C90, C900, C901, C902, C903 |
| myeloma | ICD9 | 203 |
|  | Cancer register (40006) | C90 |
|  | Self-report (20001) | 1050 |
| Leukaemia | ICD10 | C91, C910, C911, C912, C913, C914, C915, C916, C917, C918, C919, C92, C920, C921, C922, C923, C924, C925, C926, C927, C928, C929, C93, C930, C931, C94, C940, C942, C944, C945, C946, C947, C95, C950, C951, C959 |
|  | ICD9 | 204, 205, 2040, 2050, 2051, 2059 |
|  | Cancer register (40006) | C91, C92, C93, C94, C95 |
|  | Self-report (20001) | 1048, 1055, 1056, 1074 |
| Myelodysplastic syndrome | ICD10 | D46, D460, D461, D462, D463, D464, D465, D466, D467, D469, D473 |
|  | ICD9 | 238.75 |
|  | Cancer register (40006) | D46 |
|  | Self-report (20001) | 1051 |
| Metastatic | ICD10 | C77, C770, C771, C772, C773, C774, C775, C778, C779, C78, C780, C781, C782, C783, C784, C785, C786, C787, C788, C79, C790, C791, C792, C793, C794, C795, C796, C797, C798, C799 |
|  | ICD9 | 196, 198, 1983 |
|  | Cancer register (40012) | 6, 9 |
|  | Self-report (20001) | 1071, 1085 |
| Diabetes | Self-report (20002) | 1220, 1222, 1223 |
|  | ICD10 | E10, E11, E13, E14, G590, G632, H280, H360, M142, N083, O240, O241, O243, O244, O249, Y423 |
|  | Diagnosed by doctor | 2443, 2976, 2986 |
|  | Medications (20003) | 1140883066 |
|  | Medications (6153, 6177) | 3 |
|  | First occurrences | 130706, 130708, 130712, 130714 |
| Hypertension | Self-report (20002) | 1065, 1072 |
|  | ICD10 | I10 |
|  | Diagnosed by doctor (6150, 6152) | 2966, 4 |
|  | Medications (6153, 6177) | 2 |
|  | First occurrences | 131286 |
| Hypertension medication | Medications (20003) | 1140860334, 1140860336, 1140860338, 1140860340, 1140860342, 1140860348, 1140860352, 1140860356, 1140860358, 1140860380, 1140860382, 1140860386, 1140860390, 1140860394, 1140860396, 1140860398, 1140860402, 1140860404, 1140860406, 1140860410, 1140860418, 1140860422, 1140860426, 1140860434, 1140860492, 1140860498, 1140860562, 1140860564, 1140860580, 1140860590, 1140860610, 1140860654, 1140860658, 1140860690, 1140860696, 1140860706, 1140860714, 1140860728, 1140860736, 1140860738, 1140860750, 1140860752, 1140860758, 1140860764, 1140860776, 1140860784, 1140860790, 1140860802, 1140860806, 1140860878, 1140860882, 1140860892, 1140860904, 1140860912, 1140860918, 1140861088, 1140861090, 1140861106, 1140861110, 1140861114, 1140861120, 1140861128, 1140861130, 1140861136, 1140861138, 1140861166, 1140861176, 1140861190, 1140861194, 1140861202, 1140861276, 1140861282, 1140864950, 1140864952, 1140866072, 1140866078, 1140866090, 1140866092, 1140866094, 1140866096, 1140866102, 1140866104, 1140866122, 1140866128, 1140866132, 1140866136, 1140866138, 1140866140, 1140866144, 1140866146, 1140866156, 1140866158, 1140866162, 1140866164, 1140866168, 1140866226, 1140866232, 1140866236, 1140866244, 1140866262, 1140866306, 1140866308, 1140866312, 1140866318, 1140866324, 1140866328, 1140866330, 1140866340, 1140866352, 1140866354, 1140866360, 1140866396, 1140866400, 1140866402, 1140866404, 1140866410, 1140866416, 1140866420, 1140866440, 1140866446, 1140866450, 1140866460, 1140866466, 1140866484, 1140866546, 1140866554, 1140866692, 1140866704, 1140866712, 1140866724, 1140866726, 1140866738, 1140866756, 1140866758, 1140866764, 1140866766, 1140866778, 1140866782, 1140866784, 1140866798, 1140866800, 1140866802, 1140866804, 1140879760, 1140879762, 1140879778, 1140879782, 1140879786, 1140879794, 1140879798, 1140879802, 1140879806, 1140879810, 1140879818, 1140879824, 1140879830, 1140879834, 1140879842, 1140879866, 1140888510, 1140888552, 1140888556, 1140888560, 1140888578, 1140888646, 1140909368, 1140911698, 1140916356, 1140916362, 1140917428, 1140923572, 1140923712, 1140923718, 1140926778, 1140926780, 1141145658, 1141145660, 1141145668, 1141151016, 1141151018, 1141151382, 1141152600, 1141152998, 1141153006, 1141153026, 1141153032, 1141153328, 1141156754, 1141156808, 1141156836, 1141156846, 1141164148, 1141164154, 1141164276, 1141164280, 1141165470, 1141165476, 1141166006, 1141167822, 1141167832, 1141171152, 1141171336, 1141171344, 1141172682, 1141172686, 1141180592, 1141180598, 1141187788, 1141187790, 1141190160, 1141193282, 1141193346, 1141194794, 1141194800, 1141194804, 1141194808, 1141194810, 1141201038, 1141201040 |
|  | Medications (6153, 6177) | 2 |
| High cholesterol | Self-report (20002) | 1473 |
|  | ICD10 | E780, E782, E783, E784, E785 |
|  | Medications (6153, 6177) | 1 |
|  | First occurrences | 130814 |
| Rheumatoid | Self-report (20002) | 1464 |
| arthritis | ICD10 | M05, M06 |
|  | First occurrences | 131848, 131850 |
| Systemic lupus | Self-report (20002) | 1381 |
| erythematosus | ICD10 | M32 |
|  | ICD9 | 7100 |
|  | First occurrences | 131894 |

**Supplemental Table 1 footnote**: ICD10 codes are drawn from fields 41270, 41280, 41234 and 41259; ICD9 codes are drawn from fields 41271, 41281, 41234 and 41259; OPCS4 codes are drawn from fields 41272, 41282, 41149 and 41259; Primary care codes are drawn from field 42040. Deaths codes are drawn from fields 40000, 40001 and 40023. Where a 3-digit code is given, this includes all 4-digit sub-codes, for example, I46 includes I462, I468 and I469.

## Supplemental Table 2: Risk scores and their components

| **Risk score** | **Components** | **Outcome** | **Intended setting** | **Reference publication** |
| --- | --- | --- | --- | --- |
| QRISK3 | Age, sex, ethnic group, body mass index, smoking category, systolic blood pressure, systolic blood pressure standard deviation, Townsend deprivation index, total  cholesterol, HDL cholesterol, atypical antipsychotics,  regular steroid tablets, migraine, rheumatoid arthritis, atrial  fibrillation, chronic kidney disease (Stage 3,4 or 5), severe mental illness, systemic lupus erythematosus, erectile  dysfunction diagnosis, hypertension medication, Type 1  Diabetes, Type 2 Diabetes, family history of heart disease | Combined  cardiovascular disease  (CVD 1, CVD 2) | Prediction of incident ischaemic heart disease and  stroke over a ten-year period in the absence of  cardiovascular disease at  baseline | Hippisley-Cox et al. (2017)[1] |
| Framingham Risk Score (with BMI) | Age, sex, BMI, systolic blood pressure, hypertension medication, smoker, diabetes |  | Assessment of the 10-year risk of coronary heart disease | D’Agostino et al. (2008)[2] |
| Framingham Risk Score (with blood lipids) | Age, sex, total cholesterol, HDL cholesterol, systolic blood pressure, hypertension medication, smoker, diabetes |  | Assessment of the 10-year risk of coronary heart disease | D’Agostino et al. (2008)[2] |
| SCORE2/ SCORE-OP | Age, sex, smoker, systolic blood pressure, diabetes, total cholesterol, HDL cholesterol |  | Prediction of 10-year risk of fatal and non-fatal cardiovascular disease events without previous CVD or diabetes | SCORE2 working group (2021)[3][4] |
| CHARGE-AF | Age, ethnicity, height, weight, systolic blood pressure, diastolic blood pressure, smoker, hypertension medication,  diabetes, heart failure, myocardial infarction | Atrial fibrillation | Prediction of atrial fibrillation risk | Alonso et al (2013)[5] |
| PCP-HF | Age, sex, ethnic group, body mass index, hypertension, systolic blood pressure, smoker, Type 2 diabetes, serum  glucose, total cholesterol, HDL cholesterol | Heart failure | Sex and race-specific estimation of 10-year risk of incident heart failure | Khan et al. (2019)[6] |
| QStroke | Age, sex, ethnic group, body mass index, smoking category, total cholesterol, HDL cholesterol, systolic blood pressure, Townsend deprivation index, atrial fibrillation,  heart failure, ischaemic heart disease, rheumatoid arthritis, chronic kidney disease (Stage 3,4 or 5), hypertension  medication, Type 1 diabetes, Type 2 diabetes, valvular heart disease, family history of heart disease | Stroke | Prediction of stroke or transient ischaemic attack risk in patients without prior  stroke or transient ischaemic attack at baseline | Hippisley-Cox et al. (2013)[7] |
| CHA_2_DS_2_-VASc | Age, sex, heart failure, hypertension, diabetes, stroke, transient ischemic attack, venous thromboembolism, myocardial infarction, peripheral artery disease |  | Estimation of ischemic stroke risk in patients with  documented atrial fibrillation | Ntaios et al. (2013)[8] |

CVD 1 = combined endpoint including non-fatal myocardial infarction, non-fatal stroke or cardiovascular mortality, where cardiovascular mortality is defined as any death with a primary cause from ICD10 I00-I80. CVD 2 = combined endpoint including everything from CVD 1 plus incident atrial fibrillation, heart failure, non-ischaemic cardiomyopathies and valvular heart disease.

**References for Supplemental Table 2**

1. Hippisley-Cox J, Coupland C, Brindle P. Development and validation of QRISK3 risk prediction algorithms to estimate future risk of cardiovascular disease: Prospective cohort study. BMJ (Online). 2017 May 23;357(May):1–21.
2. D’Agostino RB, Vasan RS, Pencina MJ, Wolf PA, Cobain M, Massaro JM, et al. General Cardiovascular Risk Profile for Use in Primary Care. Circulation. 2008 Feb 12;117(6):743–53.
3. SCORE2-OP working group and ESC Cardiovascular risk collaboration. SCORE2-OP risk prediction algorithms: estimating incident cardiovascular event risk in older persons in four geographical risk regions. European Heart Journal. 2021 Jul 1;42(25):2455–67.
4. SCORE2 working group and ESC Cardiovascular risk collaboration. SCORE2 risk prediction algorithms: new models to estimate 10-year risk of cardiovascular disease in Europe. European Heart Journal. 2021 Jul 1;42(25):2439–54.
5. Alonso A, Krijthe BP, Aspelund T, Stepas KA, Pencina MJ, Moser CB, et al. Simple risk model predicts incidence of atrial fibrillation in a racially and geographically diverse population: the CHARGE-AF consortium. Journal of the American Heart Association [Internet]. 2013 Mar 18 [cited 2022 Feb 14];2(2). Available from: <https://www.ahajournals.org/doi/abs/10.1161/JAHA.112.000102>
6. Khan SS, Ning H, Shah SJ, Yancy CW, Carnethon M, Berry JD, et al. 10-Year Risk Equations for Incident Heart Failure in the General Population. Journal of the American College of Cardiology. 2019 May;73(19):2388–97.
7. Hippisley-Cox J, Coupland C, Brindle P. Derivation and validation of QStroke score for predicting risk of ischaemic stroke in primary care and comparison with other risk scores: a prospective open cohort study. BMJ. 2013 May 2;346:f2573.
8. Ntaios G, Lip GYH, Makaritsis K, Papavasileiou V, Vemmou A, Koroboki E, et al. CHADS2, CHA_2_DS_2_-VASc, and long-term stroke outcome in patients without atrial fibrillation. Neurology. 2013 Mar 12;80(11):1009–17.

## Supplemental Table 3: Imputation report

| **Variable** | **Count missing** | **Percent missing**  **(in 157,670)** | **Strategy** |
| --- | --- | --- | --- |
| Townsend Deprivation Index | 163 | 0.10% | Replaced with mean |
| Ethnicity | 702 | 0.45% | Replaced with majority category (White) |
| Smoking | 259 | 0.16% | Replaced with majority category (Non-smoker) |
| Physical activity (MET-minutes per week) | 1,009 | 0.64% | MICE |
| Systolic blood pressure (SBP) | 188 | 0.12% | MICE |
| SBP standard deviation | 5,135 | 3.26% | MICE |
| Diastolic blood pressure | 186 | 0.12% | MICE |
| Alcohol intake frequency | 317 | 0.20% | MICE |
| Total cholesterol | 10,522 | 6.67% | MICE |
| HDL cholesterol | 23,052 | 14.62% | MICE |
| HbA1c | 11,366 | 7.21% | MICE |
| Serum glucose | 23,154 | 14.69% | MICE |

**Supplemental Table 3 footnote**: MET= metabolic equivalent task, MICE= multiple imputation with chained equations. Variables were imputed together, with a single replicate from 20 iterations.

**Note: Justification of missing value handling methods**

The data were filtered to complete cases across age, sex, height and weight. Next, missing values of Townsend Deprivation Index were imputed with the mean due to a very small rate of missingness, a unimodal continuous distribution and a value that cannot reasonably be estimated from other variables.

Categorical variables smoking and ethnicity, also with a very small degree of missingness, cannot be predicted from age, sex and body size, and both variables have a very large majority category (90% and 97% respectively). Therefore, missing values were assigned to the overwhelming majority group.

Finally, the remaining set of variables are continuous, somewhat related health variables, and have slightly larger rates of missingness. Therefore, multiple imputation with chained equations (MICE) was the ideal method to produce imputed values that reflect the underlying pattern of correlations in the data and allow the maximum use of all present data whilst introducing the least possible amount of bias.

## Supplemental Figure 1: Cohort matching summary


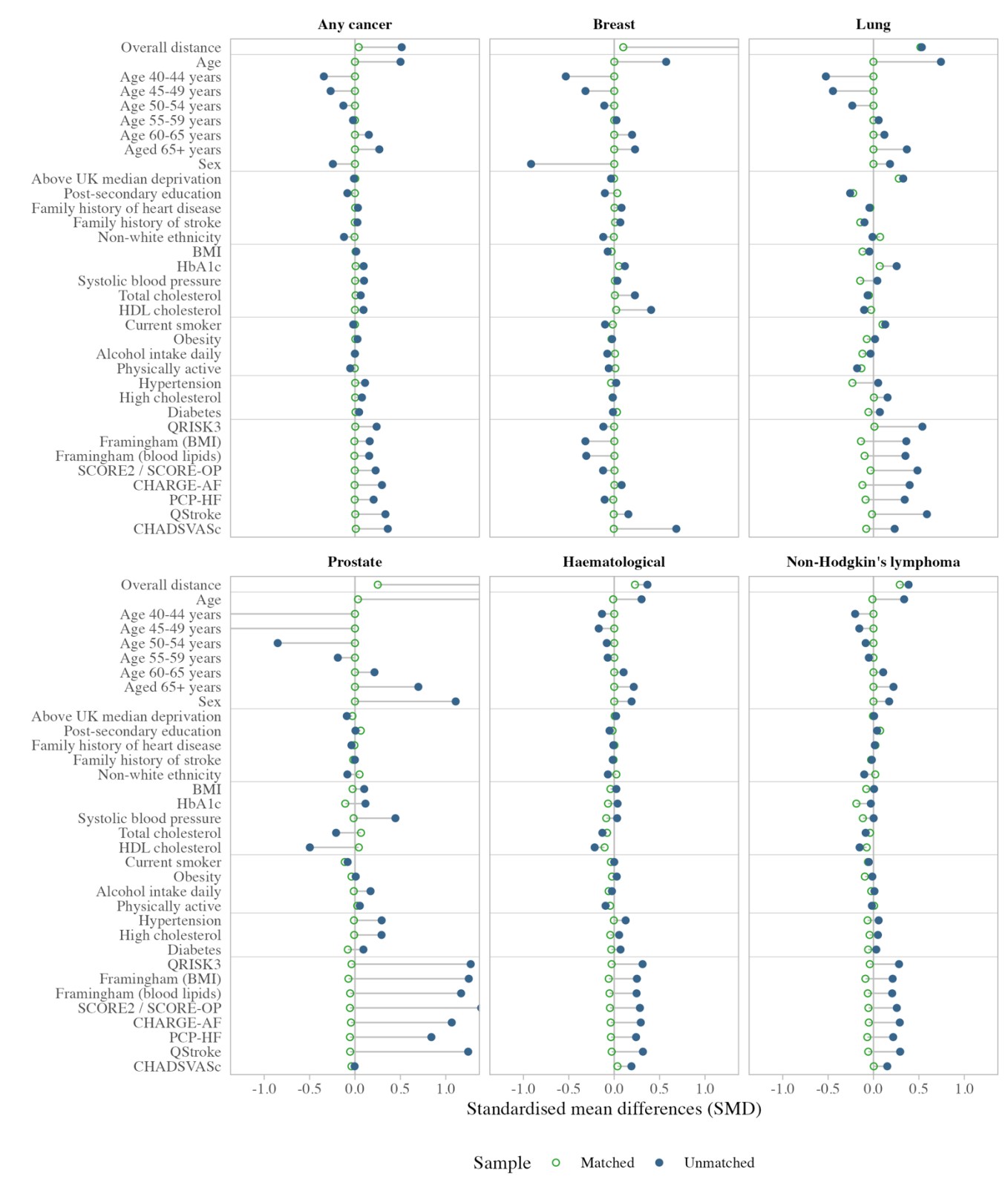


**Supplemental Figure 1 footnote**: Standardised mean differences in unmatched samples are shown in dark blue, while standardised mean differences in matched samples are shown in hollow green. The line segment in between points indicates the

agreement improvement achieved by the matching algorithm. The x-axis has been truncated to focus on the region from -1.25 to

1.25, meaning that some very large unmatched differences (in Prostate and Breast) lay outside the field of view.

## Supplemental Figure 2: Sample selection


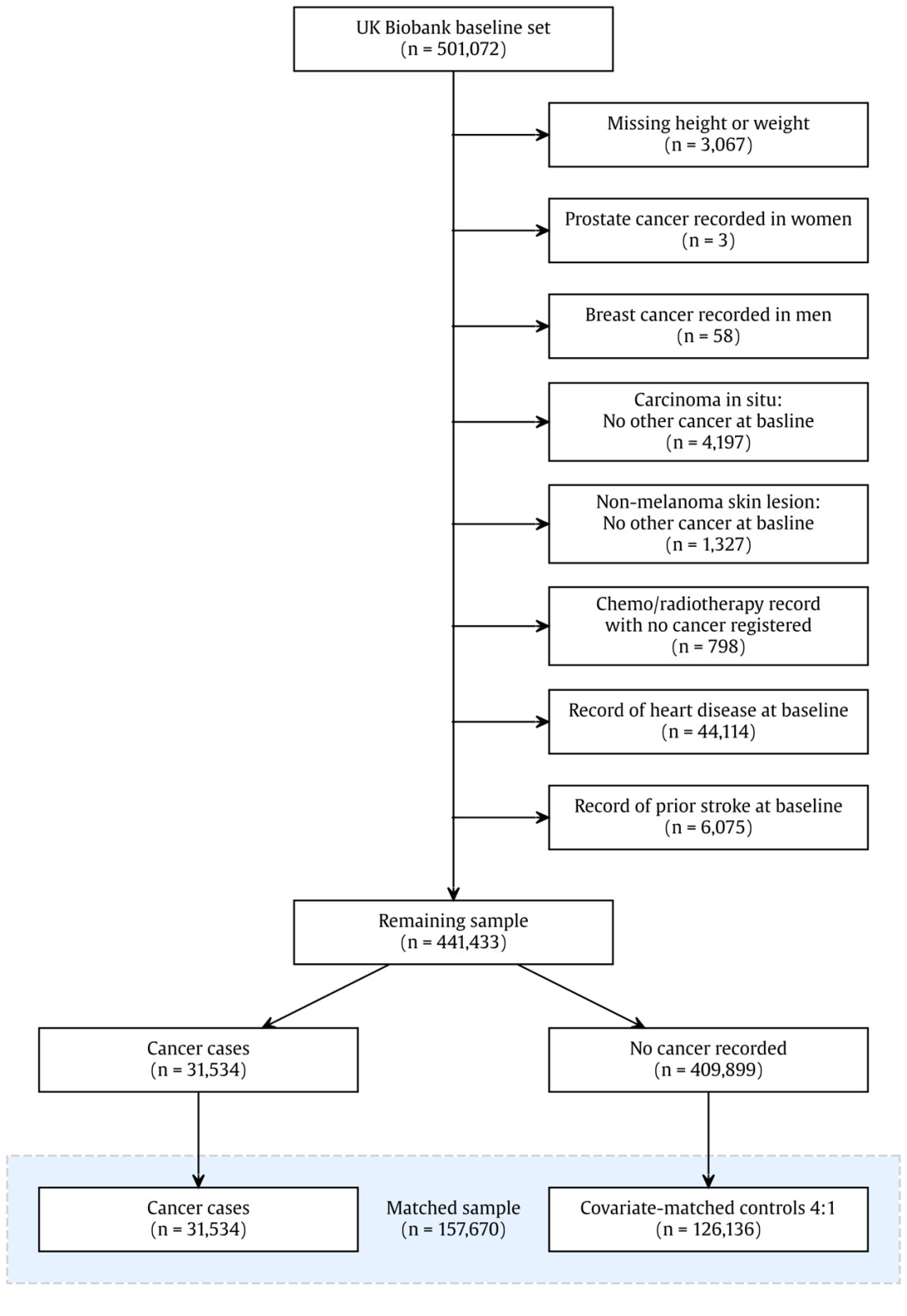


## Supplemental Table 4: Full descriptive statistics for cancer groups and their controls (solid tumors)

|  | **Any cancer** | | **Lung** | | **Breast** | | **Prostate** | | **Brain/CNS** | |
| --- | --- | --- | --- | --- | --- | --- | --- | --- | --- | --- |
| **Characteristic** | **Controls** | **Cases** | **Controls** | **Cases** | **Controls** | **Cases** | **Controls** | **Cases** | **Controls** | **Cases** |
| N | 126,136 | 31,534 | 984 | 246 | 40,668 | 10,167 | 11,380 | 2,845 | 1,016 | 254 |
| Average age (years) | 59.4 (±7.2) | 59.4 (±7.2) | 60.6 (±6.6) | 60.6 (±6.5) | 59.6 (±6.7) | 59.6 (±6.7) | 63.6 (±4.4) | 63.7 (±4.4) | 55.1 (±8.4) | 54.9 (±8.5) |
| Age: Less than 50 yrs | 15,040 (11.9%) | 3,760 (11.9%) | 76 (7.7%) | 19 (7.7%) | 3,824 (9.4%) | 956 (9.4%) | 88 (0.8%) | 22 (0.8%) | 320 (31.5%) | 80 (31.5%) |
| Age: 50-59 yrs | 37,328 (29.6%) | 9,332 (29.6%) | 296 (30.1%) | 74 (30.1%) | 12,976 (31.9%) | 3,244 (31.9%) | 1,688 (14.8%) | 422 (14.8%) | 332 (32.7%) | 83 (32.7%) |
| Age: 60-64 yrs | 37,872 (30.0%) | 9,468 (30.0%) | 280 (28.5%) | 70 (28.5%) | 13,104 (32.2%) | 3,276 (32.2%) | 3,772 (33.1%) | 943 (33.1%) | 208 (20.5%) | 52 (20.5%) |
| Age: 65 or more | 35,896 (28.5%) | 8,974 (28.5%) | 332 (33.7%) | 83 (33.7%) | 10,764 (26.5%) | 2,691 (26.5%) | 5,832 (51.2%) | 1,458 (51.2%) | 156 (15.4%) | 39 (15.4%) |
| Women | 83,852 (66.5%) | 20,963 (66.5%) | 452 (45.9%) | 113 (45.9%) | 40,668 (100.0%) | 10,167 (100.0%) |  |  | 572 (56.3%) | 143 (56.3%) |
| Men | 42,284 (33.5%) | 10,571 (33.5%) | 532 (54.1%) | 133 (54.1%) |  |  | 11,380 (100.0%) | 2,845 (100.0%) | 444 (43.7%) | 111 (43.7%) |
| Ethnicity other than White | 4,330 (3.4%) | 1,055 (3.3%) | 36 (3.7%) | 13 (5.3%) | 1,391 (3.4%) | 340 (3.3%) | 334 (2.9%) | 111 (3.9%) | 44 (4.3%) | 17 (6.7%) |
| Above UK median deprivation | 38,045 (30.2%) | 9,554 (30.3%) | 327 (33.2%) | 116 (47.2%) | 11,923 (29.3%) | 2,971 (29.2%) | 3,192 (28.0%) | 763 (26.8%) | 336 (33.1%) | 85 (33.5%) |
| Townsend Deprivation Index | -2.2 [-3.7, 0.4] | -2.2 [-3.7, 0.4] | -2.1 [-3.8, 0.8] | -0.7 [-3.1, 2.6] | -2.2 [-3.7, 0.2] | -2.3 [-3.7, 0.2] | -2.4 [-3.7, 0.1] | -2.5 [-3.8, -0.1] | -2.0 [-3.6, 0.9] | -1.9 [-3.6, 0.9] |
| Post-secondary education | 70,966 (56.3%) | 17,735 (56.2%) | 578 (58.7%) | 117 (47.6%) | 21,797 (53.6%) | 5,620 (55.3%) | 6,550 (57.6%) | 1,726 (60.7%) | 611 (60.1%) | 163 (64.2%) |
| Family history of heart disease | 55,898 (44.3%) | 14,012 (44.4%) | 417 (42.4%) | 100 (40.7%) | 18,992 (46.7%) | 4,763 (46.8%) | 4,689 (41.2%) | 1,164 (40.9%) | 395 (38.9%) | 93 (36.6%) |
| Family history of stroke | 35,596 (28.2%) | 8,872 (28.1%) | 284 (28.9%) | 56 (22.8%) | 12,023 (29.6%) | 3,050 (30.0%) | 3,156 (27.7%) | 765 (26.9%) | 267 (26.3%) | 57 (22.4%) |
| Systolic blood pressure (mmHg) | 139.5 (±19.1) | 139.6 (±19.1) | 141.6 (±18.9) | 138.5 (±20.8) | 138.2 (±19.4) | 138.3 (±19.3) | 145.7 (±17.7) | 145.5 (±17.5) | 136.6 (±18.6) | 136.9 (±18.6) |
| Total cholesterol (mmol/L) | 5.8 (±1.1) | 5.8 (±1.2) | 5.8 (±1.1) | 5.7 (±1.2) | 6.0 (±1.1) | 6.0 (±1.1) | 5.5 (±1.1) | 5.5 (±1.1) | 5.7 (±1.1) | 5.9 (±1.2) |
| HDL cholesterol (mmol/L) | 1.5 (±0.4) | 1.5 (±0.4) | 1.4 (±0.4) | 1.4 (±0.4) | 1.6 (±0.4) | 1.6 (±0.4) | 1.3 (±0.3) | 1.3 (±0.3) | 1.4 (±0.4) | 1.4 (±0.4) |
| HbA1c (mmol/mol) | 35.5 [33.2, 38.1] | 35.6 [33.2, 38.2] | 35.9 [33.5, 38.4] | 36.5 [34.1, 39.6] | 35.6 [33.3, 38.1] | 35.9 [33.5, 38.4] | 35.9 [33.5, 38.8] | 35.7 [33.3, 38.3] | 35.0 [32.3, 37.4] | 35.2 [32.0, 37.1] |
| Current smoker | 12,413 (9.8%) | 3,100 (9.8%) | 113 (11.5%) | 37 (15.0%) | 3,305 (8.1%) | 783 (7.7%) | 1,282 (11.3%) | 233 (8.2%) | 119 (11.7%) | 26 (10.2%) |
| Body mass index | 26.6 [24.0, 29.9] | 26.6 [24.0, 29.9] | 26.8 [24.2, 30.4] | 26.6 [24.0, 29.9] | 26.2 [23.5, 29.7] | 26.2 [23.6, 29.5] | 27.3 [25.0, 30.0] | 27.3 [25.0, 29.8] | 26.7 [24.0, 29.7] | 27.4 [24.2, 30.9] |
| Obesity | 30,620 (24.3%) | 7,713 (24.5%) | 267 (27.1%) | 59 (24.0%) | 9,568 (23.5%) | 2,267 (22.3%) | 2,873 (25.2%) | 672 (23.6%) | 237 (23.3%) | 80 (31.5%) |
| Alcohol intake daily | 25,661 (20.3%) | 6,416 (20.3%) | 235 (23.9%) | 47 (19.1%) | 6,976 (17.2%) | 1,778 (17.5%) | 3,261 (28.7%) | 799 (28.1%) | 181 (17.8%) | 35 (13.8%) |
| Physically active | 91,011 (72.2%) | 22,722 (72.1%) | 710 (72.2%) | 162 (65.9%) | 28,922 (71.1%) | 7,287 (71.7%) | 8,605 (75.6%) | 2,181 (76.7%) | 744 (73.2%) | 183 (72.0%) |
| Hypertension | 40,348 (32.0%) | 10,120 (32.1%) | 392 (39.8%) | 72 (29.3%) | 11,971 (29.4%) | 2,837 (27.9%) | 4,774 (42.0%) | 1,178 (41.4%) | 254 (25.0%) | 70 (27.6%) |
| High cholesterol | 22,292 (17.7%) | 5,599 (17.8%) | 206 (20.9%) | 52 (21.1%) | 6,065 (14.9%) | 1,451 (14.3%) | 3,225 (28.3%) | 795 (27.9%) | 154 (15.2%) | 42 (16.5%) |
| Diabetes | 6,686 (5.3%) | 1,718 (5.4%) | 73 (7.4%) | 15 (6.1%) | 1,441 (3.5%) | 421 (4.1%) | 994 (8.7%) | 192 (6.7%) | 56 (5.5%) | 8 (3.1%) |
| Ten-year outcomes |  |  |  |  |  |  |  |  |  |  |
| CVD 1 | 5,423 (4.3%) | 1,542 (4.9%) | 60 (6.1%) | 20 (8.1%) | 1,199 (2.9%) | 302 (3.0%) | 901 (7.9%) | 218 (7.7%) | 35 (3.4%) | 25 (9.8%) |
| CVD 2 | 12,087 (9.6%) | 3,636 (11.5%) | 132 (13.4%) | 52 (21.1%) | 2,935 (7.2%) | 847 (8.3%) | 1,974 (17.3%) | 464 (16.3%) | 68 (6.7%) | 35 (13.8%) |
| Atrial fibrillation | 5,693 (4.5%) | 1,707 (5.4%) | 62 (6.3%) | 29 (11.8%) | 1,350 (3.3%) | 397 (3.9%) | 1,007 (8.8%) | 234 (8.2%) | 23 (2.3%) | 13 (5.1%) |
| Heart failure | 2,204 (1.7%) | 789 (2.5%) | 31 (3.2%) | 17 (6.9%) | 530 (1.3%) | 188 (1.8%) | 348 (3.1%) | 97 (3.4%) | 12 (1.2%) | 2 (0.8%) |
| Stroke | 2,072 (1.6%) | 664 (2.1%) | 19 (1.9%) | 7 (2.8%) | 532 (1.3%) | 151 (1.5%) | 300 (2.6%) | 84 (3.0%) | 7 (0.7%) | 19 (7.5%) |
| Follow-up time | 13.6 [12.9, 14.4] | 13.5 [12.6, 14.3] | 13.6 [12.9, 14.3] | 12.4 [5.7, 13.9] | 13.7 [12.9, 14.4] | 13.5 [12.6, 14.3] | 13.5 [12.7, 14.3] | 13.4 [12.5, 14.2] | 13.7 [12.9, 14.3] | 13.1 [12.4, 14.0] |

**Supplemental Table 4 footnote:** *Physically active is defined as greater than 600 summed MET-minutes per week, where MET = metabolic equivalent task. Median UK Townsend Deprivation Index = -0.35 as per 2011 UK Census. CVD 1 = combined endpoint including non-fatal myocardial infarction, non-fatal stroke or cardiovascular mortality, where cardiovascular mortality is defined as any death with a primary cause from ICD10 I00-I80. CVD 2 = combined endpoint including everything from CVD 1 plus incident atrial fibrillation, heart failure, non-ischaemic cardiomyopathies and valvular heart disease. Data are presented as mean ± standard deviation for continuous variables and count (percentage) for categorical variables

## Supplemental Table 5: Full descriptive statistics for cancer groups and their controls (hematological)

|  | **Haematological** | | **Non-Hodgkin’s lymphoma** | | **Hodgkin’s lymphoma** | | **Multiple myeloma** | | **Leukaemia** | | **Polycythaemia vera** | | **Myelodysplastic syndrome** | |
| --- | --- | --- | --- | --- | --- | --- | --- | --- | --- | --- | --- | --- | --- | --- |
| **Characteristic** | **Controls** | **Cases** | **Controls** | **Cases** | **Controls** | **Cases** | **Controls** | **Cases** | **Controls** | **Cases** | **Controls** | **Cases** | **Controls** | **Cases** |
| N | 10,152 | 2,538 | 3,536 | 884 | 1,496 | 374 | 656 | 164 | 2,388 | 597 | 728 | 182 | 296 | 74 |
| Average age (years) | 58.3 (±7.9) | 58.2 (±7.9) | 58.5 (±7.7) | 58.4 (±7.7) | 54.0 (±8.2) | 53.9 (±8.1) | 60.0 (±7.1) | 59.9 (±7.1) | 59.2 (±7.7) | 59.1 (±7.8) | 58.8 (±7.4) | 58.5 (±7.4) | 60.1 (±7.6) | 60.3 (±7.5) |
| Age: Less than 50 yrs | 1,768 (17.4%) | 442 (17.4%) | 580 (16.4%) | 145 (16.4%) | 504 (33.7%) | 126 (33.7%) | 76 (11.6%) | 19 (11.6%) | 364 (15.2%) | 91 (15.2%) | 92 (12.6%) | 23 (12.6%) | 32 (10.8%) | 8 (10.8%) |
| Age: 50-59 yrs | 2,960 (29.2%) | 740 (29.2%) | 1,052 (29.8%) | 263 (29.8%) | 552 (36.9%) | 138 (36.9%) | 160 (24.4%) | 40 (24.4%) | 612 (25.6%) | 153 (25.6%) | 236 (32.4%) | 59 (32.4%) | 64 (21.6%) | 16 (21.6%) |
| Age: 60-64 yrs | 2,808 (27.7%) | 702 (27.7%) | 984 (27.8%) | 246 (27.8%) | 256 (17.1%) | 64 (17.1%) | 220 (33.5%) | 55 (33.5%) | 704 (29.5%) | 176 (29.5%) | 220 (30.2%) | 55 (30.2%) | 104 (35.1%) | 26 (35.1%) |
| Age: 65 or more | 2,616 (25.8%) | 654 (25.8%) | 920 (26.0%) | 230 (26.0%) | 184 (12.3%) | 46 (12.3%) | 200 (30.5%) | 50 (30.5%) | 708 (29.6%) | 177 (29.6%) | 180 (24.7%) | 45 (24.7%) | 96 (32.4%) | 24 (32.4%) |
| Women | 4,616 (45.5%) | 1,154 (45.5%) | 1,640 (46.4%) | 410 (46.4%) | 768 (51.3%) | 192 (51.3%) | 284 (43.3%) | 71 (43.3%) | 1,080 (45.2%) | 270 (45.2%) | 200 (27.5%) | 50 (27.5%) | 148 (50.0%) | 37 (50.0%) |
| Men | 5,536 (54.5%) | 1,384 (54.5%) | 1,896 (53.6%) | 474 (53.6%) | 728 (48.7%) | 182 (48.7%) | 372 (56.7%) | 93 (56.7%) | 1,308 (54.8%) | 327 (54.8%) | 528 (72.5%) | 132 (72.5%) | 148 (50.0%) | 37 (50.0%) |
| Ethnicity other than White | 372 (3.7%) | 105 (4.1%) | 115 (3.3%) | 32 (3.6%) | 74 (4.9%) | 16 (4.3%) | 20 (3.0%) | 9 (5.5%) | 89 (3.7%) | 16 (2.7%) | 24 (3.3%) | 3 (1.6%) | 8 (2.7%) | 5 (6.8%) |
| Above UK median deprivation | 3,202 (31.5%) | 806 (31.8%) | 1,109 (31.4%) | 275 (31.1%) | 468 (31.3%) | 117 (31.3%) | 215 (32.8%) | 47 (28.7%) | 762 (31.9%) | 187 (31.3%) | 236 (32.4%) | 74 (40.7%) | 95 (32.1%) | 27 (36.5%) |
| Townsend Deprivation Index | -2.1 [-3.7, 0.6] | -2.1 [-3.7, 0.6] | -2.2 [-3.7, 0.6] | -2.0 [-3.7, 0.7] | -2.0 [-3.6, 0.6] | -2.0 [-3.7, 0.5] | -2.1 [-3.7, 0.6] | -2.4 [-3.6, 0.1] | -2.1 [-3.7, 0.6] | -2.3 [-3.7, 0.4] | -2.2 [-3.7, 0.6] | -1.2 [-3.4, 1.5] | -2.2 [-3.7, 0.5] | -1.8 [-3.4, 1.1] |
| Post-secondary education | 5,962 (58.7%) | 1,468 (57.8%) | 2,079 (58.8%) | 551 (62.3%) | 969 (64.8%) | 232 (62.0%) | 354 (54.0%) | 95 (57.9%) | 1,386 (58.0%) | 322 (53.9%) | 422 (58.0%) | 91 (50.0%) | 180 (60.8%) | 40 (54.1%) |
| Family history of heart disease | 4,290 (42.3%) | 1,075 (42.4%) | 1,503 (42.5%) | 385 (43.6%) | 588 (39.3%) | 137 (36.6%) | 293 (44.7%) | 69 (42.1%) | 1,033 (43.3%) | 262 (43.9%) | 312 (42.9%) | 78 (42.9%) | 119 (40.2%) | 31 (41.9%) |
| Family history of stroke | 2,694 (26.5%) | 665 (26.2%) | 964 (27.3%) | 231 (26.1%) | 355 (23.7%) | 76 (20.3%) | 173 (26.4%) | 60 (36.6%) | 642 (26.9%) | 151 (25.3%) | 184 (25.3%) | 56 (30.8%) | 89 (30.1%) | 18 (24.3%) |
| Systolic blood pressure (mmHg) | 139.9 (±18.7) | 138.3 (±19.0) | 139.9 (±18.8) | 137.7 (±18.5) | 136.1 (±18.0) | 135.9 (±20.5) | 141.0 (±18.4) | 138.1 (±18.0) | 141.2 (±19.0) | 139.1 (±19.1) | 141.2 (±18.2) | 141.1 (±18.0) | 142.9 (±18.8) | 140.8 (±19.2) |
| Total cholesterol (mmol/L) | 5.7 (±1.1) | 5.6 (±1.2) | 5.7 (±1.1) | 5.7 (±1.2) | 5.7 (±1.0) | 5.8 (±1.1) | 5.7 (±1.2) | 5.5 (±1.2) | 5.7 (±1.2) | 5.6 (±1.2) | 5.5 (±1.1) | 5.1 (±1.3) | 5.7 (±1.2) | 5.3 (±1.1) |
| HDL cholesterol (mmol/L) | 1.4 (±0.4) | 1.4 (±0.4) | 1.4 (±0.4) | 1.4 (±0.4) | 1.4 (±0.4) | 1.5 (±0.4) | 1.4 (±0.4) | 1.4 (±0.4) | 1.4 (±0.4) | 1.3 (±0.4) | 1.3 (±0.4) | 1.3 (±0.4) | 1.4 (±0.4) | 1.4 (±0.5) |
| HbA1c (mmol/mol) | 35.4 [33.0, 38.1] | 35.3 [32.5, 38.1] | 35.5 [33.2, 38.2] | 35.0 [32.5, 37.7] | 34.7 [32.4, 37.4] | 35.2 [32.6, 38.0] | 35.9 [33.2, 38.7] | 36.5 [33.4, 39.2] | 35.5 [33.1, 38.1] | 35.7 [33.1, 38.5] | 36.0 [33.3, 38.2] | 35.2 [31.6, 37.9] | 35.2 [33.1, 38.4] | 34.6 [30.9, 37.1] |
| Current smoker | 1,169 (11.5%) | 264 (10.4%) | 377 (10.7%) | 79 (8.9%) | 181 (12.1%) | 45 (12.0%) | 79 (12.0%) | 13 (7.9%) | 262 (11.0%) | 47 (7.9%) | 110 (15.1%) | 37 (20.3%) | 29 (9.8%) | 8 (10.8%) |
| Body mass index | 26.9 [24.4, 30.1] | 26.6 [24.1, 29.9] | 27.0 [24.3, 30.2] | 26.6 [23.9, 29.4] | 26.5 [24.0, 29.8] | 25.8 [23.5, 29.3] | 27.0 [24.5, 30.5] | 26.8 [24.6, 30.5] | 26.9 [24.4, 29.9] | 26.9 [24.5, 30.2] | 26.8 [24.7, 30.1] | 28.1 [24.6, 31.6] | 27.4 [24.7, 30.6] | 26.9 [24.0, 28.4] |
| Obesity | 2,590 (25.5%) | 621 (24.5%) | 945 (26.7%) | 201 (22.7%) | 348 (23.3%) | 79 (21.1%) | 180 (27.4%) | 44 (26.8%) | 587 (24.6%) | 155 (26.0%) | 188 (25.8%) | 65 (35.7%) | 83 (28.0%) | 10 (13.5%) |
| Alcohol intake daily | 2,215 (21.8%) | 492 (19.4%) | 771 (21.8%) | 184 (20.8%) | 288 (19.3%) | 65 (17.4%) | 136 (20.7%) | 24 (14.6%) | 547 (22.9%) | 117 (19.6%) | 170 (23.4%) | 48 (26.4%) | 65 (22.0%) | 10 (13.5%) |
| Physically active | 7,319 (72.1%) | 1,777 (70.0%) | 2,594 (73.4%) | 651 (73.6%) | 1,101 (73.6%) | 261 (69.8%) | 460 (70.1%) | 98 (59.8%) | 1,706 (71.4%) | 410 (68.7%) | 516 (70.9%) | 129 (70.9%) | 212 (71.6%) | 46 (62.2%) |
| Hypertension | 3,353 (33.0%) | 833 (32.8%) | 1,149 (32.5%) | 261 (29.5%) | 395 (26.4%) | 84 (22.5%) | 228 (34.8%) | 65 (39.6%) | 839 (35.1%) | 185 (31.0%) | 274 (37.6%) | 110 (60.4%) | 108 (36.5%) | 28 (37.8%) |
| High cholesterol | 1,873 (18.4%) | 427 (16.8%) | 644 (18.2%) | 147 (16.6%) | 183 (12.2%) | 52 (13.9%) | 140 (21.3%) | 22 (13.4%) | 472 (19.8%) | 106 (17.8%) | 160 (22.0%) | 46 (25.3%) | 52 (17.6%) | 13 (17.6%) |
| Diabetes | 695 (6.8%) | 154 (6.1%) | 226 (6.4%) | 45 (5.1%) | 66 (4.4%) | 15 (4.0%) | 55 (8.4%) | 13 (7.9%) | 167 (7.0%) | 36 (6.0%) | 71 (9.8%) | 20 (11.0%) | 21 (7.1%) | 6 (8.1%) |
| Ten-year outcomes |  |  |  |  |  |  |  |  |  |  |  |  |  |  |
| CVD 1 | 507 (5.0%) | 227 (8.9%) | 185 (5.2%) | 55 (6.2%) | 68 (4.5%) | 45 (12.0%) | 39 (5.9%) | 18 (11.0%) | 111 (4.6%) | 53 (8.9%) | 41 (5.6%) | 20 (11.0%) | 8 (2.7%) | 11 (14.9%) |
| CVD 2 | 1,058 (10.4%) | 533 (21.0%) | 378 (10.7%) | 145 (16.4%) | 109 (7.3%) | 89 (23.8%) | 77 (11.7%) | 36 (22.0%) | 252 (10.6%) | 134 (22.4%) | 80 (11.0%) | 43 (23.6%) | 33 (11.1%) | 18 (24.3%) |
| Atrial fibrillation | 500 (4.9%) | 230 (9.1%) | 186 (5.3%) | 65 (7.4%) | 43 (2.9%) | 29 (7.8%) | 32 (4.9%) | 16 (9.8%) | 124 (5.2%) | 60 (10.1%) | 36 (4.9%) | 18 (9.9%) | 20 (6.8%) | 8 (10.8%) |
| Heart failure | 217 (2.1%) | 165 (6.5%) | 70 (2.0%) | 50 (5.7%) | 20 (1.3%) | 25 (6.7%) | 21 (3.2%) | 12 (7.3%) | 49 (2.1%) | 32 (5.4%) | 16 (2.2%) | 16 (8.8%) | 9 (3.0%) | 7 (9.5%) |
| Stroke | 180 (1.8%) | 86 (3.4%) | 69 (2.0%) | 20 (2.3%) | 24 (1.6%) | 12 (3.2%) | 12 (1.8%) | 8 (4.9%) | 38 (1.6%) | 21 (3.5%) | 13 (1.8%) | 8 (4.4%) | 3 (1.0%) | 7 (9.5%) |
| Follow-up time | 13.6 [12.9, 14.3] | 13.2 [12.3, 14.1] | 13.6 [12.9, 14.3] | 13.3 [12.4, 14.1] | 13.8 [13.0, 14.5] | 13.4 [12.5, 14.3] | 13.5 [12.7, 14.3] | 11.8 [5.8, 13.6] | 13.6 [12.8, 14.3] | 13.4 [12.4, 14.2] | 13.6 [12.7, 14.4] | 13.3 [12.4, 14.1] | 13.6 [12.9, 14.3] | 12.8 [8.5, 13.6] |

**Supplemental Table 5 footnote:** *Physically active is defined as greater than 600 summed MET-minutes per week, where MET = metabolic equivalent task. Median UK Townsend Deprivation Index = -0.35 as per 2011 UK Census. CVD 1 = combined endpoint including non-fatal myocardial infarction, non-fatal stroke or cardiovascular mortality, where cardiovascular mortality is defined as any death with a primary cause from ICD10 I00-I80. CVD 2 = combined endpoint including everything from CVD 1 plus incident atrial fibrillation, heart failure, non-ischaemic cardiomyopathies and valvular heart disease. Data are presented as mean ± standard deviation for continuous variables and count (percentage) for categorical variables

13

## Supplemental Figure 3: Risk scores by cancer group


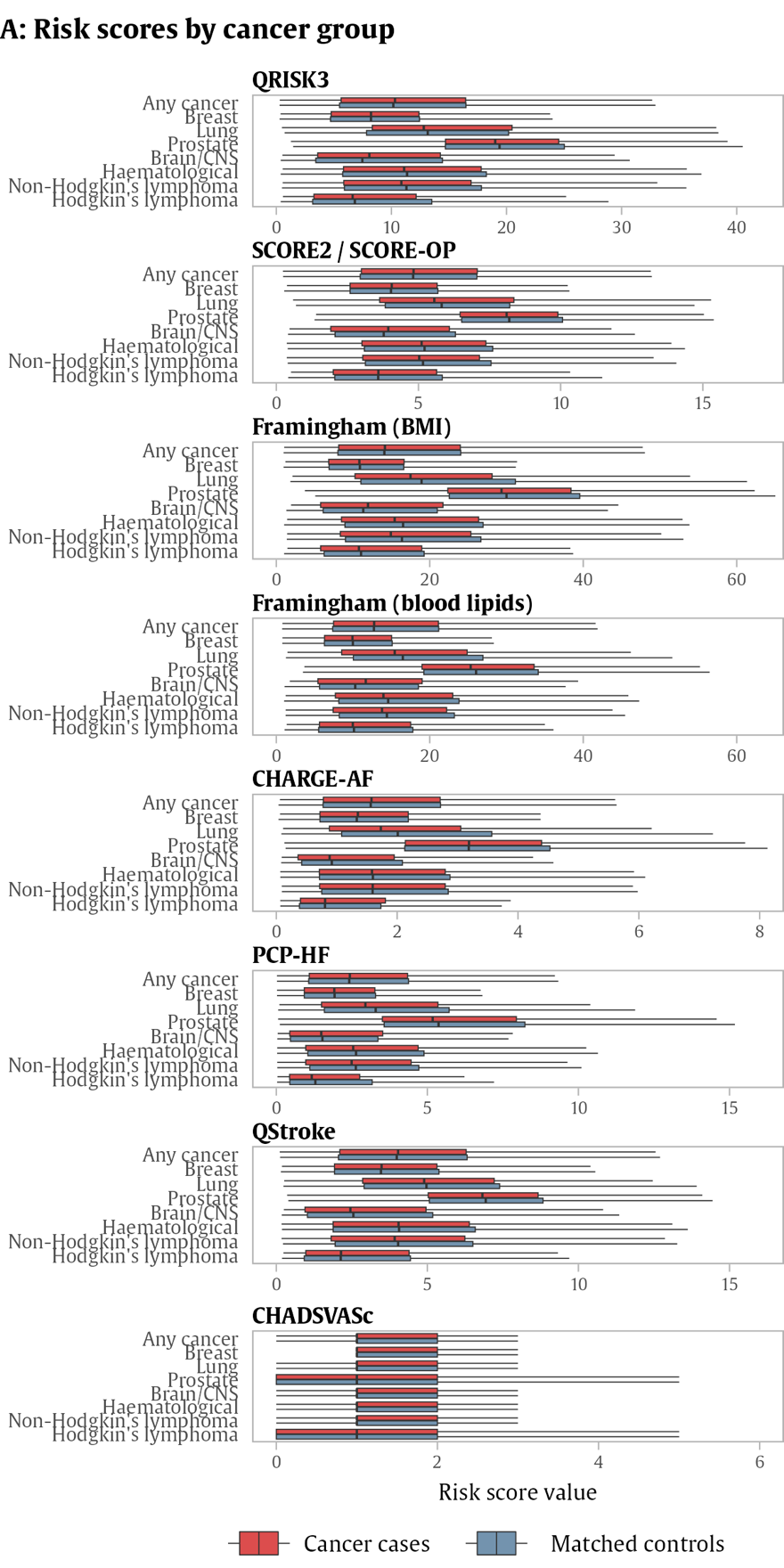


**Supplemental Figure 3 footnote**: Boxplots show median risk score for cancer cases (in red) and non-cancer matched controls (in blue), with coloured rectangles indicating the interval from 25^th^ percentile to 75^th^ percentile.

## Supplemental Table 6: Risk score statistical summaries

| Risk score | Cancer | Group | Count | Mean | SD | Min | 25% | 50% | 75% | Max |
| --- | --- | --- | --- | --- | --- | --- | --- | --- | --- | --- |
| QRISK3 | Any cancer | Cancer cases | 31,534 | 12.04 | 8.54 | 0.30 | 5.63 | 10.32 | 16.45 | 85.85 |
|  | Any cancer | Controls | 126,136 | 12.01 | 8.69 | 0.29 | 5.49 | 10.17 | 16.48 | 83.53 |
|  | Breast | Cancer cases | 10,167 | 9.27 | 6.18 | 0.37 | 4.77 | 8.23 | 12.39 | 85.85 |
|  | Breast | Controls | 40,668 | 9.28 | 6.17 | 0.29 | 4.72 | 8.23 | 12.44 | 63.92 |
|  | Lung | Cancer cases | 246 | 14.98 | 9.22 | 0.49 | 8.34 | 12.82 | 20.48 | 52.75 |
|  | Lung | Controls | 984 | 14.88 | 9.42 | 0.73 | 7.84 | 13.16 | 20.18 | 63.38 |
|  | Prostate | Cancer cases | 2,845 | 20.21 | 8.00 | 1.28 | 14.71 | 19.02 | 24.54 | 69.45 |
|  | Prostate | Controls | 11,380 | 20.51 | 8.51 | 1.47 | 14.69 | 19.40 | 25.02 | 75.29 |
|  | Brain/CNS | Cancer cases | 254 | 9.95 | 8.18 | 0.53 | 3.60 | 8.09 | 14.25 | 41.50 |
|  | Brain/CNS | Controls | 1,016 | 9.86 | 8.32 | 0.39 | 3.44 | 7.48 | 14.44 | 61.05 |
|  | Haematological | Cancer cases | 2,538 | 12.92 | 9.32 | 0.55 | 5.84 | 11.11 | 17.80 | 66.11 |
|  | Haematological | Controls | 10,152 | 13.19 | 9.71 | 0.38 | 5.77 | 11.37 | 18.25 | 78.44 |
|  | Non-Hodgkin's lymphoma | Cancer cases | 884 | 12.50 | 8.84 | 0.55 | 5.87 | 10.87 | 16.93 | 62.46 |
|  | Non-Hodgkin's lymphoma | Controls | 3,536 | 12.85 | 8.96 | 0.49 | 5.92 | 11.31 | 17.82 | 63.68 |
|  | Hodgkin’s lymphoma | Cancer cases | 374 | 9.11 | 8.28 | 0.58 | 3.27 | 6.63 | 12.15 | 57.66 |
|  | Hodgkin’s lymphoma | Controls | 1,496 | 9.35 | 8.40 | 0.38 | 3.16 | 6.83 | 13.51 | 78.11 |
|  | Multiple myeloma | Cancer cases | 164 | 14.86 | 10.27 | 1.09 | 7.91 | 11.76 | 19.92 | 55.60 |
|  | Multiple myeloma | Controls | 656 | 15.27 | 10.69 | 0.90 | 7.35 | 12.82 | 20.75 | 66.71 |
|  | Leukaemia | Cancer cases | 597 | 13.73 | 9.65 | 0.62 | 6.61 | 12.13 | 18.73 | 66.11 |
|  | Leukaemia | Controls | 2,388 | 14.12 | 10.19 | 0.45 | 6.61 | 12.17 | 19.14 | 75.66 |
|  | Other haem | Cancer cases | 263 | 14.41 | 9.67 | 0.67 | 7.13 | 13.36 | 19.38 | 54.55 |
|  | Other haem | Controls | 1,052 | 14.43 | 9.96 | 0.50 | 7.12 | 12.83 | 19.51 | 78.44 |
| Framingham (BMI) | Any cancer | Cancer cases | 31,534 | 17.74 | 12.89 | 1.03 | 8.14 | 14.17 | 23.98 | 95.10 |
|  | Any cancer | Controls | 126,136 | 17.81 | 13.22 | 0.96 | 8.04 | 14.09 | 24.04 | 95.57 |
|  | Breast | Cancer cases | 10,167 | 12.91 | 8.62 | 1.18 | 6.84 | 10.83 | 16.66 | 87.31 |
|  | Breast | Controls | 40,668 | 12.90 | 8.54 | 0.96 | 6.88 | 10.90 | 16.62 | 92.46 |
|  | Lung | Cancer cases | 246 | 20.66 | 13.86 | 2.11 | 10.27 | 17.49 | 28.13 | 81.12 |
|  | Lung | Controls | 984 | 22.57 | 15.06 | 1.84 | 11.03 | 18.94 | 31.16 | 83.27 |
|  | Prostate | Cancer cases | 2,845 | 31.37 | 12.55 | 3.73 | 22.35 | 29.35 | 38.40 | 88.19 |
|  | Prostate | Controls | 11,380 | 32.28 | 13.43 | 5.08 | 22.55 | 30.00 | 39.55 | 92.97 |
|  | Brain/CNS | Cancer cases | 254 | 15.12 | 11.61 | 1.93 | 5.78 | 11.96 | 21.73 | 64.83 |
|  | Brain/CNS | Controls | 1,016 | 15.34 | 12.49 | 1.32 | 6.11 | 11.34 | 20.99 | 68.01 |
|  | Haematological | Cancer cases | 2,538 | 19.06 | 13.68 | 1.41 | 8.50 | 15.43 | 26.37 | 95.10 |
|  | Haematological | Controls | 10,152 | 19.89 | 14.30 | 1.02 | 8.96 | 16.53 | 26.95 | 93.23 |
|  | Non-Hodgkin's lymphoma | Cancer cases | 884 | 18.42 | 13.14 | 1.41 | 8.35 | 14.93 | 25.36 | 70.66 |
|  | Non-Hodgkin's lymphoma | Controls | 3,536 | 19.59 | 13.69 | 1.37 | 9.02 | 16.38 | 26.67 | 87.47 |
|  | Hodgkins lymphoma | Cancer cases | 374 | 14.38 | 12.08 | 1.45 | 5.78 | 10.77 | 18.96 | 72.78 |
|  | Hodgkins lymphoma | Controls | 1,496 | 14.62 | 11.92 | 1.02 | 6.24 | 11.05 | 19.27 | 87.47 |
|  | Multiple myeloma | Cancer cases | 164 | 20.70 | 13.82 | 2.47 | 9.93 | 18.01 | 26.73 | 69.38 |
|  | Multiple myeloma | Controls | 656 | 22.03 | 14.70 | 2.55 | 10.57 | 18.96 | 29.60 | 79.05 |
|  | Leukemia | Cancer cases | 597 | 19.41 | 13.20 | 1.43 | 8.87 | 16.30 | 27.66 | 88.41 |
|  | Leukemia | Controls | 2,388 | 21.05 | 14.86 | 1.51 | 9.49 | 17.61 | 28.70 | 93.23 |
|  | Other heme | Cancer cases | 263 | 21.11 | 15.10 | 1.91 | 10.23 | 17.81 | 28.45 | 95.10 |
|  | Other heme | Controls | 1,052 | 21.44 | 15.11 | 1.27 | 10.17 | 18.42 | 28.30 | 92.91 |
| Framingham (blood lipids) | Any cancer | Cancer cases | 31,534 | 15.70 | 11.19 | 0.80 | 7.47 | 12.75 | 21.13 | 97.01 |
|  | Any cancer | Controls | 126,136 | 15.77 | 11.46 | 0.76 | 7.35 | 12.73 | 21.16 | 96.98 |
|  | Breast | Cancer cases | 10,167 | 11.64 | 7.51 | 0.80 | 6.29 | 9.93 | 15.03 | 97.01 |
|  | Breast | Controls | 40,668 | 11.62 | 7.40 | 0.78 | 6.27 | 9.99 | 15.11 | 69.61 |
|  | Lung | Cancer cases | 246 | 18.41 | 12.68 | 1.46 | 8.52 | 15.44 | 24.87 | 70.06 |
|  | Lung | Controls | 984 | 19.65 | 12.77 | 1.26 | 10.05 | 16.49 | 26.93 | 76.32 |
|  | Prostate | Cancer cases | 2,845 | 27.27 | 11.39 | 3.66 | 19.00 | 25.34 | 33.60 | 77.55 |
|  | Prostate | Controls | 11,380 | 27.87 | 12.04 | 3.47 | 19.23 | 26.04 | 34.14 | 86.36 |
|  | Brain/CNS | Cancer cases | 254 | 13.60 | 10.21 | 1.76 | 5.43 | 11.66 | 19.02 | 66.72 |
|  | Brain/CNS | Controls | 1,016 | 13.66 | 11.00 | 1.08 | 5.64 | 10.29 | 18.52 | 80.13 |
|  | Hematological | Cancer cases | 2,538 | 16.91 | 12.03 | 1.22 | 7.71 | 13.97 | 23.00 | 87.36 |
|  | Hematological | Controls | 10,152 | 17.53 | 12.50 | 1.02 | 8.15 | 14.58 | 23.81 | 96.98 |
|  | Non-Hodgkin's lymphoma | Cancer cases | 884 | 16.35 | 11.66 | 1.22 | 7.42 | 13.78 | 22.20 | 76.39 |
|  | Non-Hodgkin's lymphoma | Controls | 3,536 | 17.08 | 11.69 | 1.21 | 8.21 | 14.41 | 23.21 | 84.77 |
|  | Hodgkins lymphoma | Cancer cases | 374 | 13.28 | 11.14 | 1.36 | 5.63 | 9.97 | 17.51 | 65.50 |
|  | Hodgkins lymphoma | Controls | 1,496 | 13.03 | 10.32 | 1.08 | 5.52 | 10.13 | 17.80 | 84.32 |
|  | Multiple myeloma | Cancer cases | 164 | 18.36 | 12.54 | 1.88 | 9.11 | 15.01 | 24.98 | 57.68 |
|  | Multiple myeloma | Controls | 656 | 19.71 | 13.25 | 1.82 | 9.21 | 16.73 | 26.79 | 84.54 |
|  | Leukemia | Cancer cases | 597 | 17.65 | 11.88 | 1.62 | 8.38 | 14.58 | 24.80 | 87.36 |
|  | Leukemia | Controls | 2,388 | 18.70 | 13.24 | 1.15 | 8.73 | 15.52 | 25.02 | 91.97 |
|  | Other heme | Cancer cases | 263 | 18.60 | 13.24 | 1.25 | 8.69 | 16.05 | 25.17 | 86.53 |
|  | Other heme | Controls | 1,052 | 18.93 | 13.28 | 1.02 | 9.21 | 15.92 | 25.73 | 96.98 |
| SCORE2 / SCORE-OP | Any cancer | Cancer cases | 31,534 | 5.28 | 3.02 | 0.23 | 2.99 | 4.83 | 7.07 | 25.38 |
|  | Any cancer | Controls | 126,136 | 5.28 | 3.08 | 0.24 | 2.95 | 4.81 | 7.05 | 29.08 |
|  | Breast | Cancer cases | 10,167 | 4.31 | 2.28 | 0.38 | 2.60 | 4.06 | 5.66 | 25.38 |
|  | Breast | Controls | 40,668 | 4.31 | 2.26 | 0.28 | 2.60 | 4.03 | 5.68 | 22.80 |
|  | Lung | Cancer cases | 246 | 6.17 | 3.25 | 0.59 | 3.64 | 5.56 | 8.36 | 18.31 |
|  | Lung | Controls | 984 | 6.27 | 3.30 | 0.69 | 3.83 | 5.82 | 8.21 | 22.89 |
|  | Prostate | Cancer cases | 2,845 | 8.33 | 2.69 | 1.40 | 6.46 | 8.10 | 9.90 | 20.22 |
|  | Prostate | Controls | 11,380 | 8.48 | 2.88 | 1.35 | 6.52 | 8.19 | 10.07 | 26.17 |
|  | Brain/CNS | Cancer cases | 254 | 4.41 | 2.90 | 0.47 | 1.92 | 3.94 | 6.09 | 16.21 |
|  | Brain/CNS | Controls | 1,016 | 4.50 | 3.09 | 0.41 | 2.07 | 3.78 | 6.30 | 21.03 |
|  | Hematological | Cancer cases | 2,538 | 5.49 | 3.17 | 0.37 | 3.01 | 5.11 | 7.37 | 23.30 |
|  | Hematological | Controls | 10,152 | 5.64 | 3.29 | 0.40 | 3.10 | 5.21 | 7.61 | 28.53 |
|  | Non-Hodgkin's lymphoma | Cancer cases | 884 | 5.39 | 3.12 | 0.37 | 3.04 | 5.03 | 7.14 | 21.92 |
|  | Non-Hodgkin's lymphoma | Controls | 3,536 | 5.57 | 3.14 | 0.40 | 3.13 | 5.16 | 7.55 | 21.98 |
|  | Hodgkins lymphoma | Cancer cases | 374 | 4.27 | 2.97 | 0.52 | 2.01 | 3.59 | 5.64 | 17.35 |
|  | Hodgkins lymphoma | Controls | 1,496 | 4.23 | 2.84 | 0.42 | 2.06 | 3.58 | 5.84 | 21.41 |
|  | Multiple myeloma | Cancer cases | 164 | 5.87 | 3.07 | 1.01 | 3.50 | 5.51 | 7.50 | 16.72 |
|  | Multiple myeloma | Controls | 656 | 6.27 | 3.29 | 0.61 | 3.78 | 5.91 | 8.25 | 20.76 |
|  | Leukemia | Cancer cases | 597 | 5.76 | 3.16 | 0.55 | 3.26 | 5.36 | 7.84 | 22.70 |
|  | Leukemia | Controls | 2,388 | 5.97 | 3.39 | 0.43 | 3.35 | 5.55 | 7.99 | 24.23 |
|  | Other heme | Cancer cases | 263 | 6.05 | 3.35 | 0.63 | 3.79 | 5.70 | 7.95 | 23.30 |
|  | Other heme | Controls | 1,052 | 6.09 | 3.40 | 0.44 | 3.62 | 5.71 | 7.94 | 28.53 |
| CHARGE-AF | Any cancer | Cancer cases | 31,534 | 1.98 | 1.61 | 0.06 | 0.78 | 1.58 | 2.71 | 19.63 |
|  | Any cancer | Controls | 126,136 | 1.99 | 1.65 | 0.04 | 0.77 | 1.56 | 2.72 | 21.98 |
|  | Breast | Cancer cases | 10,167 | 1.60 | 1.15 | 0.06 | 0.72 | 1.35 | 2.18 | 12.75 |
|  | Breast | Controls | 40,668 | 1.60 | 1.15 | 0.04 | 0.72 | 1.33 | 2.18 | 12.03 |
|  | Lung | Cancer cases | 246 | 2.28 | 1.95 | 0.11 | 0.88 | 1.73 | 3.05 | 12.85 |
|  | Lung | Controls | 984 | 2.52 | 1.97 | 0.08 | 1.08 | 2.01 | 3.57 | 15.73 |
|  | Prostate | Cancer cases | 2,845 | 3.46 | 1.84 | 0.13 | 2.14 | 3.19 | 4.39 | 19.63 |
|  | Prostate | Controls | 11,380 | 3.54 | 1.95 | 0.15 | 2.13 | 3.19 | 4.53 | 16.10 |
|  | Brain/CNS | Cancer cases | 254 | 1.40 | 1.41 | 0.09 | 0.36 | 0.88 | 1.95 | 10.37 |
|  | Brain/CNS | Controls | 1,016 | 1.47 | 1.44 | 0.08 | 0.42 | 0.92 | 2.09 | 10.42 |
|  | Hematological | Cancer cases | 2,538 | 2.00 | 1.70 | 0.07 | 0.71 | 1.58 | 2.80 | 15.33 |
|  | Hematological | Controls | 10,152 | 2.07 | 1.78 | 0.07 | 0.72 | 1.60 | 2.87 | 18.10 |
|  | Non-Hodgkin's lymphoma | Cancer cases | 884 | 1.95 | 1.55 | 0.09 | 0.72 | 1.60 | 2.80 | 8.86 |
|  | Non-Hodgkin's lymphoma | Controls | 3,536 | 2.03 | 1.67 | 0.09 | 0.75 | 1.59 | 2.85 | 14.65 |
|  | Hodgkins lymphoma | Cancer cases | 374 | 1.36 | 1.55 | 0.07 | 0.40 | 0.81 | 1.81 | 15.33 |
|  | Hodgkins lymphoma | Controls | 1,496 | 1.34 | 1.47 | 0.07 | 0.38 | 0.81 | 1.73 | 12.81 |
|  | Multiple myeloma | Cancer cases | 164 | 2.19 | 1.81 | 0.18 | 0.89 | 1.79 | 2.78 | 10.19 |
|  | Multiple myeloma | Controls | 656 | 2.43 | 2.02 | 0.12 | 1.02 | 1.88 | 3.29 | 18.10 |
|  | Leukemia | Cancer cases | 597 | 2.12 | 1.68 | 0.11 | 0.81 | 1.75 | 2.97 | 11.66 |
|  | Leukemia | Controls | 2,388 | 2.25 | 1.87 | 0.08 | 0.83 | 1.82 | 3.10 | 13.10 |
|  | Other heme | Cancer cases | 263 | 2.26 | 1.83 | 0.13 | 0.87 | 1.90 | 3.09 | 13.59 |
|  | Other heme | Controls | 1,052 | 2.29 | 1.82 | 0.11 | 0.88 | 1.92 | 3.22 | 12.89 |
| PCP-HF | Any cancer | Cancer cases | 31,534 | 3.35 | 3.52 | 0.02 | 1.09 | 2.43 | 4.35 | 57.59 |
|  | Any cancer | Controls | 126,136 | 3.36 | 3.55 | 0.02 | 1.07 | 2.42 | 4.38 | 59.11 |
|  | Breast | Cancer cases | 10,167 | 2.39 | 2.20 | 0.02 | 0.93 | 1.92 | 3.26 | 40.19 |
|  | Breast | Controls | 40,668 | 2.42 | 2.21 | 0.03 | 0.93 | 1.93 | 3.29 | 41.17 |
|  | Lung | Cancer cases | 246 | 3.96 | 3.87 | 0.10 | 1.50 | 2.95 | 5.35 | 36.02 |
|  | Lung | Controls | 984 | 4.30 | 3.80 | 0.06 | 1.59 | 3.29 | 5.72 | 30.05 |
|  | Prostate | Cancer cases | 2,845 | 6.25 | 4.31 | 0.06 | 3.51 | 5.18 | 7.95 | 57.59 |
|  | Prostate | Controls | 11,380 | 6.49 | 4.61 | 0.12 | 3.57 | 5.37 | 8.23 | 59.11 |
|  | Brain/CNS | Cancer cases | 254 | 2.55 | 3.23 | 0.05 | 0.45 | 1.49 | 3.52 | 31.03 |
|  | Brain/CNS | Controls | 1,016 | 2.52 | 3.01 | 0.03 | 0.47 | 1.53 | 3.37 | 26.42 |
|  | Hematological | Cancer cases | 2,538 | 3.60 | 4.04 | 0.03 | 0.98 | 2.55 | 4.69 | 52.51 |
|  | Hematological | Controls | 10,152 | 3.74 | 4.13 | 0.03 | 1.05 | 2.64 | 4.89 | 54.94 |
|  | Non-Hodgkin's lymphoma | Cancer cases | 884 | 3.40 | 3.58 | 0.03 | 0.98 | 2.49 | 4.46 | 29.79 |
|  | Non-Hodgkin's lymphoma | Controls | 3,536 | 3.64 | 3.87 | 0.03 | 1.11 | 2.63 | 4.72 | 45.64 |
|  | Hodgkins lymphoma | Cancer cases | 374 | 2.20 | 2.82 | 0.04 | 0.44 | 1.17 | 2.76 | 18.78 |
|  | Hodgkins lymphoma | Controls | 1,496 | 2.39 | 3.14 | 0.03 | 0.45 | 1.29 | 3.17 | 35.09 |
|  | Multiple myeloma | Cancer cases | 164 | 4.11 | 4.08 | 0.20 | 1.46 | 3.02 | 5.02 | 29.65 |
|  | Multiple myeloma | Controls | 656 | 4.35 | 4.45 | 0.11 | 1.49 | 3.18 | 5.77 | 45.93 |
|  | Leukemia | Cancer cases | 597 | 3.70 | 3.69 | 0.04 | 1.12 | 2.82 | 5.05 | 30.66 |
|  | Leukemia | Controls | 2,388 | 4.08 | 4.50 | 0.05 | 1.24 | 2.93 | 5.35 | 54.94 |
|  | Other heme | Cancer cases | 263 | 4.31 | 4.69 | 0.06 | 1.54 | 3.14 | 5.32 | 30.85 |
|  | Other heme | Controls | 1,052 | 4.21 | 4.45 | 0.04 | 1.42 | 3.07 | 5.38 | 49.20 |
| QStroke | Any cancer | Cancer cases | 31,534 | 4.49 | 3.04 | 0.11 | 2.10 | 4.03 | 6.28 | 40.18 |
|  | Any cancer | Controls | 126,136 | 4.48 | 3.06 | 0.12 | 2.06 | 3.99 | 6.32 | 39.49 |
|  | Breast | Cancer cases | 10,167 | 3.86 | 2.47 | 0.19 | 1.93 | 3.48 | 5.32 | 33.61 |
|  | Breast | Controls | 40,668 | 3.87 | 2.48 | 0.15 | 1.93 | 3.47 | 5.38 | 23.80 |
|  | Lung | Cancer cases | 246 | 5.34 | 3.16 | 0.26 | 2.86 | 4.90 | 7.21 | 17.07 |
|  | Lung | Controls | 984 | 5.39 | 3.25 | 0.24 | 2.91 | 4.97 | 7.39 | 16.70 |
|  | Prostate | Cancer cases | 2,845 | 6.97 | 2.80 | 0.36 | 5.03 | 6.82 | 8.66 | 22.66 |
|  | Prostate | Controls | 11,380 | 7.12 | 2.98 | 0.40 | 5.07 | 6.93 | 8.83 | 35.63 |
|  | Brain/CNS | Cancer cases | 254 | 3.30 | 2.76 | 0.25 | 0.95 | 2.45 | 4.96 | 12.64 |
|  | Brain/CNS | Controls | 1,016 | 3.39 | 2.84 | 0.18 | 1.03 | 2.55 | 5.18 | 16.57 |
|  | Hematological | Cancer cases | 2,538 | 4.50 | 3.24 | 0.18 | 1.88 | 4.05 | 6.39 | 25.29 |
|  | Hematological | Controls | 10,152 | 4.59 | 3.32 | 0.19 | 1.88 | 4.06 | 6.59 | 29.45 |
|  | Non-Hodgkin's lymphoma | Cancer cases | 884 | 4.36 | 3.01 | 0.18 | 1.82 | 3.92 | 6.24 | 17.07 |
|  | Non-Hodgkin's lymphoma | Controls | 3,536 | 4.53 | 3.15 | 0.22 | 1.95 | 4.03 | 6.51 | 22.92 |
|  | Hodgkins lymphoma | Cancer cases | 374 | 3.06 | 2.77 | 0.24 | 0.98 | 2.14 | 4.39 | 15.37 |
|  | Hodgkins lymphoma | Controls | 1,496 | 3.08 | 2.81 | 0.19 | 0.93 | 2.13 | 4.44 | 26.94 |
|  | Multiple myeloma | Cancer cases | 164 | 5.09 | 3.30 | 0.47 | 2.42 | 4.51 | 7.39 | 16.44 |
|  | Multiple myeloma | Controls | 656 | 5.30 | 3.54 | 0.30 | 2.67 | 4.71 | 7.32 | 21.71 |
|  | Leukemia | Cancer cases | 597 | 4.77 | 3.32 | 0.24 | 2.20 | 4.51 | 6.54 | 25.29 |
|  | Leukemia | Controls | 2,388 | 4.94 | 3.42 | 0.19 | 2.23 | 4.52 | 6.98 | 29.45 |
|  | Other heme | Cancer cases | 263 | 5.13 | 3.46 | 0.29 | 2.43 | 4.88 | 7.19 | 20.71 |
|  | Other heme | Controls | 1,052 | 5.09 | 3.32 | 0.24 | 2.45 | 4.76 | 7.07 | 23.94 |
| CHADsVASc | Any cancer | Cancer cases | 31,534 | 1.45 | 0.99 | 0.00 | 1.00 | 1.00 | 2.00 | 7.00 |
|  | Any cancer | Controls | 126,136 | 1.44 | 0.98 | 0.00 | 1.00 | 1.00 | 2.00 | 7.00 |
|  | Breast | Cancer cases | 10,167 | 1.70 | 0.89 | 1.00 | 1.00 | 1.00 | 2.00 | 7.00 |
|  | Breast | Controls | 40,668 | 1.71 | 0.88 | 1.00 | 1.00 | 1.00 | 2.00 | 7.00 |
|  | Lung | Cancer cases | 246 | 1.36 | 1.12 | 0.00 | 1.00 | 1.00 | 2.00 | 6.00 |
|  | Lung | Controls | 984 | 1.45 | 1.13 | 0.00 | 1.00 | 1.00 | 2.00 | 6.00 |
|  | Prostate | Cancer cases | 2,845 | 1.09 | 0.92 | 0.00 | 0.00 | 1.00 | 2.00 | 6.00 |
|  | Prostate | Controls | 11,380 | 1.13 | 0.96 | 0.00 | 0.00 | 1.00 | 2.00 | 5.00 |
|  | Brain/CNS | Cancer cases | 254 | 1.18 | 0.94 | 0.00 | 1.00 | 1.00 | 2.00 | 5.00 |
|  | Brain/CNS | Controls | 1,016 | 1.14 | 0.94 | 0.00 | 1.00 | 1.00 | 2.00 | 5.00 |
|  | Hematological | Cancer cases | 2,538 | 1.30 | 1.06 | 0.00 | 1.00 | 1.00 | 2.00 | 6.00 |
|  | Hematological | Controls | 10,152 | 1.26 | 1.06 | 0.00 | 1.00 | 1.00 | 2.00 | 6.00 |
|  | Non-Hodgkin's lymphoma | Cancer cases | 884 | 1.26 | 1.05 | 0.00 | 1.00 | 1.00 | 2.00 | 5.00 |
|  | Non-Hodgkin's lymphoma | Controls | 3,536 | 1.25 | 1.05 | 0.00 | 1.00 | 1.00 | 2.00 | 6.00 |
|  | Hodgkins lymphoma | Cancer cases | 374 | 1.07 | 0.95 | 0.00 | 0.00 | 1.00 | 2.00 | 5.00 |
|  | Hodgkins lymphoma | Controls | 1,496 | 1.09 | 1.01 | 0.00 | 0.00 | 1.00 | 2.00 | 5.00 |
|  | Multiple myeloma | Cancer cases | 164 | 1.54 | 1.15 | 0.00 | 1.00 | 1.00 | 2.00 | 5.00 |
|  | Multiple myeloma | Controls | 656 | 1.41 | 1.17 | 0.00 | 1.00 | 1.00 | 2.00 | 6.00 |
|  | Leukemia | Cancer cases | 597 | 1.29 | 1.05 | 0.00 | 1.00 | 1.00 | 2.00 | 6.00 |
|  | Leukemia | Controls | 2,388 | 1.30 | 1.02 | 0.00 | 1.00 | 1.00 | 2.00 | 6.00 |
|  | Other heme | Cancer cases | 263 | 1.46 | 1.15 | 0.00 | 1.00 | 1.00 | 2.00 | 5.00 |
|  | Other heme | Controls | 1,052 | 1.38 | 1.13 | 0.00 | 1.00 | 1.00 | 2.00 | 5.00 |

**Supplemental Table 6 footnote:** CVD 1 = combined endpoint including non-fatal myocardial infarction, non-fatal stroke or cardiovascular mortality, where cardiovascular mortality is defined as any death with a primary cause from ICD10 I00-I80. CVD 2 = combined endpoint including everything from CVD 1 plus incident atrial fibrillation, heart failure, non-ischaemic cardiomyopathies and valvular heart disease; CNS = central nervous system; SD: standard deviation.

## Supplemental Table 7: Any cancer

| **Outcome** | **Risk score** | **Group** | **Events within 10 years** | **Cumulative incidence** | **Difference in incidence between cancer and controls** | **Average risk score** | **Difference between observed incidence and risk score** | **AUC** | **Balanced accuracy** | **Sensitivity** |
| --- | --- | --- | --- | --- | --- | --- | --- | --- | --- | --- |
| **CVD 1** | QRISK3 | Cancer | 1,542 (4.9%) | 4.9* [4.7, 5.1] | 0.6* [0.3, 0.9] | 12.0 [11.9, 12.1] | 7.2* [6.9, 7.4] | 0.70* [0.69, 0.71] | 0.65* [0.64, 0.66] | 0.62* [0.60, 0.64] |
|  |  | Controls | 5,423 (4.3%) | 4.3* [4.2, 4.4] | 0.6* [0.3, 0.9] | 12.0 [12.0, 12.1] | 7.7* [7.6, 7.8] | 0.73* [0.73, 0.73] | 0.67* [0.67, 0.67] | 0.66* [0.65, 0.67] |
|  | Framingham (BMI) | Cancer | 1,542 (4.9%) | 4.9* [4.7, 5.1] | 0.6* [0.3, 0.9] | 17.7 [17.6, 17.9] | 12.8* [12.6, 13.1] | 0.69* [0.68, 0.71] | 0.65* [0.64, 0.66] | 0.73 [0.71, 0.75] |
|  |  | Controls | 5,423 (4.3%) | 4.3* [4.2, 4.4] | 0.6* [0.3, 0.9] | 17.8 [17.7, 17.9] | 13.5* [13.4, 13.6] | 0.72* [0.72, 0.72] | 0.66* [0.66, 0.66] | 0.75 [0.75, 0.76] |
|  | Framingham (blood) | Cancer | 1,542 (4.9%) | 4.9* [4.7, 5.1] | 0.6* [0.3, 0.9] | 15.7 [15.6, 15.8] | 10.8* [10.5, 11.1] | 0.70* [0.68, 0.71] | 0.65* [0.64, 0.66] | 0.68* [0.66, 0.71] |
|  |  | Controls | 5,423 (4.3%) | 4.3* [4.2, 4.4] | 0.6* [0.3, 0.9] | 15.8 [15.7, 15.8] | 11.5* [11.3, 11.6] | 0.72* [0.72, 0.72] | 0.66* [0.66, 0.67] | 0.71* [0.71, 0.72] |
|  | SCORE2 / | Cancer | 1,542 (4.9%) | 4.9* [4.7, 5.1] | 0.6* [0.3, 0.9] | 5.3 [5.2, 5.3] | 0.4* [0.1, 0.6] | 0.70* [0.68, 0.71] | 0.65* [0.64, 0.66] | 0.71* [0.69, 0.73] |
|  | SCORE-OP | Controls | 5,423 (4.3%) | 4.3* [4.2, 4.4] | 0.6* [0.3, 0.9] | 5.3 [5.3, 5.3] | 1.0* [0.9, 1.1] | 0.72* [0.72, 0.72] | 0.66* [0.66, 0.67] | 0.74* [0.73, 0.75] |
| **CVD 2** | QRISK3 | Cancer | 3,636 (11.5%) | 11.5* [11.2, 11.9] | 1.9* [1.6, 2.3] | 12.0 [11.9, 12.1] | 0.5* [0.1, 0.9] | 0.68* [0.67, 0.69] | 0.64* [0.63, 0.64] | 0.64* [0.63, 0.66] |
|  |  | Controls | 12,087 (9.6%) | 9.6* [9.4, 9.7] | 1.9* [1.6, 2.3] | 12.0 [12.0, 12.1] | 2.4* [2.3, 2.6] | 0.72* [0.72, 0.72] | 0.66* [0.66, 0.66] | 0.69* [0.68, 0.69] |
|  | Framingham (BMI) | Cancer | 3,636 (11.5%) | 11.5* [11.2, 11.9] | 1.9* [1.6, 2.3] | 17.7 [17.6, 17.9] | 6.2* [5.8, 6.6] | 0.68* [0.67, 0.69] | 0.63* [0.62, 0.64] | 0.67* [0.66, 0.69] |
|  |  | Controls | 12,087 (9.6%) | 9.6* [9.4, 9.7] | 1.9* [1.6, 2.3] | 17.8 [17.7, 17.9] | 8.2* [8.1, 8.4] | 0.71* [0.71, 0.71] | 0.66* [0.65, 0.66] | 0.72* [0.72, 0.73] |
|  | Framingham (blood) | Cancer | 3,636 (11.5%) | 11.5* [11.2, 11.9] | 1.9* [1.6, 2.3] | 15.7 [15.6, 15.8] | 4.2* [3.8, 4.5] | 0.67* [0.66, 0.68] | 0.62* [0.61, 0.63] | 0.66* [0.64, 0.67] |
|  |  | Controls | 12,087 (9.6%) | 9.6* [9.4, 9.7] | 1.9* [1.6, 2.3] | 15.8 [15.7, 15.8] | 6.2* [6.0, 6.4] | 0.70* [0.70, 0.70] | 0.65* [0.65, 0.65] | 0.71* [0.70, 0.71] |
|  | SCORE2 / | Cancer | 3,636 (11.5%) | 11.5* [11.2, 11.9] | 1.9* [1.6, 2.3] | 5.3 [5.2, 5.3] | -6.3* [-6.6, -5.9] | 0.67* [0.67, 0.68] | 0.63* [0.62, 0.64] | 0.66* [0.65, 0.67] |
|  | SCORE-OP | Controls | 12,087 (9.6%) | 9.6* [9.4, 9.7] | 1.9* [1.6, 2.3] | 5.3 [5.3, 5.3] | -4.3* [-4.5, -4.1] | 0.71* [0.71, 0.71] | 0.65* [0.65, 0.66] | 0.71* [0.71, 0.71] |
| **Atrial** | CHARGE-AF | Cancer | 1,707 (5.4%) | 5.4* [5.2, 5.7] | 0.9* [0.6, 1.2] | 2.0 [2.0, 2.0] | -3.4* [-3.7, -3.2] | 0.71* [0.70, 0.72] | 0.65* [0.64, 0.66] | 0.69* [0.67, 0.71] |
| **fibrillation** |  | Controls | 5,693 (4.5%) | 4.5* [4.4, 4.6] | 0.9* [0.6, 1.2] | 2.0 [2.0, 2.0] | -2.5* [-2.6, -2.4] | 0.74* [0.74, 0.74] | 0.68* [0.67, 0.68] | 0.74* [0.74, 0.75] |
| **Heart** | PCP-HF | Cancer | 789 (2.5%) | 2.5* [2.3, 2.7] | 0.8* [0.6, 0.9] | 3.3 [3.3, 3.4] | 0.8* [0.7, 1.0] | 0.71* [0.69, 0.72] | 0.65* [0.64, 0.67] | 0.73* [0.70, 0.76] |
| **failure** |  | Controls | 2,204 (1.7%) | 1.7* [1.7, 1.8] | 0.8* [0.6, 0.9] | 3.4 [3.3, 3.4] | 1.6* [1.5, 1.7] | 0.76* [0.76, 0.76] | 0.69* [0.69, 0.70] | 0.81* [0.80, 0.81] |
| **Stroke** | QStroke | Cancer | 664 (2.1%) | 2.1* [2.0, 2.3] | 0.5* [0.3, 0.6] | 4.5 [4.5, 4.5] | 2.4* [2.2, 2.6] | 0.66* [0.64, 0.68] | 0.62* [0.61, 0.64] | 0.67* [0.64, 0.71] |
|  |  | Controls | 2,072 (1.6%) | 1.6* [1.6, 1.7] | 0.5* [0.3, 0.6] | 4.5 [4.5, 4.5] | 2.8* [2.8, 2.9] | 0.70* [0.70, 0.70] | 0.65* [0.65, 0.66] | 0.73* [0.72, 0.74] |
|  | CHADsVASc | Cancer | 664 (2.1%) | 2.1* [2.0, 2.3] | 0.5* [0.3, 0.6] | 1.5 [1.4, 1.5] |  | 0.57* [0.54, 0.59] | 0.56* [0.54, 0.58] | 0.52* [0.48, 0.56] |
|  |  | Controls | 2,072 (1.6%) | 1.6* [1.6, 1.7] | 0.5* [0.3, 0.6] | 1.4 [1.4, 1.4] |  | 0.59* [0.59, 0.59] | 0.59* [0.58, 0.59] | 0.57* [0.56, 0.58] |

**Supplemental Table 7 footnote:** Table shows the event counts, ten-year cumulative incidence, average risk score and predictive performance statistics. Risk score means are given for each outcome-cancer-risk-score combination. Cumulative incidence and 95% confidence intervals are from Fine-Gray models accounting for the competing risk of death at ten years follow-up. The column marked in yellow indicates calibration differences between the risk score and the observed incidence, where the asterisk (*) indicates where this difference is significantly different from zero. All other asterisks indicate significant differences between cancer cases and controls. The final three columns reflect performance scoring of the candidate risk scores in cancer and control groups. Area under the receiver operating curve (AUC) is calculated from time-dependent analyses adjusted by the competing risk of death. Balanced accuracy and sensitivity are binary metrics calculated using ten-year outcomes, with confidence intervals derived from bootstrapping and permutation testing with 1000 replicates. Calibration is not possible for CHADsVASc as this is an ordinal scale. CVD 1 = combined endpoint including non-fatal myocardial infarction, non-fatal stroke or cardiovascular mortality, where cardiovascular mortality is defined as any death with a primary cause from ICD10 I00-I80. CVD 2 = combined endpoint including everything from CVD 1 plus incident atrial fibrillation, heart failure, non-ischaemic cardiomyopathies and valvular heart disease.

## Supplemental Figure 4: Cumulative Incidence Function Plot- Any cancer


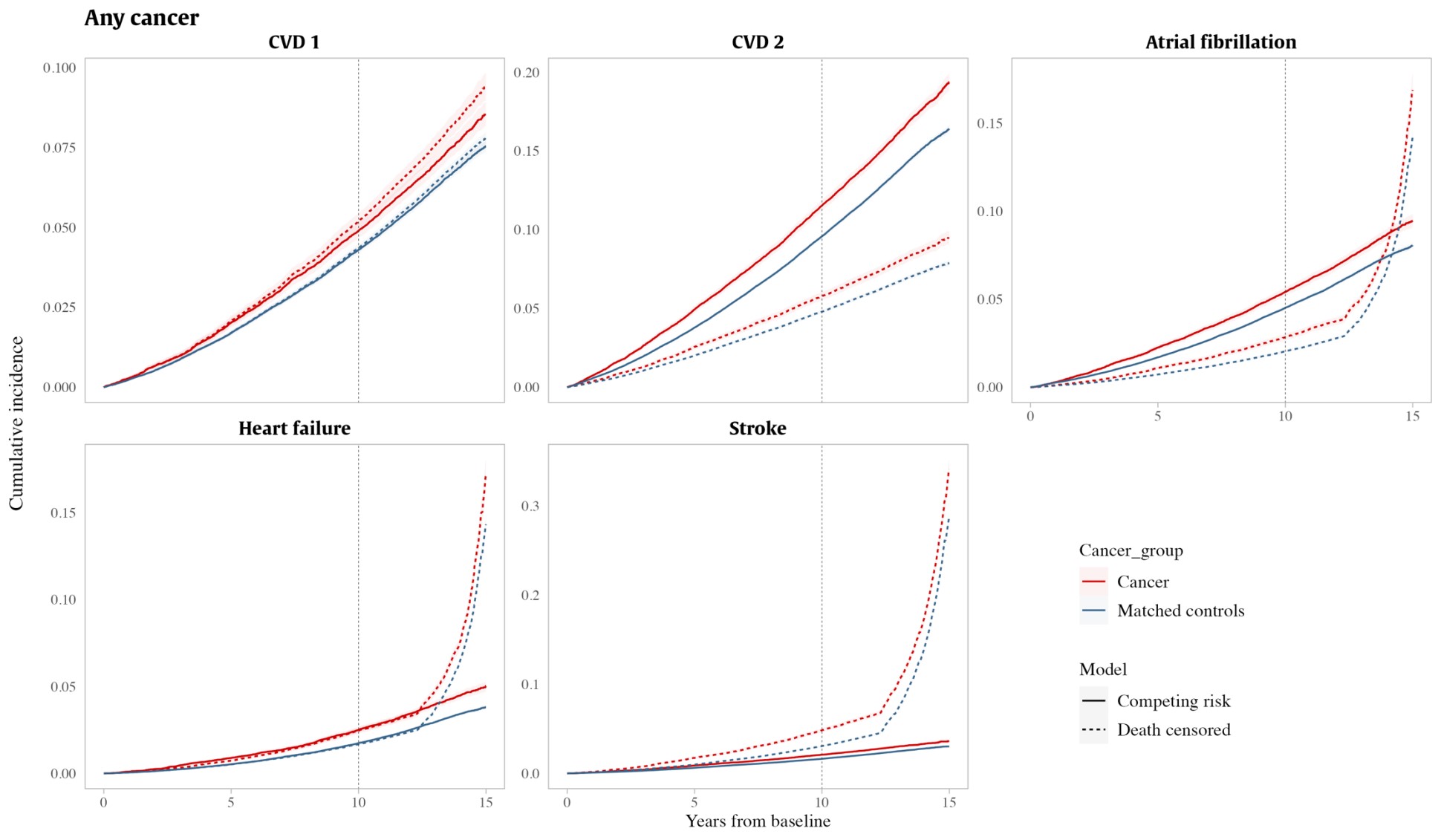


**Supplemental Figure 4 footnote:** Cumulative incidence functions and 95% confidence intervals across follow-up for Fine-Gray models adjusting for competing risk of death (solid lines) along with incidence with death censoring (dotted lines). Cancer cases are shown in red, with Controls in dark blue. Where sample sizes are very large the shaded 95% confidence intervals may be too narrow to visualise clearly. The vertical dotted line indicates ten-year follow-up.

## Supplemental Figure 5: Calibration plots – Any cancer


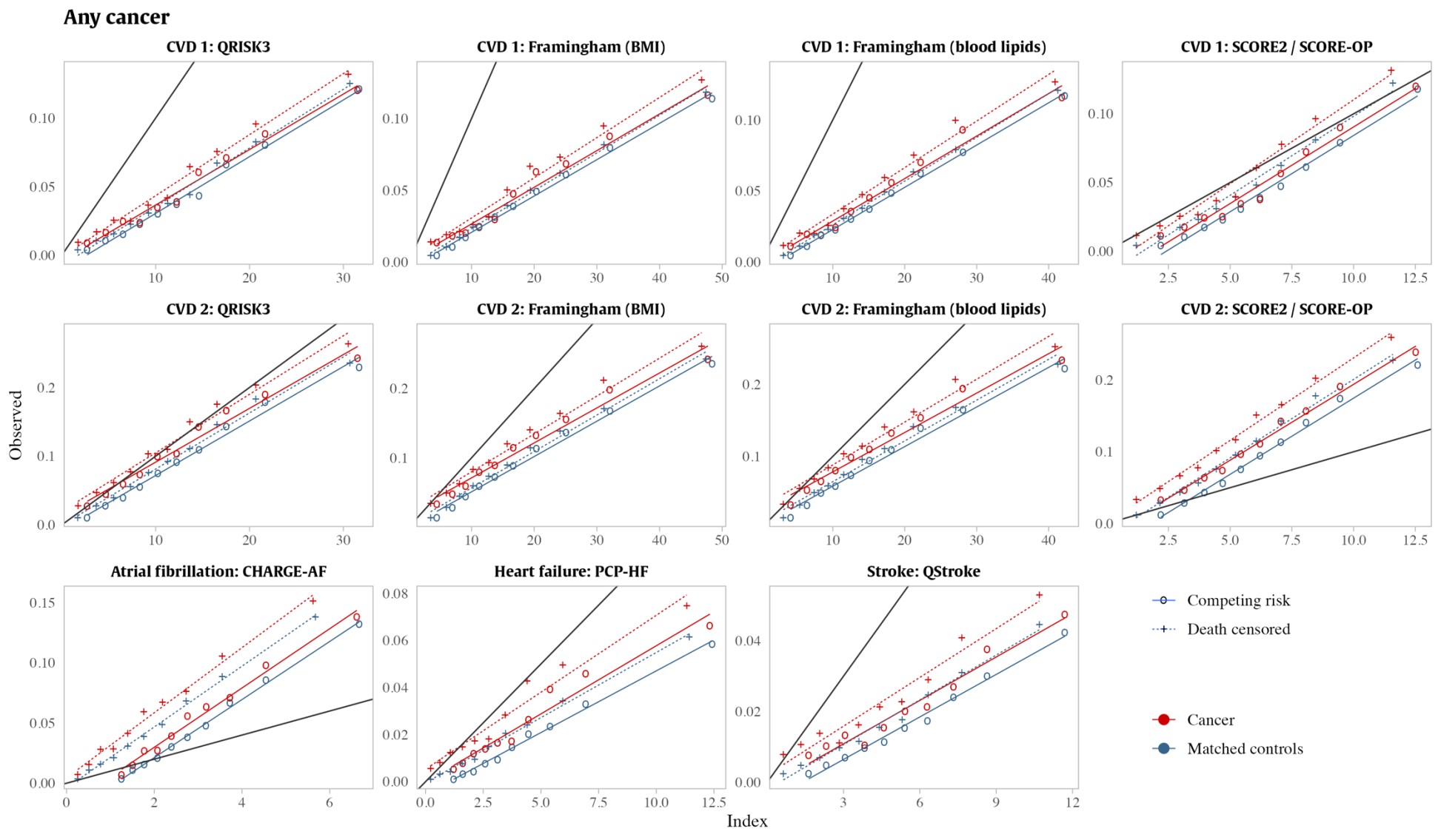


**Supplemental Figure 5 footnote:** Calibration plots for outcome-risk score combinations at ten-year follow-up, where the risk score values are shown on the x-axis, and observed decile means are plotted on the y-axis. Observed outcomes accounting for competing risk of death are shown with ‘o’ and solid lines, and outcomes with death censoring are shown with ‘+’ and dotted lines. Linear best fit lines are given to show the trend of the relationship between risk score and observed outcomes, with cancer cases in red and Controls in dark blue. The solid black line indicates where the perfect agreement of risk score and outcomes would fall. Calibration statistics are not possible for CHADsVASc as this is an ordinal scale_._

## Supplemental Table 8: Breast cancer

| **Outcome** | **Index** | **Group** | **Events within 10 years** | **Cumulative incidence** | **Difference in incidence between cancer and controls** | **Average risk score** | **Difference between observed incidence and risk score** | **AUC** | **Balanced accuracy** | **Sensitivity** |
| --- | --- | --- | --- | --- | --- | --- | --- | --- | --- | --- |
| **CVD 1** | QRISK3 | Cancer | 302 (3.0%) | 3.0 [2.7, 3.3] | 0.0 [-0.4, 0.4] | 9.3 [9.1, 9.4] | 6.3* [5.9, 6.7] | 0.68 [0.65, 0.71] | 0.63 [0.61, 0.65] | 0.63 [0.58, 0.67] |
|  |  | Controls | 1,199 (2.9%) | 2.9 [2.8, 3.1] | 0.0 [-0.4, 0.4] | 9.3 [9.2, 9.3] | 6.3* [6.2, 6.5] | 0.71 [0.69, 0.72] | 0.65 [0.65, 0.66] | 0.67 [0.66, 0.68] |
|  | Framingham (BMI) | Cancer | 302 (3.0%) | 3.0 [2.7, 3.3] | 0.0 [-0.4, 0.4] | 12.9 [12.7, 13.1] | 9.9* [9.6, 10.3] | 0.67 [0.64, 0.70] | 0.63 [0.60, 0.65] | 0.69 [0.65, 0.74] |
|  |  | Controls | 1,199 (2.9%) | 2.9 [2.8, 3.1] | 0.0 [-0.4, 0.4] | 12.9 [12.8, 13.0] | 9.9* [9.8, 10.1] | 0.69 [0.68, 0.71] | 0.64 [0.63, 0.64] | 0.71 [0.70, 0.72] |
|  | Framingham (blood) | Cancer | 302 (3.0%) | 3.0 [2.7, 3.3] | 0.0 [-0.4, 0.4] | 11.6 [11.5, 11.8] | 8.7* [8.3, 9.0] | 0.67 [0.64, 0.70] | 0.63 [0.61, 0.66] | 0.69 [0.64, 0.73] |
|  |  | Controls | 1,199 (2.9%) | 2.9 [2.8, 3.1] | 0.0 [-0.4, 0.4] | 11.6 [11.6, 11.7] | 8.7* [8.5, 8.9] | 0.69 [0.67, 0.70] | 0.64 [0.63, 0.64] | 0.70 [0.69, 0.71] |
|  | SCORE2 / | Cancer | 302 (3.0%) | 3.0 [2.7, 3.3] | 0.0 [-0.4, 0.4] | 4.3 [4.3, 4.4] | 1.3* [1.0, 1.7] | 0.68 [0.65, 0.71] | 0.64 [0.62, 0.67] | 0.72 [0.67, 0.76] |
|  | SCORE-OP | Controls | 1,199 (2.9%) | 2.9 [2.8, 3.1] | 0.0 [-0.4, 0.4] | 4.3 [4.3, 4.3] | 1.4* [1.2, 1.5] | 0.70 [0.68, 0.71] | 0.65 [0.64, 0.66] | 0.72 [0.71, 0.74] |
| **CVD 2** | QRISK3 | Cancer | 847 (8.3%) | 8.3* [7.8, 8.9] | 1.1* [0.5, 1.7] | 9.3 [9.1, 9.4] | 0.9* [0.4, 1.5] | 0.67* [0.65, 0.69] | 0.62* [0.61, 0.64] | 0.65* [0.62, 0.68] |
|  |  | Controls | 2,935 (7.2%) | 7.2* [7.0, 7.5] | 1.1* [0.5, 1.7] | 9.3 [9.2, 9.3] | 2.1* [1.8, 2.3] | 0.70* [0.70, 0.71] | 0.65* [0.65, 0.65] | 0.70* [0.70, 0.71] |
|  | Framingham (BMI) | Cancer | 847 (8.3%) | 8.3* [7.8, 8.9] | 1.1* [0.5, 1.7] | 12.9 [12.7, 13.1] | 4.6* [4.0, 5.1] | 0.66* [0.64, 0.68] | 0.62 [0.61, 0.64] | 0.63 [0.60, 0.66] |
|  |  | Controls | 2,935 (7.2%) | 7.2* [7.0, 7.5] | 1.1* [0.5, 1.7] | 12.9 [12.8, 13.0] | 5.7* [5.4, 5.9] | 0.69* [0.68, 0.70] | 0.64 [0.63, 0.64] | 0.66 [0.66, 0.67] |
|  | Framingham (blood) | Cancer | 847 (8.3%) | 8.3* [7.8, 8.9] | 1.1* [0.5, 1.7] | 11.6 [11.5, 11.8] | 3.3* [2.7, 3.9] | 0.64* [0.62, 0.66] | 0.60* [0.58, 0.61] | 0.63* [0.60, 0.66] |
|  |  | Controls | 2,935 (7.2%) | 7.2* [7.0, 7.5] | 1.1* [0.5, 1.7] | 11.6 [11.6, 11.7] | 4.4* [4.1, 4.7] | 0.67* [0.66, 0.68] | 0.63* [0.62, 0.63] | 0.68* [0.68, 0.69] |
|  | SCORE2 / | Cancer | 847 (8.3%) | 8.3* [7.8, 8.9] | 1.1* [0.5, 1.7] | 4.3 [4.3, 4.4] | -4.0* [-4.6, -3.5] | 0.66* [0.64, 0.68] | 0.62* [0.61, 0.64] | 0.66* [0.63, 0.69] |
|  | SCORE-OP | Controls | 2,935 (7.2%) | 7.2* [7.0, 7.5] | 1.1* [0.5, 1.7] | 4.3 [4.3, 4.3] | -2.9* [-3.2, -2.7] | 0.69* [0.68, 0.70] | 0.65* [0.64, 0.65] | 0.71* [0.70, 0.71] |
| **Atrial** | CHARGE-AF | Cancer | 397 (3.9%) | 3.9* [3.5, 4.3] | 0.6* [0.2, 1.0] | 1.6 [1.6, 1.6] | -2.3* [-2.7, -1.9] | 0.70 [0.68, 0.73] | 0.65 [0.63, 0.67] | 0.73 [0.69, 0.77] |
| **fibrillation** |  | Controls | 1,350 (3.3%) | 3.3* [3.1, 3.5] | 0.6* [0.2, 1.0] | 1.6 [1.6, 1.6] | -1.7* [-1.9, -1.5] | 0.73 [0.72, 0.74] | 0.67 [0.66, 0.67] | 0.76 [0.75, 0.77] |
| **Heart** | PCP-HF | Cancer | 188 (1.8%) | 1.8* [1.6, 2.1] | 0.5* [0.2, 0.8] | 2.4 [2.3, 2.4] | 0.5* [0.3, 0.8] | 0.71 [0.67, 0.75] | 0.67 [0.63, 0.70] | 0.64 [0.57, 0.71] |
| **failure** |  | Controls | 530 (1.3%) | 1.3* [1.2, 1.4] | 0.5* [0.2, 0.8] | 2.4 [2.4, 2.4] | 1.1* [1.0, 1.2] | 0.75 [0.73, 0.77] | 0.69 [0.68, 0.70] | 0.70 [0.68, 0.72] |
| **Stroke** | QStroke | Cancer | 151 (1.5%) | 1.5 [1.3, 1.7] | 0.2 [-0.1, 0.5] | 3.9 [3.8, 3.9] | 2.4* [2.1, 2.6] | 0.66 [0.61, 0.70] | 0.61 [0.58, 0.65] | 0.63 [0.56, 0.70] |
|  |  | Controls | 532 (1.3%) | 1.3 [1.2, 1.4] | 0.2 [-0.1, 0.5] | 3.9 [3.8, 3.9] | 2.6* [2.4, 2.7] | 0.69 [0.67, 0.71] | 0.65 [0.64, 0.66] | 0.71 [0.69, 0.72] |
|  | CHADsVASc | Cancer | 151 (1.5%) | 1.5 [1.3, 1.7] | 0.2 [-0.1, 0.5] | 1.7 [1.7, 1.7] |  | 0.62 [0.58, 0.67] | 0.60 [0.56, 0.63] | 0.68 [0.61, 0.74] |
|  |  | Controls | 532 (1.3%) | 1.3 [1.2, 1.4] | 0.2 [-0.1, 0.5] | 1.7 [1.7, 1.7] |  | 0.64 [0.62, 0.66] | 0.62 [0.61, 0.63] | 0.72 [0.71, 0.74] |

**Supplemental Table 8 footnote:** Table shows the event counts, ten-year cumulative incidence, average risk score and predictive performance statistics. Risk score means are given for each outcome-cancer-risk-score combination. Cumulative incidence and 95% confidence intervals are from Fine-Gray models accounting for the competing risk of death at ten years follow-up. The column marked in yellow indicates calibration differences between the risk score and the observed incidence, where the asterisk (*) indicates where this difference is significantly different from zero. All other asterisks indicate significant differences between cancer cases and controls. The final three columns reflect performance scoring of the candidate risk scores in cancer and control groups. Area under the receiver operating curve (AUC) is calculated from time-dependent analyses adjusted by the competing risk of death. Balanced accuracy and sensitivity are binary metrics calculated using ten-year outcomes, with confidence intervals derived from bootstrapping and permutation testing with 1000 replicates. Calibration is not possible for CHADsVASc as this is an ordinal scale. CVD 1 = combined endpoint including non-fatal myocardial infarction, non-fatal stroke or cardiovascular mortality, where cardiovascular mortality is defined as any death with a primary cause from ICD10 I00-I80. CVD 2 = combined endpoint including everything from CVD 1 plus incident atrial fibrillation, heart failure, non-ischaemic cardiomyopathies and valvular heart disease.

## Supplemental Table 9: Lung cancer

| **Outcome** | **Index** | **Group** | **Events within 10 years** | **Cumulative incidence** | **Difference in incidence between cancer and controls** | **Average risk score** | **Difference between observed incidence and risk score** | **AUC** | **Balanced accuracy** | **Sensitivity** |
| --- | --- | --- | --- | --- | --- | --- | --- | --- | --- | --- |
| **CVD 1** | QRISK3 | Cancer | 20 (8.1%) | 8.1 [5.1, 12.0] | 2.0 [-2.1, 6.2] | 15.0 [13.8, 16.1] | 6.8* [2.8, 10.9] | 0.61 [0.49, 0.72] | 0.58 [0.47, 0.69] | 0.55 [0.34, 0.76] |
|  |  | Controls | 60 (6.1%) | 6.1 [4.7, 7.7] | 2.0 [-2.1, 6.2] | 14.9 [14.3, 15.5] | 8.8* [7.1, 10.5] | 0.72 [0.65, 0.78] | 0.69 [0.67, 0.72] | 0.75 [0.70, 0.80] |
|  | Framingham (BMI) | Cancer | 20 (8.1%) | 8.1 [5.1, 12.0] | 2.0 [-2.1, 6.2] | 20.7 [18.9, 22.4] | 12.5* [8.3, 16.8] | 0.53 [0.41, 0.64] | 0.46* [0.34, 0.58] | 0.20* [0.00, 0.43] |
|  |  | Controls | 60 (6.1%) | 6.1 [4.7, 7.7] | 2.0 [-2.1, 6.2] | 22.6 [21.6, 23.5] | 16.5* [14.6, 18.3] | 0.70 [0.64, 0.77] | 0.68* [0.65, 0.71] | 0.67* [0.61, 0.72] |
|  | Framingham (blood) | Cancer | 20 (8.1%) | 8.1 [5.1, 12.0] | 2.0 [-2.1, 6.2] | 18.4 [16.8, 20.0] | 10.3* [6.1, 14.4] | 0.54 [0.41, 0.67] | 0.56 [0.45, 0.67] | 0.50 [0.29, 0.71] |
|  |  | Controls | 60 (6.1%) | 6.1 [4.7, 7.7] | 2.0 [-2.1, 6.2] | 19.7 [18.9, 20.5] | 13.6* [11.8, 15.4] | 0.69 [0.62, 0.76] | 0.66 [0.63, 0.68] | 0.72 [0.66, 0.77] |
|  | SCORE2 / | Cancer | 20 (8.1%) | 8.1 [5.1, 12.0] | 2.0 [-2.1, 6.2] | 6.2 [5.8, 6.6] | -2.0 [-5.8, 1.9] | 0.60 [0.48, 0.71] | 0.63 [0.54, 0.72] | 0.75 [0.58, 0.92] |
|  | SCORE-OP | Controls | 60 (6.1%) | 6.1 [4.7, 7.7] | 2.0 [-2.1, 6.2] | 6.3 [6.1, 6.5] | 0.2 [-1.5, 1.8] | 0.70 [0.64, 0.77] | 0.66 [0.64, 0.68] | 0.85 [0.81, 0.89] |
| **CVD 2** | QRISK3 | Cancer | 52 (21.1%) | 21.1* [16.3, 26.5] | 7.7* [2.0, 13.5] | 15.0 [13.8, 16.1] | -6.2* [-11.6, -0.7] | 0.63 [0.55, 0.71] | 0.60 [0.52, 0.67] | 0.63 [0.50, 0.77] |
|  |  | Controls | 132 (13.4%) | 13.4* [11.4, 15.6] | 7.7* [2.0, 13.5] | 14.9 [14.3, 15.5] | 1.5 [-0.8, 3.8] | 0.70 [0.65, 0.74] | 0.68 [0.66, 0.70] | 0.79 [0.75, 0.82] |
|  | Framingham (BMI) | Cancer | 52 (21.1%) | 21.1* [16.3, 26.5] | 7.7* [2.0, 13.5] | 20.7 [18.9, 22.4] | -0.5 [-6.1, 5.1] | 0.61 [0.52, 0.69] | 0.55* [0.48, 0.63] | 0.38* [0.25, 0.52] |
|  |  | Controls | 132 (13.4%) | 13.4* [11.4, 15.6] | 7.7* [2.0, 13.5] | 22.6 [21.6, 23.5] | 9.2* [6.7, 11.6] | 0.70 [0.66, 0.75] | 0.67* [0.66, 0.69] | 0.64* [0.61, 0.68] |
|  | Framingham (blood) | Cancer | 52 (21.1%) | 21.1* [16.3, 26.5] | 7.7* [2.0, 13.5] | 18.4 [16.8, 20.0] | -2.7 [-8.3, 2.8] | 0.60 [0.52, 0.69] | 0.59 [0.51, 0.66] | 0.54* [0.40, 0.68] |
|  |  | Controls | 132 (13.4%) | 13.4* [11.4, 15.6] | 7.7* [2.0, 13.5] | 19.7 [18.9, 20.5] | 6.2* [3.9, 8.6] | 0.70 [0.65, 0.74] | 0.65 [0.63, 0.67] | 0.71* [0.68, 0.75] |
|  | SCORE2 / | Cancer | 52 (21.1%) | 21.1* [16.3, 26.5] | 7.7* [2.0, 13.5] | 6.2 [5.8, 6.6] | -15.0* [-20.3, -9.6] | 0.65 [0.56, 0.73] | 0.63 [0.56, 0.69] | 0.71 [0.59, 0.83] |
|  | SCORE-OP | Controls | 132 (13.4%) | 13.4* [11.4, 15.6] | 7.7* [2.0, 13.5] | 6.3 [6.1, 6.5] | -7.1* [-9.4, -4.9] | 0.69 [0.64, 0.74] | 0.66 [0.64, 0.67] | 0.82 [0.79, 0.85] |
| **Atrial** | CHARGE-AF | Cancer | 29 (11.8%) | 11.8* [8.1, 16.2] | 5.5* [0.8, 10.2] | 2.3 [2.0, 2.5] | -9.5* [-13.9, -5.1] | 0.71 [0.62, 0.80] | 0.68 [0.60, 0.77] | 0.79 [0.64, 0.95] |
| **fibrillation** |  | Controls | 62 (6.3%) | 6.3* [4.9, 7.9] | 5.5* [0.8, 10.2] | 2.5 [2.4, 2.6] | -3.8* [-5.4, -2.1] | 0.68 [0.62, 0.74] | 0.65 [0.63, 0.67] | 0.82 [0.78, 0.86] |
| **Heart** | PCP-HF | Cancer | 17 (6.9%) | 6.9 [4.2, 10.5] | 3.8 [-0.1, 7.6] | 4.0 [3.5, 4.4] | -3.0 [-6.6, 0.7] | 0.65 [0.51, 0.79] | 0.62 [0.49, 0.76] | 0.59 [0.32, 0.85] |
| **failure** |  | Controls | 31 (3.2%) | 3.2 [2.2, 4.4] | 3.8 [-0.1, 7.6] | 4.3 [4.1, 4.5] | 1.1 [-0.1, 2.4] | 0.77 [0.71, 0.84] | 0.72 [0.69, 0.75] | 0.81 [0.74, 0.87] |
| **Stroke** | QStroke | Cancer | 7 (2.8%) | 2.8 [1.3, 5.5] | 0.9 [-1.9, 3.8] | 5.3 [4.9, 5.7] | 2.5 [-0.2, 5.2] | 0.56 [0.44, 0.68] | 0.65 [0.50, 0.81] | 0.86 [0.55, 1.00] |
|  |  | Controls | 19 (1.9%) | 1.9 [1.2, 2.9] | 0.9 [-1.9, 3.8] | 5.4 [5.2, 5.6] | 3.5* [2.4, 4.5] | 0.66 [0.54, 0.78] | 0.64 [0.60, 0.68] | 0.84 [0.77, 0.92] |
|  | CHADsVASc | Cancer | 7 (2.8%) | 2.8 [1.3, 5.5] | 0.9 [-1.9, 3.8] | 1.4 [1.2, 1.5] |  | 0.60 [0.36, 0.83] | 0.47 [0.35, 0.59] | 0.71 [0.48, 0.95] |
|  |  | Controls | 19 (1.9%) | 1.9 [1.2, 2.9] | 0.9 [-1.9, 3.8] | 1.4 [1.4, 1.5] |  | 0.59 [0.49, 0.69] | 0.60 [0.57, 0.62] | 1.00 [0.95, 1.00] |

**Supplemental Table 9 footnote:** Table shows the event counts, ten-year cumulative incidence, average risk score and predictive performance statistics. Risk score means are given for each outcome-cancer-risk-score combination. Cumulative incidence and 95% confidence intervals are from Fine-Gray models accounting for the competing risk of death at ten years follow-up. The column marked in yellow indicates calibration differences between the risk score and the observed incidence, where the asterisk (*) indicates where this difference is significantly different from zero. All other asterisks indicate significant differences between cancer cases and controls. The final three columns reflect performance scoring of the candidate risk scores in cancer and control groups. Area under the receiver operating curve (AUC) is calculated from time-dependent analyses adjusted by the competing risk of death. Balanced accuracy and sensitivity are binary metrics calculated using ten-year outcomes, with confidence intervals derived from bootstrapping and permutation testing with 1000 replicates. Calibration is not possible for CHADsVASc as this is an ordinal scale. CVD 1 = combined endpoint including non-fatal myocardial infarction, non-fatal stroke or cardiovascular mortality, where cardiovascular mortality is defined as any death with a primary cause from ICD10 I00-I80. CVD 2 = combined endpoint including everything from CVD 1 plus incident atrial fibrillation, heart failure, non-ischaemic cardiomyopathies and valvular heart disease.

## Supplemental Table 10: Prostate cancer

| **Outcome** | **Index** | **Group** | **Events within 10 years** | **Cumulative incidence** | **Difference in incidence between cancer and controls** | **Average risk score** | **Difference between observed incidence and risk score** | **AUC** | **Balanced accuracy** | **Sensitivity** |
| --- | --- | --- | --- | --- | --- | --- | --- | --- | --- | --- |
| **CVD 1** | QRISK3 | Cancer | 218 (7.7%) | 7.7 [6.7, 8.7] | -0.3 [-1.4, 0.9] | 20.2 [19.9, 20.5] | 12.5* [11.5, 13.6] | 0.63 [0.59, 0.67] | 0.59 [0.56, 0.62] | 0.47 [0.41, 0.53] |
|  |  | Controls | 901 (7.9%) | 7.9 [7.4, 8.4] | -0.3 [-1.4, 0.9] | 20.5 [20.4, 20.7] | 12.6* [12.1, 13.1] | 0.64 [0.62, 0.66] | 0.61 [0.60, 0.61] | 0.51 [0.50, 0.53] |
|  | Framingham (BMI) | Cancer | 218 (7.7%) | 7.7 [6.7, 8.7] | -0.3 [-1.4, 0.9] | 31.4 [30.9, 31.8] | 23.7* [22.6, 24.8] | 0.61 [0.58, 0.65] | 0.56 [0.54, 0.59] | 0.56* [0.50, 0.62] |
|  |  | Controls | 901 (7.9%) | 7.9 [7.4, 8.4] | -0.3 [-1.4, 0.9] | 32.3 [32.0, 32.5] | 24.4* [23.8, 24.9] | 0.62 [0.60, 0.64] | 0.59 [0.58, 0.60] | 0.63* [0.62, 0.65] |
|  | Framingham (blood) | Cancer | 218 (7.7%) | 7.7 [6.7, 8.7] | -0.3 [-1.4, 0.9] | 27.3 [26.8, 27.7] | 19.6* [18.5, 20.7] | 0.63 [0.59, 0.67] | 0.57 [0.54, 0.60] | 0.41 [0.36, 0.47] |
|  |  | Controls | 901 (7.9%) | 7.9 [7.4, 8.4] | -0.3 [-1.4, 0.9] | 27.9 [27.7, 28.1] | 20.0* [19.4, 20.5] | 0.63 [0.61, 0.65] | 0.59 [0.58, 0.60] | 0.47 [0.45, 0.48] |
|  | SCORE2 / | Cancer | 218 (7.7%) | 7.7 [6.7, 8.7] | -0.3 [-1.4, 0.9] | 8.3 [8.2, 8.4] | 0.7 [-0.3, 1.7] | 0.62 [0.58, 0.66] | 0.57 [0.54, 0.60] | 0.52* [0.46, 0.58] |
|  | SCORE-OP | Controls | 901 (7.9%) | 7.9 [7.4, 8.4] | -0.3 [-1.4, 0.9] | 8.5 [8.4, 8.5] | 0.6* [0.1, 1.1] | 0.63 [0.62, 0.65] | 0.60 [0.59, 0.61] | 0.60* [0.59, 0.62] |
| **CVD 2** | QRISK3 | Cancer | 464 (16.3%) | 16.3 [15.0, 17.7] | -1.0 [-2.6, 0.5] | 20.2 [19.9, 20.5] | 3.9* [2.5, 5.3] | 0.61 [0.58, 0.64] | 0.57 [0.55, 0.59] | 0.62 [0.58, 0.66] |
|  |  | Controls | 1,974 (17.3%) | 17.3 [16.7, 18.0] | -1.0 [-2.6, 0.5] | 20.5 [20.4, 20.7] | 3.2* [2.4, 3.9] | 0.61 [0.60, 0.62] | 0.58 [0.57, 0.59] | 0.65 [0.64, 0.66] |
|  | Framingham (BMI) | Cancer | 464 (16.3%) | 16.3 [15.0, 17.7] | -1.0 [-2.6, 0.5] | 31.4 [30.9, 31.8] | 15.1* [13.6, 16.5] | 0.61 [0.58, 0.64] | 0.56 [0.54, 0.58] | 0.57* [0.53, 0.61] |
|  |  | Controls | 1,974 (17.3%) | 17.3 [16.7, 18.0] | -1.0 [-2.6, 0.5] | 32.3 [32.0, 32.5] | 14.9* [14.2, 15.7] | 0.62 [0.60, 0.63] | 0.59 [0.58, 0.59] | 0.63* [0.62, 0.64] |
|  | Framingham (blood) | Cancer | 464 (16.3%) | 16.3 [15.0, 17.7] | -1.0 [-2.6, 0.5] | 27.3 [26.8, 27.7] | 11.0* [9.5, 12.4] | 0.61 [0.58, 0.63] | 0.59 [0.57, 0.61] | 0.67 [0.63, 0.70] |
|  |  | Controls | 1,974 (17.3%) | 17.3 [16.7, 18.0] | -1.0 [-2.6, 0.5] | 27.9 [27.7, 28.1] | 10.5* [9.8, 11.3] | 0.60 [0.58, 0.61] | 0.57 [0.56, 0.57] | 0.65 [0.64, 0.66] |
|  | SCORE2 / | Cancer | 464 (16.3%) | 16.3 [15.0, 17.7] | -1.0 [-2.6, 0.5] | 8.3 [8.2, 8.4] | -8.0* [-9.4, -6.6] | 0.61 [0.58, 0.63] | 0.57 [0.55, 0.59] | 0.69 [0.66, 0.72] |
|  | SCORE-OP | Controls | 1,974 (17.3%) | 17.3 [16.7, 18.0] | -1.0 [-2.6, 0.5] | 8.5 [8.4, 8.5] | -8.9* [-9.6, -8.2] | 0.60 [0.59, 0.62] | 0.58 [0.57, 0.58] | 0.71 [0.70, 0.72] |
| **Atrial** | CHARGE-AF | Cancer | 234 (8.2%) | 8.2 [7.3, 9.3] | -0.6 [-1.8, 0.6] | 3.5 [3.4, 3.5] | -4.8* [-5.8, -3.7] | 0.62 [0.58, 0.65] | 0.59 [0.56, 0.61] | 0.66 [0.61, 0.71] |
| **fibrillation** |  | Controls | 1,007 (8.8%) | 8.8 [8.3, 9.4] | -0.6 [-1.8, 0.6] | 3.5 [3.5, 3.6] | -5.3* [-5.8, -4.8] | 0.64 [0.63, 0.66] | 0.61 [0.60, 0.61] | 0.70 [0.68, 0.71] |
| **Heart** | PCP-HF | Cancer | 97 (3.4%) | 3.4 [2.8, 4.1] | 0.4 [-0.4, 1.1] | 6.3 [6.1, 6.4] | 2.8* [2.1, 3.6] | 0.63 [0.58, 0.68] | 0.55* [0.50, 0.59] | 0.36* [0.27, 0.45] |
| **failure** |  | Controls | 348 (3.1%) | 3.1 [2.8, 3.4] | 0.4 [-0.4, 1.1] | 6.5 [6.4, 6.6] | 3.4* [3.1, 3.8] | 0.65 [0.62, 0.68] | 0.61* [0.60, 0.62] | 0.50* [0.48, 0.52] |
| **Stroke** | QStroke | Cancer | 84 (3.0%) | 3.0 [2.4, 3.6] | 0.3 [-0.4, 1.1] | 7.0 [6.9, 7.1] | 4.0* [3.3, 4.7] | 0.63 [0.57, 0.69] | 0.61 [0.57, 0.66] | 0.67 [0.57, 0.76] |
|  |  | Controls | 300 (2.6%) | 2.6 [2.4, 2.9] | 0.3 [-0.4, 1.1] | 7.1 [7.1, 7.2] | 4.5* [4.2, 4.8] | 0.64 [0.60, 0.67] | 0.60 [0.59, 0.62] | 0.67 [0.65, 0.69] |
|  | CHADsVASc | Cancer | 84 (3.0%) | 3.0 [2.4, 3.6] | 0.3 [-0.4, 1.1] | 1.1 [1.1, 1.1] |  | 0.58 [0.53, 0.64] | 0.56 [0.50, 0.61] | 0.39 [0.29, 0.49] |
|  |  | Controls | 300 (2.6%) | 2.6 [2.4, 2.9] | 0.3 [-0.4, 1.1] | 1.1 [1.1, 1.1] |  | 0.61 [0.58, 0.64] | 0.58 [0.57, 0.59] | 0.47 [0.44, 0.49] |

**Supplemental Table 10 footnote:** Table shows the event counts, ten-year cumulative incidence, average risk score and predictive performance statistics. Risk score means are given for each outcome-cancer-risk-score combination. Cumulative incidence and 95% confidence intervals are from Fine-Gray models accounting for the competing risk of death at ten years follow-up. The column marked in yellow indicates calibration differences between the risk score and the observed incidence, where the asterisk (*) indicates where this difference is significantly different from zero. All other asterisks indicate significant differences between cancer cases and controls. The final three columns reflect performance scoring of the candidate risk scores in cancer and control groups. Area under the receiver operating curve (AUC) is calculated from time-dependent analyses adjusted by the competing risk of death. Balanced accuracy and sensitivity are binary metrics calculated using ten-year outcomes, with confidence intervals derived from bootstrapping and permutation testing with 1000 replicates. Calibration is not possible for CHADsVASc as this is an ordinal scale. CVD 1 = combined endpoint including non-fatal myocardial infarction, non-fatal stroke or cardiovascular mortality, where cardiovascular mortality is defined as any death with a primary cause from ICD10 I00-I80. CVD 2 = combined endpoint including everything from CVD 1 plus incident atrial fibrillation, heart failure, non-ischaemic cardiomyopathies and valvular heart disease.

## Supplemental Table 11: Brain/CNS cancer

| **Outcome** | **Index** | **Group** | **Events within 10 years** | **Cumulative incidence** | **Difference in incidence between cancer and controls** | **Average risk score** | **Difference between observed incidence and risk score** | **AUC** | **Balanced accuracy** | **Sensitivity** |
| --- | --- | --- | --- | --- | --- | --- | --- | --- | --- | --- |
| **CVD 1** | QRISK3 | Cancer | 25 (9.8%) | 9.8* [6.6, 13.9] | 6.4* [2.2, 10.6] | 9.9 [8.9, 11.0] | 0.1 [-4.1, 4.3] | 0.59* [0.48, 0.70] | 0.55* [0.43, 0.67] | 0.52* [0.29, 0.75] |
|  |  | Controls | 35 (3.4%) | 3.4* [2.4, 4.7] | 6.4* [2.2, 10.6] | 9.9 [9.3, 10.4] | 6.4* [5.1, 7.8] | 0.77* [0.71, 0.83] | 0.75* [0.72, 0.78] | 0.89* [0.83, 0.94] |
|  | Framingham (BMI) | Cancer | 25 (9.8%) | 9.8* [6.6, 13.9] | 6.4* [2.2, 10.6] | 15.1 [13.7, 16.6] | 5.3* [1.0, 9.6] | 0.65 [0.54, 0.76] | 0.58* [0.46, 0.70] | 0.60* [0.37, 0.83] |
|  |  | Controls | 35 (3.4%) | 3.4* [2.4, 4.7] | 6.4* [2.2, 10.6] | 15.3 [14.6, 16.1] | 11.9* [10.4, 13.4] | 0.78 [0.72, 0.85] | 0.74* [0.71, 0.77] | 0.89* [0.83, 0.94] |
|  | Framingham (blood) | Cancer | 25 (9.8%) | 9.8* [6.6, 13.9] | 6.4* [2.2, 10.6] | 13.6 [12.3, 14.9] | 3.8 [-0.5, 8.0] | 0.65 [0.54, 0.76] | 0.59* [0.49, 0.68] | 0.72* [0.53, 0.91] |
|  |  | Controls | 35 (3.4%) | 3.4* [2.4, 4.7] | 6.4* [2.2, 10.6] | 13.7 [13.0, 14.3] | 10.2* [8.8, 11.6] | 0.79 [0.73, 0.86] | 0.73* [0.71, 0.75] | 0.97* [0.93, 1.00] |
|  | SCORE2 / | Cancer | 25 (9.8%) | 9.8* [6.6, 13.9] | 6.4* [2.2, 10.6] | 4.4 [4.1, 4.8] | -5.4* [-9.5, -1.4] | 0.67 [0.55, 0.78] | 0.61 [0.48, 0.74] | 0.36 [0.10, 0.62] |
|  | SCORE-OP | Controls | 35 (3.4%) | 3.4* [2.4, 4.7] | 6.4* [2.2, 10.6] | 4.5 [4.3, 4.7] | 1.1 [-0.2, 2.3] | 0.78 [0.72, 0.85] | 0.73 [0.70, 0.76] | 0.63 [0.57, 0.69] |
| **CVD 2** | QRISK3 | Cancer | 35 (13.8%) | 13.8* [9.9, 18.3] | 7.1* [2.2, 11.9] | 9.9 [8.9, 11.0] | -3.8 [-8.5, 0.8] | 0.62 [0.52, 0.72] | 0.60* [0.51, 0.69] | 0.63 [0.46, 0.80] |
|  |  | Controls | 68 (6.7%) | 6.7* [5.3, 8.3] | 7.1* [2.2, 11.9] | 9.9 [9.3, 10.4] | 3.2* [1.4, 4.9] | 0.76 [0.72, 0.81] | 0.73* [0.70, 0.75] | 0.84 [0.80, 0.88] |
|  | Framingham (BMI) | Cancer | 35 (13.8%) | 13.8* [9.9, 18.3] | 7.1* [2.2, 11.9] | 15.1 [13.7, 16.6] | 1.3 [-3.4, 6.1] | 0.66 [0.55, 0.76] | 0.60 [0.51, 0.69] | 0.63 [0.45, 0.80] |
|  |  | Controls | 68 (6.7%) | 6.7* [5.3, 8.3] | 7.1* [2.2, 11.9] | 15.3 [14.6, 16.1] | 8.6* [6.8, 10.5] | 0.75 [0.70, 0.81] | 0.71 [0.68, 0.73] | 0.81 [0.77, 0.85] |
|  | Framingham (blood) | Cancer | 35 (13.8%) | 13.8* [9.9, 18.3] | 7.1* [2.2, 11.9] | 13.6 [12.3, 14.9] | -0.2 [-4.9, 4.5] | 0.66 [0.55, 0.76] | 0.59* [0.51, 0.66] | 0.71* [0.57, 0.86] |
|  |  | Controls | 68 (6.7%) | 6.7* [5.3, 8.3] | 7.1* [2.2, 11.9] | 13.7 [13.0, 14.3] | 7.0* [5.2, 8.7] | 0.75 [0.69, 0.80] | 0.70* [0.69, 0.72] | 0.91* [0.88, 0.95] |
|  | SCORE2 / | Cancer | 35 (13.8%) | 13.8* [9.9, 18.3] | 7.1* [2.2, 11.9] | 4.4 [4.1, 4.8] | -9.4* [-13.9, -4.8] | 0.68 [0.57, 0.78] | 0.63 [0.55, 0.72] | 0.66 [0.49, 0.83] |
|  | SCORE-OP | Controls | 68 (6.7%) | 6.7* [5.3, 8.3] | 7.1* [2.2, 11.9] | 4.5 [4.3, 4.7] | -2.2* [-3.8, -0.5] | 0.75 [0.70, 0.80] | 0.71 [0.69, 0.73] | 0.79 [0.75, 0.84] |
| **Atrial** | CHARGE-AF | Cancer | 13 (5.1%) | 5.1 [2.9, 8.3] | 2.9 [-0.5, 6.2] | 1.4 [1.2, 1.6] | -3.7* [-6.9, -0.5] | 0.79 [0.66, 0.92] | 0.71 [0.56, 0.86] | 0.69 [0.39, 0.99] |
| **fibrillation** |  | Controls | 23 (2.3%) | 2.3 [1.5, 3.3] | 2.9 [-0.5, 6.2] | 1.5 [1.4, 1.6] | -0.8 [-1.9, 0.3] | 0.76 [0.68, 0.84] | 0.74 [0.70, 0.77] | 0.78 [0.71, 0.85] |
| **Heart** | PCP-HF | Cancer | 2 (0.8%) | 0.8 [0.2, 2.6] | -0.4 [-2.4, 1.6] | 2.5 [2.1, 2.9] | 1.8 [-0.1, 3.6] | 0.92 [0.82, 1.00] | 0.69 [0.46, 0.92] | 0.50 [0.05, 0.95] |
| **failure** |  | Controls | 12 (1.2%) | 1.2 [0.6, 2.0] | -0.4 [-2.4, 1.6] | 2.5 [2.3, 2.7] | 1.3* [0.5, 2.2] | 0.78 [0.63, 0.92] | 0.78 [0.70, 0.85] | 0.67 [0.52, 0.81] |
| **Stroke** | QStroke | Cancer | 19 (7.5%) | 7.5* [4.7, 11.2] | 6.8* [3.1, 10.5] | 3.3 [3.0, 3.6] | -4.2* [-7.9, -0.5] | 0.55* [0.42, 0.68] | 0.49* [0.30, 0.68] | 0.37* [0.00, 0.76] |
|  |  | Controls | 7 (0.7%) | 0.7* [0.3, 1.4] | 6.8* [3.1, 10.5] | 3.4 [3.2, 3.6] | 2.7* [2.0, 3.4] | 0.86* [0.77, 0.96] | 0.81* [0.76, 0.86] | 1.00* [0.90, 1.00] |
|  | CHADsVASc | Cancer | 19 (7.5%) | 7.5* [4.7, 11.2] | 6.8* [3.1, 10.5] | 1.2 [1.1, 1.3] |  | 0.52* [0.40, 0.64] | 0.50* [0.32, 0.69] | 0.32* [0.00, 0.68] |
|  |  | Controls | 7 (0.7%) | 0.7* [0.3, 1.4] | 6.8* [3.1, 10.5] | 1.1 [1.1, 1.2] |  | 0.84* [0.72, 0.95] | 0.80* [0.74, 0.85] | 0.86* [0.75, 0.96] |

**Supplemental Table 11 footnote:** Table shows the event counts, ten-year cumulative incidence, average risk score and predictive performance statistics. Risk score means are given for each outcome-cancer-risk-score combination. Cumulative incidence and 95% confidence intervals are from Fine-Gray models accounting for the competing risk of death at ten years follow-up. The column marked in yellow indicates calibration differences between the risk score and the observed incidence, where the asterisk (*) indicates where this difference is significantly different from zero. All other asterisks indicate significant differences between cancer cases and controls. The final three columns reflect performance scoring of the candidate risk scores in cancer and control groups. Area under the receiver operating curve (AUC) is calculated from time-dependent analyses adjusted by the competing risk of death. Balanced accuracy and sensitivity are binary metrics calculated using ten-year outcomes, with confidence intervals derived from bootstrapping and permutation testing with 1000 replicates. Calibration is not possible for CHADsVASc as this is an ordinal scale. CVD 1 = combined endpoint including non-fatal myocardial infarction, non-fatal stroke or cardiovascular mortality, where cardiovascular mortality is defined as any death with a primary cause from ICD10 I00-I80. CVD 2 = combined endpoint including everything from CVD 1 plus incident atrial fibrillation, heart failure, non-ischaemic cardiomyopathies and valvular heart disease.

## Supplemental Table 12: Hematological cancer

| **Outcome** | **Index** | **Group** | **Events within 10 years** | **Cumulative incidence** | **Difference in incidence between cancer and controls** | **Average risk score** | **Difference between observed incidence and risk score** | **AUC** | **Balanced accuracy** | **Sensitivity** |
| --- | --- | --- | --- | --- | --- | --- | --- | --- | --- | --- |
| **CVD 1** | QRISK3 | Cancer | 227 (8.9%) | 8.9* [7.9, 10.1] | 4.0* [2.7, 5.2] | 12.9 [12.6, 13.3] | 4.0* [2.8, 5.2] | 0.68 [0.64, 0.71] | 0.63 [0.59, 0.66] | 0.60* [0.54, 0.67] |
|  |  | Controls | 507 (5.0%) | 5.0* [4.6, 5.4] | 4.0* [2.7, 5.2] | 13.2 [13.0, 13.4] | 8.2* [7.7, 8.7] | 0.72 [0.70, 0.74] | 0.67 [0.66, 0.68] | 0.70* [0.69, 0.72] |
|  | Framingham (BMI) | Cancer | 227 (8.9%) | 8.9* [7.9, 10.1] | 4.0* [2.7, 5.2] | 19.1 [18.5, 19.6] | 10.1* [8.8, 11.4] | 0.67 [0.64, 0.71] | 0.63 [0.60, 0.66] | 0.71* [0.65, 0.77] |
|  |  | Controls | 507 (5.0%) | 5.0* [4.6, 5.4] | 4.0* [2.7, 5.2] | 19.9 [19.6, 20.2] | 14.9* [14.4, 15.4] | 0.70 [0.68, 0.72] | 0.66 [0.65, 0.66] | 0.80* [0.78, 0.81] |
|  | Framingham (blood) | Cancer | 227 (8.9%) | 8.9* [7.9, 10.1] | 4.0* [2.7, 5.2] | 16.9 [16.4, 17.4] | 8.0* [6.7, 9.2] | 0.68 [0.65, 0.72] | 0.63 [0.59, 0.66] | 0.76 [0.70, 0.82] |
|  |  | Controls | 507 (5.0%) | 5.0* [4.6, 5.4] | 4.0* [2.7, 5.2] | 17.5 [17.3, 17.8] | 12.5* [12.0, 13.0] | 0.70 [0.68, 0.72] | 0.65 [0.64, 0.65] | 0.83 [0.81, 0.84] |
|  | SCORE2 / | Cancer | 227 (8.9%) | 8.9* [7.9, 10.1] | 4.0* [2.7, 5.2] | 5.5 [5.4, 5.6] | -3.5* [-4.6, -2.3] | 0.68 [0.64, 0.71] | 0.63 [0.59, 0.66] | 0.70 [0.63, 0.76] |
|  | SCORE-OP | Controls | 507 (5.0%) | 5.0* [4.6, 5.4] | 4.0* [2.7, 5.2] | 5.6 [5.6, 5.7] | 0.6* [0.2, 1.1] | 0.69 [0.67, 0.72] | 0.64 [0.63, 0.65] | 0.75 [0.73, 0.76] |
| **CVD 2** | QRISK3 | Cancer | 533 (21.0%) | 21.0* [19.4, 22.6] | 10.6* [8.9, 12.3] | 12.9 [12.6, 13.3] | -8.1* [-9.7, -6.4] | 0.65* [0.62, 0.67] | 0.61* [0.58, 0.63] | 0.56* [0.51, 0.61] |
|  |  | Controls | 1,058 (10.4%) | 10.4* [9.8, 11.0] | 10.6* [8.9, 12.3] | 13.2 [13.0, 13.4] | 2.8* [2.1, 3.4] | 0.72* [0.70, 0.73] | 0.67* [0.66, 0.67] | 0.71* [0.69, 0.72] |
|  | Framingham (BMI) | Cancer | 533 (21.0%) | 21.0* [19.4, 22.6] | 10.6* [8.9, 12.3] | 19.1 [18.5, 19.6] | -1.9* [-3.6, -0.2] | 0.64* [0.61, 0.67] | 0.60* [0.58, 0.63] | 0.53* [0.48, 0.58] |
|  |  | Controls | 1,058 (10.4%) | 10.4* [9.8, 11.0] | 10.6* [8.9, 12.3] | 19.9 [19.6, 20.2] | 9.5* [8.8, 10.1] | 0.71* [0.70, 0.73] | 0.66* [0.65, 0.67] | 0.67* [0.66, 0.69] |
|  | Framingham (blood) | Cancer | 533 (21.0%) | 21.0* [19.4, 22.6] | 10.6* [8.9, 12.3] | 16.9 [16.4, 17.4] | -4.1* [-5.8, -2.4] | 0.64* [0.61, 0.66] | 0.61* [0.59, 0.64] | 0.59* [0.54, 0.64] |
|  |  | Controls | 1,058 (10.4%) | 10.4* [9.8, 11.0] | 10.6* [8.9, 12.3] | 17.5 [17.3, 17.8] | 7.1* [6.5, 7.8] | 0.69* [0.68, 0.71] | 0.64* [0.64, 0.65] | 0.69* [0.68, 0.70] |
|  | SCORE2 / | Cancer | 533 (21.0%) | 21.0* [19.4, 22.6] | 10.6* [8.9, 12.3] | 5.5 [5.4, 5.6] | -15.5* [-17.1, -13.9] | 0.64* [0.61, 0.66] | 0.60* [0.57, 0.62] | 0.53* [0.48, 0.58] |
|  | SCORE-OP | Controls | 1,058 (10.4%) | 10.4* [9.8, 11.0] | 10.6* [8.9, 12.3] | 5.6 [5.6, 5.7] | -4.8* [-5.4, -4.2] | 0.70* [0.69, 0.72] | 0.65* [0.64, 0.66] | 0.67* [0.66, 0.68] |
| **Atrial** | CHARGE-AF | Cancer | 230 (9.1%) | 9.1* [8.0, 10.2] | 4.1* [2.9, 5.4] | 2.0 [1.9, 2.1] | -7.1* [-8.2, -5.9] | 0.68* [0.64, 0.71] | 0.62* [0.59, 0.66] | 0.53* [0.46, 0.60] |
| **fibrillation** |  | Controls | 500 (4.9%) | 4.9* [4.5, 5.4] | 4.1* [2.9, 5.4] | 2.1 [2.0, 2.1] | -2.9* [-3.3, -2.4] | 0.75* [0.73, 0.77] | 0.69* [0.68, 0.70] | 0.69* [0.67, 0.70] |
| **Heart** | PCP-HF | Cancer | 165 (6.5%) | 6.5* [5.6, 7.5] | 4.4* [3.3, 5.4] | 3.6 [3.4, 3.8] | -2.9* [-3.9, -1.9] | 0.64* [0.59, 0.68] | 0.59* [0.54, 0.64] | 0.47* [0.36, 0.57] |
| **failure** |  | Controls | 217 (2.1%) | 2.1* [1.9, 2.4] | 4.4* [3.3, 5.4] | 3.7 [3.7, 3.8] | 1.6* [1.3, 1.9] | 0.77* [0.74, 0.80] | 0.71* [0.69, 0.72] | 0.72* [0.69, 0.74] |
| **Stroke** | QStroke | Cancer | 86 (3.4%) | 3.4* [2.7, 4.1] | 1.6* [0.8, 2.4] | 4.5 [4.4, 4.6] | 1.1* [0.3, 1.9] | 0.64 [0.58, 0.69] | 0.57* [0.51, 0.64] | 0.47* [0.34, 0.59] |
|  |  | Controls | 180 (1.8%) | 1.8* [1.5, 2.0] | 1.6* [0.8, 2.4] | 4.6 [4.5, 4.7] | 2.8* [2.5, 3.1] | 0.71 [0.67, 0.74] | 0.67* [0.65, 0.68] | 0.67* [0.64, 0.70] |
|  | CHADsVASc | Cancer | 86 (3.4%) | 3.4* [2.7, 4.1] | 1.6* [0.8, 2.4] | 1.3 [1.3, 1.3] |  | 0.58 [0.52, 0.64] | 0.56 [0.50, 0.62] | 0.48 [0.36, 0.59] |
|  |  | Controls | 180 (1.8%) | 1.8* [1.5, 2.0] | 1.6* [0.8, 2.4] | 1.3 [1.2, 1.3] |  | 0.61 [0.57, 0.65] | 0.60 [0.59, 0.62] | 0.54 [0.52, 0.57] |

**Supplemental Table 12 footnote:** Table shows the event counts, ten-year cumulative incidence, average risk score and predictive performance statistics. Risk score means are given for each outcome-cancer-risk-score combination. Cumulative incidence and 95% confidence intervals are from Fine-Gray models accounting for the competing risk of death at ten years follow-up. The column marked in yellow indicates calibration differences between the risk score and the observed incidence, where the asterisk (*) indicates where this difference is significantly different from zero. All other asterisks indicate significant differences between cancer cases and controls. The final three columns reflect performance scoring of the candidate risk scores in cancer and control groups. Area under the receiver operating curve (AUC) is calculated from time-dependent analyses adjusted by the competing risk of death. Balanced accuracy and sensitivity are binary metrics calculated using ten-year outcomes, with confidence intervals derived from bootstrapping and permutation testing with 1000 replicates. Calibration is not possible for CHADsVASc as this is an ordinal scale. CVD 1 = combined endpoint including non-fatal myocardial infarction, non-fatal stroke or cardiovascular mortality, where cardiovascular mortality is defined as any death with a primary cause from ICD10 I00-I80. CVD 2 = combined endpoint including everything from CVD 1 plus incident atrial fibrillation, heart failure, non-ischaemic cardiomyopathies and valvular heart disease.

## Supplemental Table 13: Non-Hodgkin’s lymphoma

| **Outcome** | **Index** | **Group** | **Events within 10 years** | **Cumulative incidence** | **Difference in incidence between cancer and controls** | **Average risk score** | **Difference between observed incidence and risk score** | **AUC** | **Balanced accuracy** | **Sensitivity** |
| --- | --- | --- | --- | --- | --- | --- | --- | --- | --- | --- |
| **CVD 1** | QRISK3 | Cancer | 55 (6.2%) | 6.2 [4.8, 7.9] | 1.0 [-0.9, 2.9] | 12.5 [11.9, 13.1] | 6.3* [4.5, 8.1] | 0.69 [0.62, 0.77] | 0.66 [0.60, 0.72] | 0.73 [0.62, 0.84] |
|  |  | Controls | 185 (5.2%) | 5.2 [4.5, 6.0] | 1.0 [-0.9, 2.9] | 12.9 [12.6, 13.2] | 7.6* [6.8, 8.4] | 0.72 [0.69, 0.75] | 0.68 [0.66, 0.69] | 0.78 [0.76, 0.81] |
|  | Framingham (BMI) | Cancer | 55 (6.2%) | 6.2 [4.8, 7.9] | 1.0 [-0.9, 2.9] | 18.4 [17.6, 19.3] | 12.2* [10.3, 14.1] | 0.69 [0.61, 0.77] | 0.64 [0.57, 0.70] | 0.60 [0.48, 0.72] |
|  |  | Controls | 185 (5.2%) | 5.2 [4.5, 6.0] | 1.0 [-0.9, 2.9] | 19.6 [19.1, 20.0] | 14.4* [13.5, 15.2] | 0.71 [0.67, 0.74] | 0.66 [0.65, 0.68] | 0.69 [0.66, 0.72] |
|  | Framingham (blood) | Cancer | 55 (6.2%) | 6.2 [4.8, 7.9] | 1.0 [-0.9, 2.9] | 16.4 [15.6, 17.1] | 10.1* [8.2, 12.0] | 0.70 [0.62, 0.77] | 0.66 [0.60, 0.72] | 0.64 [0.52, 0.76] |
|  |  | Controls | 185 (5.2%) | 5.2 [4.5, 6.0] | 1.0 [-0.9, 2.9] | 17.1 [16.7, 17.5] | 11.9* [11.0, 12.7] | 0.69 [0.65, 0.73] | 0.65 [0.63, 0.66] | 0.66 [0.63, 0.69] |
|  | SCORE2 / | Cancer | 55 (6.2%) | 6.2 [4.8, 7.9] | 1.0 [-0.9, 2.9] | 5.4 [5.2, 5.6] | -0.8 [-2.6, 0.9] | 0.68 [0.60, 0.76] | 0.61 [0.56, 0.66] | 0.73 [0.62, 0.83] |
|  | SCORE-OP | Controls | 185 (5.2%) | 5.2 [4.5, 6.0] | 1.0 [-0.9, 2.9] | 5.6 [5.5, 5.7] | 0.3 [-0.4, 1.1] | 0.69 [0.66, 0.73] | 0.64 [0.63, 0.65] | 0.81 [0.78, 0.83] |
| **CVD 2** | QRISK3 | Cancer | 145 (16.4%) | 16.4* [14.0, 18.9] | 5.7* [3.0, 8.4] | 12.5 [11.9, 13.1] | -3.9* [-6.5, -1.3] | 0.60* [0.55, 0.65] | 0.58* [0.54, 0.63] | 0.57* [0.49, 0.65] |
|  |  | Controls | 378 (10.7%) | 10.7* [9.7, 11.7] | 5.7* [3.0, 8.4] | 12.9 [12.6, 13.2] | 2.2* [1.1, 3.3] | 0.70* [0.68, 0.73] | 0.66* [0.65, 0.67] | 0.74* [0.72, 0.76] |
|  | Framingham (BMI) | Cancer | 145 (16.4%) | 16.4* [14.0, 18.9] | 5.7* [3.0, 8.4] | 18.4 [17.6, 19.3] | 2.0 [-0.6, 4.7] | 0.61* [0.56, 0.66] | 0.59* [0.54, 0.63] | 0.49* [0.40, 0.58] |
|  |  | Controls | 378 (10.7%) | 10.7* [9.7, 11.7] | 5.7* [3.0, 8.4] | 19.6 [19.1, 20.0] | 8.9* [7.8, 10.0] | 0.70* [0.67, 0.72] | 0.66* [0.64, 0.67] | 0.66* [0.64, 0.68] |
|  | Framingham (blood) | Cancer | 145 (16.4%) | 16.4* [14.0, 18.9] | 5.7* [3.0, 8.4] | 16.4 [15.6, 17.1] | -0.1 [-2.7, 2.6] | 0.59* [0.54, 0.64] | 0.59 [0.54, 0.63] | 0.48* [0.40, 0.57] |
|  |  | Controls | 378 (10.7%) | 10.7* [9.7, 11.7] | 5.7* [3.0, 8.4] | 17.1 [16.7, 17.5] | 6.4* [5.3, 7.5] | 0.67* [0.65, 0.70] | 0.63 [0.62, 0.64] | 0.61* [0.59, 0.64] |
|  | SCORE2 / | Cancer | 145 (16.4%) | 16.4* [14.0, 18.9] | 5.7* [3.0, 8.4] | 5.4 [5.2, 5.6] | -11.0* [-13.5, -8.5] | 0.59* [0.54, 0.64] | 0.58* [0.53, 0.62] | 0.52* [0.44, 0.61] |
|  | SCORE-OP | Controls | 378 (10.7%) | 10.7* [9.7, 11.7] | 5.7* [3.0, 8.4] | 5.6 [5.5, 5.7] | -5.1* [-6.2, -4.1] | 0.69* [0.66, 0.71] | 0.64* [0.63, 0.66] | 0.67* [0.65, 0.69] |
| **Atrial** | CHARGE-AF | Cancer | 65 (7.4%) | 7.4* [5.8, 9.2] | 2.1* [0.1, 4.1] | 2.0 [1.9, 2.1] | -5.4* [-7.2, -3.6] | 0.65 [0.58, 0.72] | 0.61 [0.56, 0.67] | 0.66 [0.55, 0.77] |
| **fibrillation** |  | Controls | 186 (5.3%) | 5.3* [4.6, 6.0] | 2.1* [0.1, 4.1] | 2.0 [2.0, 2.1] | -3.2* [-4.0, -2.5] | 0.71 [0.67, 0.74] | 0.66 [0.65, 0.68] | 0.77 [0.75, 0.80] |
| **Heart** | PCP-HF | Cancer | 50 (5.7%) | 5.7* [4.3, 7.3] | 3.7* [1.9, 5.4] | 3.4 [3.2, 3.6] | -2.3* [-3.9, -0.6] | 0.64* [0.57, 0.72] | 0.60* [0.51, 0.70] | 0.48* [0.30, 0.66] |
| **failure** |  | Controls | 70 (2.0%) | 2.0* [1.6, 2.5] | 3.7* [1.9, 5.4] | 3.6 [3.5, 3.8] | 1.7* [1.1, 2.2] | 0.77* [0.72, 0.82] | 0.72* [0.69, 0.74] | 0.74* [0.70, 0.79] |
| **Stroke** | QStroke | Cancer | 20 (2.3%) | 2.3 [1.4, 3.4] | 0.3 [-0.9, 1.6] | 4.4 [4.2, 4.6] | 2.1* [0.9, 3.3] | 0.67 [0.54, 0.80] | 0.60 [0.50, 0.70] | 0.50 [0.30, 0.70] |
|  |  | Controls | 69 (2.0%) | 2.0 [1.5, 2.4] | 0.3 [-0.9, 1.6] | 4.5 [4.4, 4.6] | 2.6* [2.1, 3.1] | 0.69 [0.62, 0.75] | 0.67 [0.64, 0.70] | 0.67 [0.62, 0.72] |
|  | CHADsVASc | Cancer | 20 (2.3%) | 2.3 [1.4, 3.4] | 0.3 [-0.9, 1.6] | 1.3 [1.2, 1.3] |  | 0.70 [0.58, 0.83] | 0.63 [0.52, 0.75] | 0.60 [0.38, 0.82] |
|  |  | Controls | 69 (2.0%) | 2.0 [1.5, 2.4] | 0.3 [-0.9, 1.6] | 1.3 [1.2, 1.3] |  | 0.61 [0.54, 0.68] | 0.60 [0.57, 0.63] | 0.54 [0.48, 0.59] |
|  |  |  |  |  |  |  |  |  |  |  |

**Supplemental Table 13 footnote:** Table shows the event counts, ten-year cumulative incidence, average risk score and predictive performance statistics. Risk score means are given for each outcome-cancer-risk-score combination. Cumulative incidence and 95% confidence intervals are from Fine-Gray models accounting for the competing risk of death at ten years follow-up. The column marked in yellow indicates calibration differences between the risk score and the observed incidence, where the asterisk (*) indicates where this difference is significantly different from zero. All other asterisks indicate significant differences between cancer cases and controls. The final three columns reflect performance scoring of the candidate risk scores in cancer and control groups. Area under the receiver operating curve (AUC) is calculated from time-dependent analyses adjusted by the competing risk of death. Balanced accuracy and sensitivity are binary metrics calculated using ten-year outcomes, with confidence intervals derived from bootstrapping and permutation testing with 1000 replicates. Calibration is not possible for CHADsVASc as this is an ordinal scale. CVD 1 = combined endpoint including non-fatal myocardial infarction, non-fatal stroke or cardiovascular mortality, where cardiovascular mortality is defined as any death with a primary cause from ICD10 I00-I80. CVD 2 = combined endpoint including everything from CVD 1 plus incident atrial fibrillation, heart failure, non-ischaemic cardiomyopathies and valvular heart disease.

## Supplemental Table 14: Hodgkin’s lymphoma

| **Outcome** | **Index** | **Group** | **Events within 10 years** | **Cumulative incidence** | **Difference in incidence between cancer and controls** | **Average risk score** | **Difference between observed incidence and risk score** | **AUC** | **Balanced accuracy** | **Sensitivity** |
| --- | --- | --- | --- | --- | --- | --- | --- | --- | --- | --- |
| **CVD 1** | QRISK3 | Cancer | 45 (12.0%) | 12.0* [9.0, 15.6] | 7.5* [3.8, 11.2] | 9.1 [8.3, 10.0] | -2.9 [-6.5, 0.7] | 0.73 [0.67, 0.80] | 0.66 [0.57, 0.74] | 0.60* [0.43, 0.77] |
|  |  | Controls | 68 (4.5%) | 4.5* [3.6, 5.7] | 7.5* [3.8, 11.2] | 9.3 [8.9, 9.8] | 4.8* [3.6, 6.0] | 0.79 [0.74, 0.83] | 0.74 [0.72, 0.76] | 0.81* [0.77, 0.85] |
|  | Framingham (BMI) | Cancer | 45 (12.0%) | 12.0* [9.0, 15.6] | 7.5* [3.8, 11.2] | 14.4 [13.2, 15.6] | 2.4 [-1.4, 6.1] | 0.72 [0.65, 0.79] | 0.69 [0.63, 0.75] | 0.84* [0.74, 0.95] |
|  |  | Controls | 68 (4.5%) | 4.5* [3.6, 5.7] | 7.5* [3.8, 11.2] | 14.6 [14.0, 15.2] | 10.1* [8.8, 11.4] | 0.78 [0.73, 0.82] | 0.73 [0.72, 0.75] | 0.97* [0.95, 1.00] |
|  | Framingham (blood) | Cancer | 45 (12.0%) | 12.0* [9.0, 15.6] | 7.5* [3.8, 11.2] | 13.3 [12.1, 14.4] | 1.2 [-2.5, 5.0] | 0.73 [0.66, 0.80] | 0.63 [0.54, 0.73] | 0.60 [0.43, 0.77] |
|  |  | Controls | 68 (4.5%) | 4.5* [3.6, 5.7] | 7.5* [3.8, 11.2] | 13.0 [12.5, 13.5] | 8.5* [7.2, 9.7] | 0.77 [0.72, 0.82] | 0.72 [0.70, 0.74] | 0.81 [0.77, 0.85] |
|  | SCORE2 / | Cancer | 45 (12.0%) | 12.0* [9.0, 15.6] | 7.5* [3.8, 11.2] | 4.3 [4.0, 4.6] | -7.8* [-11.3, -4.2] | 0.73 [0.66, 0.80] | 0.67 [0.59, 0.76] | 0.71 [0.55, 0.87] |
|  | SCORE-OP | Controls | 68 (4.5%) | 4.5* [3.6, 5.7] | 7.5* [3.8, 11.2] | 4.2 [4.1, 4.4] | -0.3 [-1.5, 0.8] | 0.77 [0.72, 0.82] | 0.72 [0.70, 0.74] | 0.82 [0.79, 0.86] |
| **CVD 2** | QRISK3 | Cancer | 89 (23.8%) | 23.8* [19.6, 28.2] | 16.5* [11.9, 21.2] | 9.1 [8.3, 10.0] | -14.7* [-19.2, -10.2] | 0.68 [0.62, 0.74] | 0.61* [0.54, 0.68] | 0.48* [0.35, 0.62] |
|  |  | Controls | 109 (7.3%) | 7.3* [6.0, 8.7] | 16.5* [11.9, 21.2] | 9.3 [8.9, 9.8] | 2.1* [0.6, 3.5] | 0.78 [0.73, 0.82] | 0.73* [0.71, 0.75] | 0.77* [0.74, 0.80] |
|  | Framingham (BMI) | Cancer | 89 (23.8%) | 23.8* [19.6, 28.2] | 16.5* [11.9, 21.2] | 14.4 [13.2, 15.6] | -9.4* [-14.0, -4.8] | 0.67* [0.61, 0.73] | 0.59* [0.52, 0.66] | 0.44* [0.30, 0.57] |
|  |  | Controls | 109 (7.3%) | 7.3* [6.0, 8.7] | 16.5* [11.9, 21.2] | 14.6 [14.0, 15.2] | 7.3* [5.8, 8.9] | 0.78* [0.74, 0.82] | 0.72* [0.70, 0.74] | 0.74* [0.71, 0.78] |
|  | Framingham (blood) | Cancer | 89 (23.8%) | 23.8* [19.6, 28.2] | 16.5* [11.9, 21.2] | 13.3 [12.1, 14.4] | -10.5* [-15.1, -6.0] | 0.67 [0.61, 0.73] | 0.60* [0.53, 0.67] | 0.52* [0.39, 0.64] |
|  |  | Controls | 109 (7.3%) | 7.3* [6.0, 8.7] | 16.5* [11.9, 21.2] | 13.0 [12.5, 13.5] | 5.7* [4.3, 7.2] | 0.76 [0.72, 0.81] | 0.72* [0.71, 0.74] | 0.80* [0.77, 0.83] |
|  | SCORE2 / | Cancer | 89 (23.8%) | 23.8* [19.6, 28.2] | 16.5* [11.9, 21.2] | 4.3 [4.0, 4.6] | -19.5* [-24.0, -15.1] | 0.66 [0.60, 0.73] | 0.61* [0.54, 0.68] | 0.57* [0.45, 0.70] |
|  | SCORE-OP | Controls | 109 (7.3%) | 7.3* [6.0, 8.7] | 16.5* [11.9, 21.2] | 4.2 [4.1, 4.4] | -3.1* [-4.5, -1.7] | 0.76 [0.72, 0.80] | 0.71* [0.69, 0.73] | 0.80* [0.77, 0.83] |
| **Atrial** | CHARGE-AF | Cancer | 29 (7.8%) | 7.8* [5.3, 10.8] | 4.9* [1.7, 8.0] | 1.4 [1.2, 1.5] | -6.4* [-9.4, -3.4] | 0.71* [0.62, 0.79] | 0.63* [0.51, 0.74] | 0.45* [0.22, 0.67] |
| **fibrillation** |  | Controls | 43 (2.9%) | 2.9* [2.1, 3.8] | 4.9* [1.7, 8.0] | 1.3 [1.3, 1.4] | -1.5* [-2.5, -0.6] | 0.85* [0.80, 0.90] | 0.78* [0.75, 0.81] | 0.77* [0.71, 0.82] |
| **Heart** | PCP-HF | Cancer | 25 (6.7%) | 6.7* [4.4, 9.5] | 5.3* [2.4, 8.3] | 2.2 [1.9, 2.5] | -4.5* [-7.3, -1.6] | 0.63 [0.55, 0.72] | 0.52* [0.37, 0.67] | 0.24* [0.00, 0.54] |
| **failure** |  | Controls | 20 (1.3%) | 1.3* [0.8, 2.0] | 5.3* [2.4, 8.3] | 2.4 [2.2, 2.5] | 1.0* [0.3, 1.8] | 0.80 [0.71, 0.90] | 0.78* [0.75, 0.82] | 0.80* [0.73, 0.87] |
| **Stroke** | QStroke | Cancer | 12 (3.2%) | 3.2 [1.8, 5.4] | 1.6 [-0.7, 3.9] | 3.1 [2.8, 3.3] | -0.1 [-2.3, 2.0] | 0.73 [0.62, 0.84] | 0.66 [0.52, 0.80] | 0.67 [0.38, 0.95] |
|  |  | Controls | 24 (1.6%) | 1.6 [1.1, 2.3] | 1.6 [-0.7, 3.9] | 3.1 [2.9, 3.2] | 1.5* [0.7, 2.2] | 0.77 [0.68, 0.85] | 0.73 [0.70, 0.77] | 0.83 [0.76, 0.90] |
|  | CHADsVASc | Cancer | 12 (3.2%) | 3.2 [1.8, 5.4] | 1.6 [-0.7, 3.9] | 1.1 [1.0, 1.2] |  | 0.52 [0.36, 0.69] | 0.57 [0.41, 0.74] | 0.42 [0.09, 0.75] |
|  |  | Controls | 24 (1.6%) | 1.6 [1.1, 2.3] | 1.6 [-0.7, 3.9] | 1.1 [1.0, 1.1] |  | 0.57 [0.43, 0.70] | 0.60 [0.56, 0.64] | 0.46 [0.38, 0.54] |

**Supplemental Table 14 footnote:** Table shows the event counts, ten-year cumulative incidence, average risk score and predictive performance statistics. Risk score means are given for each outcome-cancer-risk-score combination. Cumulative incidence and 95% confidence intervals are from Fine-Gray models accounting for the competing risk of death at ten years follow-up. The column marked in yellow indicates calibration differences between the risk score and the observed incidence, where the asterisk (*) indicates where this difference is significantly different from zero. All other asterisks indicate significant differences between cancer cases and controls. The final three columns reflect performance scoring of the candidate risk scores in cancer and control groups. Area under the receiver operating curve (AUC) is calculated from time-dependent analyses adjusted by the competing risk of death. Balanced accuracy and sensitivity are binary metrics calculated using ten-year outcomes, with confidence intervals derived from bootstrapping and permutation testing with 1000 replicates. Calibration is not possible for CHADsVASc as this is an ordinal scale. CVD 1 = combined endpoint including non-fatal myocardial infarction, non-fatal stroke or cardiovascular mortality, where cardiovascular mortality is defined as any death with a primary cause from ICD10 I00-I80. CVD 2 = combined endpoint including everything from CVD 1 plus incident atrial fibrillation, heart failure, non-ischaemic cardiomyopathies and valvular heart disease.

## Supplemental Table 15: Multiple myeloma

| **Outcome** | **Index** | **Group** | **Events within 10 years** | **Cumulative incidence** | **Difference in incidence between cancer and controls** | **Average risk score** | **Difference between observed incidence and risk score** | **AUC** | **Balanced accuracy** | **Sensitivity** |
| --- | --- | --- | --- | --- | --- | --- | --- | --- | --- | --- |
| **CVD 1** | QRISK3 | Cancer | 18 (11.0%) | 11.0 [6.8, 16.3] | 5.0 [-0.7, 10.7] | 14.9 [13.3, 16.4] | 3.9 [-1.7, 9.5] | 0.79 [0.68, 0.91] | 0.75 [0.61, 0.89] | 0.67 [0.40, 0.93] |
|  |  | Controls | 39 (5.9%) | 5.9 [4.3, 7.9] | 5.0 [-0.7, 10.7] | 15.3 [14.4, 16.1] | 9.3* [7.2, 11.5] | 0.68 [0.59, 0.77] | 0.66 [0.63, 0.69] | 0.54 [0.47, 0.60] |
|  | Framingham (BMI) | Cancer | 18 (11.0%) | 11.0 [6.8, 16.3] | 5.0 [-0.7, 10.7] | 20.7 [18.6, 22.8] | 9.7* [4.0, 15.5] | 0.73 [0.60, 0.85] | 0.67 [0.55, 0.78] | 0.78 [0.56, 1.00] |
|  |  | Controls | 39 (5.9%) | 5.9 [4.3, 7.9] | 5.0 [-0.7, 10.7] | 22.0 [20.9, 23.2] | 16.1* [13.8, 18.4] | 0.67 [0.59, 0.75] | 0.65 [0.62, 0.68] | 0.79 [0.74, 0.85] |
|  | Framingham (blood) | Cancer | 18 (11.0%) | 11.0 [6.8, 16.3] | 5.0 [-0.7, 10.7] | 18.4 [16.4, 20.3] | 7.4* [1.7, 13.1] | 0.72 [0.60, 0.85] | 0.69 [0.58, 0.80] | 0.83 [0.62, 1.00] |
|  |  | Controls | 39 (5.9%) | 5.9 [4.3, 7.9] | 5.0 [-0.7, 10.7] | 19.7 [18.7, 20.7] | 13.8* [11.5, 16.0] | 0.66 [0.58, 0.75] | 0.63 [0.60, 0.65] | 0.77 [0.72, 0.82] |
|  | SCORE2 / | Cancer | 18 (11.0%) | 11.0 [6.8, 16.3] | 5.0 [-0.7, 10.7] | 5.9 [5.4, 6.3] | -5.1 [-10.5, 0.3] | 0.72 [0.60, 0.84] | 0.66 [0.52, 0.80] | 0.67 [0.39, 0.94] |
|  | SCORE-OP | Controls | 39 (5.9%) | 5.9 [4.3, 7.9] | 5.0 [-0.7, 10.7] | 6.3 [6.0, 6.5] | 0.3 [-1.7, 2.3] | 0.64 [0.55, 0.73] | 0.62 [0.59, 0.66] | 0.64 [0.58, 0.71] |
| **CVD 2** | QRISK3 | Cancer | 36 (22.0%) | 22.0* [16.0, 28.6] | 10.2* [3.1, 17.3] | 14.9 [13.3, 16.4] | -7.1* [-13.9, -0.3] | 0.68 [0.57, 0.79] | 0.67 [0.57, 0.77] | 0.47 [0.28, 0.66] |
|  |  | Controls | 77 (11.7%) | 11.7* [9.4, 14.3] | 10.2* [3.1, 17.3] | 15.3 [14.4, 16.1] | 3.5* [0.8, 6.3] | 0.69 [0.63, 0.75] | 0.66 [0.64, 0.68] | 0.51 [0.46, 0.55] |
|  | Framingham (BMI) | Cancer | 36 (22.0%) | 22.0* [16.0, 28.6] | 10.2* [3.1, 17.3] | 20.7 [18.6, 22.8] | -1.2 [-8.2, 5.7] | 0.66 [0.55, 0.76] | 0.60 [0.50, 0.70] | 0.50 [0.32, 0.68] |
|  |  | Controls | 77 (11.7%) | 11.7* [9.4, 14.3] | 10.2* [3.1, 17.3] | 22.0 [20.9, 23.2] | 10.3* [7.5, 13.1] | 0.68 [0.62, 0.74] | 0.65 [0.63, 0.68] | 0.66 [0.62, 0.71] |
|  | Framingham (blood) | Cancer | 36 (22.0%) | 22.0* [16.0, 28.6] | 10.2* [3.1, 17.3] | 18.4 [16.4, 20.3] | -3.6 [-10.5, 3.3] | 0.67 [0.57, 0.78] | 0.63 [0.53, 0.73] | 0.56 [0.36, 0.75] |
|  |  | Controls | 77 (11.7%) | 11.7* [9.4, 14.3] | 10.2* [3.1, 17.3] | 19.7 [18.7, 20.7] | 8.0* [5.2, 10.8] | 0.66 [0.60, 0.72] | 0.64 [0.61, 0.66] | 0.65 [0.60, 0.70] |
|  | SCORE2 / | Cancer | 36 (22.0%) | 22.0* [16.0, 28.6] | 10.2* [3.1, 17.3] | 5.9 [5.4, 6.3] | -16.1* [-22.7, -9.4] | 0.67 [0.57, 0.77] | 0.61 [0.51, 0.71] | 0.56 [0.37, 0.74] |
|  | SCORE-OP | Controls | 77 (11.7%) | 11.7* [9.4, 14.3] | 10.2* [3.1, 17.3] | 6.3 [6.0, 6.5] | -5.5* [-8.1, -2.9] | 0.66 [0.60, 0.72] | 0.65 [0.63, 0.67] | 0.68 [0.63, 0.72] |
| **Atrial** | CHARGE-AF | Cancer | 16 (9.8%) | 9.8 [5.8, 14.9] | 4.9 [-0.6, 10.3] | 2.2 [1.9, 2.5] | -7.6* [-12.7, -2.4] | 0.63 [0.49, 0.76] | 0.59 [0.43, 0.74] | 0.38 [0.07, 0.68] |
| **fibrillation** |  | Controls | 32 (4.9%) | 4.9 [3.4, 6.7] | 4.9 [-0.6, 10.3] | 2.4 [2.3, 2.6] | -2.4* [-4.3, -0.6] | 0.75 [0.67, 0.83] | 0.71 [0.67, 0.75] | 0.69 [0.61, 0.76] |
| **Heart** | PCP-HF | Cancer | 12 (7.3%) | 7.3 [4.0, 12.0] | 4.1 [-0.8, 9.0] | 4.1 [3.5, 4.7] | -3.2 [-7.9, 1.5] | 0.70 [0.53, 0.86] | 0.62 [0.46, 0.77] | 0.58 [0.28, 0.89] |
| **failure** |  | Controls | 21 (3.2%) | 3.2 [2.0, 4.8] | 4.1 [-0.8, 9.0] | 4.4 [4.0, 4.7] | 1.2 [-0.4, 2.7] | 0.79 [0.70, 0.88] | 0.75 [0.71, 0.79] | 0.86 [0.78, 0.93] |
| **Stroke** | QStroke | Cancer | 8 (4.9%) | 4.9 [2.3, 9.0] | 3.0 [-1.2, 7.3] | 5.1 [4.6, 5.6] | 0.2 [-3.9, 4.3] | 0.74 [0.53, 0.95] | 0.74 [0.51, 0.96] | 0.62 [0.19, 1.00] |
|  |  | Controls | 12 (1.8%) | 1.8 [1.0, 3.1] | 3.0 [-1.2, 7.3] | 5.3 [5.0, 5.6] | 3.5* [2.2, 4.8] | 0.66 [0.51, 0.82] | 0.66 [0.60, 0.72] | 0.50 [0.38, 0.62] |
|  | CHADsVASc | Cancer | 8 (4.9%) | 4.9 [2.3, 9.0] | 3.0 [-1.2, 7.3] | 1.5 [1.4, 1.7] |  | 0.69 [0.51, 0.88] | 0.59 [0.56, 0.62] | 1.00 [1.00, 1.00] |
|  |  | Controls | 12 (1.8%) | 1.8 [1.0, 3.1] | 3.0 [-1.2, 7.3] | 1.4 [1.3, 1.5] |  | 0.61 [0.49, 0.73] | 0.62 [0.62, 0.63] | 1.00 [1.00, 1.00] |

**Supplemental Table 15 footnote:** Table shows the event counts, ten-year cumulative incidence, average risk score and predictive performance statistics. Risk score means are given for each outcome-cancer-risk-score combination. Cumulative incidence and 95% confidence intervals are from Fine-Gray models accounting for the competing risk of death at ten years follow-up. The column marked in yellow indicates calibration differences between the risk score and the observed incidence, where the asterisk (*) indicates where this difference is significantly different from zero. All other asterisks indicate significant differences between cancer cases and controls. The final three columns reflect performance scoring of the candidate risk scores in cancer and control groups. Area under the receiver operating curve (AUC) is calculated from time-dependent analyses adjusted by the competing risk of death. Balanced accuracy and sensitivity are binary metrics calculated using ten-year outcomes, with confidence intervals derived from bootstrapping and permutation testing with 1000 replicates. Calibration is not possible for CHADsVASc as this is an ordinal scale. CVD 1 = combined endpoint including non-fatal myocardial infarction, non-fatal stroke or cardiovascular mortality, where cardiovascular mortality is defined as any death with a primary cause from ICD10 I00-I80. CVD 2 = combined endpoint including everything from CVD 1 plus incident atrial fibrillation, heart failure, non-ischaemic cardiomyopathies and valvular heart disease.

## Supplemental Table 16: Leukemia

| **Outcome** | **Index** | **Group** | **Events within 10 years** | **Cumulative incidence** | **Difference in incidence between cancer and controls** | **Average risk score** | **Difference between observed incidence and risk score** | **AUC** | **Balanced accuracy** | **Sensitivity** |
| --- | --- | --- | --- | --- | --- | --- | --- | --- | --- | --- |
| **CVD 1** | QRISK3 | Cancer | 53 (8.9%) | 8.9* [6.8, 11.3] | 4.2* [1.6, 6.8] | 13.7 [13.0, 14.5] | 4.9* [2.3, 7.4] | 0.65 [0.58, 0.73] | 0.60 [0.52, 0.68] | 0.49 [0.34, 0.64] |
|  |  | Controls | 111 (4.6%) | 4.6* [3.9, 5.5] | 4.2* [1.6, 6.8] | 14.1 [13.7, 14.5] | 9.5* [8.5, 10.5] | 0.70 [0.66, 0.75] | 0.67 [0.65, 0.69] | 0.66 [0.62, 0.70] |
|  | Framingham (BMI) | Cancer | 53 (8.9%) | 8.9* [6.8, 11.3] | 4.2* [1.6, 6.8] | 19.4 [18.4, 20.5] | 10.5* [7.9, 13.2] | 0.63 [0.55, 0.70] | 0.61 [0.54, 0.67] | 0.72 [0.59, 0.84] |
|  |  | Controls | 111 (4.6%) | 4.6* [3.9, 5.5] | 4.2* [1.6, 6.8] | 21.0 [20.4, 21.6] | 16.4* [15.3, 17.5] | 0.66 [0.62, 0.70] | 0.64 [0.63, 0.66] | 0.83 [0.80, 0.86] |
|  | Framingham (blood) | Cancer | 53 (8.9%) | 8.9* [6.8, 11.3] | 4.2* [1.6, 6.8] | 17.6 [16.7, 18.6] | 8.8* [6.1, 11.4] | 0.66 [0.58, 0.74] | 0.62 [0.56, 0.67] | 0.79 [0.69, 0.89] |
|  |  | Controls | 111 (4.6%) | 4.6* [3.9, 5.5] | 4.2* [1.6, 6.8] | 18.7 [18.2, 19.2] | 14.0* [13.0, 15.1] | 0.67 [0.63, 0.72] | 0.66 [0.64, 0.67] | 0.91 [0.88, 0.94] |
|  | SCORE2 / | Cancer | 53 (8.9%) | 8.9* [6.8, 11.3] | 4.2* [1.6, 6.8] | 5.8 [5.5, 6.0] | -3.1* [-5.6, -0.7] | 0.66 [0.59, 0.74] | 0.60 [0.54, 0.65] | 0.79 [0.69, 0.90] |
|  | SCORE-OP | Controls | 111 (4.6%) | 4.6* [3.9, 5.5] | 4.2* [1.6, 6.8] | 6.0 [5.8, 6.1] | 1.3* [0.4, 2.2] | 0.67 [0.62, 0.71] | 0.63 [0.62, 0.65] | 0.88 [0.86, 0.91] |
| **CVD 2** | QRISK3 | Cancer | 134 (22.4%) | 22.4* [19.2, 25.9] | 11.9* [8.2, 15.5] | 13.7 [13.0, 14.5] | -8.7* [-12.2, -5.2] | 0.66 [0.61, 0.71] | 0.61 [0.56, 0.66] | 0.54* [0.45, 0.64] |
|  |  | Controls | 252 (10.6%) | 10.6* [9.4, 11.8] | 11.9* [8.2, 15.5] | 14.1 [13.7, 14.5] | 3.6* [2.2, 4.9] | 0.71 [0.67, 0.74] | 0.67 [0.66, 0.68] | 0.69* [0.67, 0.72] |
|  | Framingham (BMI) | Cancer | 134 (22.4%) | 22.4* [19.2, 25.9] | 11.9* [8.2, 15.5] | 19.4 [18.4, 20.5] | -3.0 [-6.6, 0.6] | 0.65 [0.60, 0.70] | 0.61 [0.56, 0.66] | 0.60* [0.50, 0.69] |
|  |  | Controls | 252 (10.6%) | 10.6* [9.4, 11.8] | 11.9* [8.2, 15.5] | 21.0 [20.4, 21.6] | 10.5* [9.1, 11.9] | 0.69 [0.66, 0.72] | 0.65 [0.63, 0.66] | 0.73* [0.70, 0.75] |
|  | Framingham (blood) | Cancer | 134 (22.4%) | 22.4* [19.2, 25.9] | 11.9* [8.2, 15.5] | 17.6 [16.7, 18.6] | -4.8* [-8.4, -1.2] | 0.64 [0.59, 0.69] | 0.60 [0.55, 0.64] | 0.69* [0.61, 0.78] |
|  |  | Controls | 252 (10.6%) | 10.6* [9.4, 11.8] | 11.9* [8.2, 15.5] | 18.7 [18.2, 19.2] | 8.1* [6.8, 9.5] | 0.68 [0.65, 0.71] | 0.64 [0.63, 0.66] | 0.83* [0.81, 0.85] |
|  | SCORE2 / | Cancer | 134 (22.4%) | 22.4* [19.2, 25.9] | 11.9* [8.2, 15.5] | 5.8 [5.5, 6.0] | -16.7* [-20.1, -13.3] | 0.65 [0.60, 0.71] | 0.61 [0.57, 0.65] | 0.75* [0.67, 0.82] |
|  | SCORE-OP | Controls | 252 (10.6%) | 10.6* [9.4, 11.8] | 11.9* [8.2, 15.5] | 6.0 [5.8, 6.1] | -4.6* [-5.9, -3.3] | 0.67 [0.64, 0.71] | 0.64 [0.63, 0.65] | 0.85* [0.83, 0.86] |
| **Atrial** | CHARGE-AF | Cancer | 60 (10.1%) | 10.1* [7.8, 12.6] | 4.9* [2.1, 7.6] | 2.1 [2.0, 2.3] | -7.9* [-10.5, -5.4] | 0.67 [0.61, 0.74] | 0.61 [0.54, 0.69] | 0.53* [0.39, 0.68] |
| **fibrillation** |  | Controls | 124 (5.2%) | 5.2* [4.4, 6.1] | 4.9* [2.1, 7.6] | 2.2 [2.2, 2.3] | -2.9* [-3.9, -2.0] | 0.73 [0.69, 0.77] | 0.69 [0.67, 0.71] | 0.72* [0.68, 0.75] |
| **Heart** | PCP-HF | Cancer | 32 (5.4%) | 5.4* [3.8, 7.4] | 3.3* [1.2, 5.4] | 3.7 [3.4, 4.0] | -1.7 [-3.7, 0.4] | 0.64 [0.54, 0.74] | 0.62 [0.52, 0.73] | 0.56 [0.35, 0.78] |
| **failure** |  | Controls | 49 (2.1%) | 2.1* [1.5, 2.7] | 3.3* [1.2, 5.4] | 4.1 [3.9, 4.3] | 2.0* [1.4, 2.7] | 0.74 [0.69, 0.80] | 0.70 [0.67, 0.73] | 0.73 [0.68, 0.79] |
| **Stroke** | QStroke | Cancer | 21 (3.5%) | 3.5* [2.2, 5.2] | 1.9* [0.1, 3.7] | 4.8 [4.5, 5.0] | 1.2 [-0.5, 3.0] | 0.53 [0.40, 0.65] | 0.50* [0.36, 0.63] | 0.24* [0.00, 0.52] |
|  |  | Controls | 38 (1.6%) | 1.6* [1.1, 2.2] | 1.9* [0.1, 3.7] | 4.9 [4.8, 5.1] | 3.4* [2.8, 3.9] | 0.70 [0.63, 0.77] | 0.69* [0.65, 0.72] | 0.66* [0.59, 0.72] |
|  | CHADsVASc | Cancer | 21 (3.5%) | 3.5* [2.2, 5.2] | 1.9* [0.1, 3.7] | 1.3 [1.2, 1.4] |  | 0.51 [0.39, 0.64] | 0.50 [0.37, 0.63] | 0.38 [0.13, 0.63] |
|  |  | Controls | 38 (1.6%) | 1.6* [1.1, 2.2] | 1.9* [0.1, 3.7] | 1.3 [1.3, 1.3] |  | 0.62 [0.53, 0.71] | 0.62 [0.59, 0.65] | 0.61 [0.54, 0.67] |

**Supplemental Table 16 footnote:** Table shows the event counts, ten-year cumulative incidence, average risk score and predictive performance statistics. Risk score means are given for each outcome-cancer-risk-score combination. Cumulative incidence and 95% confidence intervals are from Fine-Gray models accounting for the competing risk of death at ten years follow-up. The column marked in yellow indicates calibration differences between the risk score and the observed incidence, where the asterisk (*) indicates where this difference is significantly different from zero. All other asterisks indicate significant differences between cancer cases and controls. The final three columns reflect performance scoring of the candidate risk scores in cancer and control groups. Area under the receiver operating curve (AUC) is calculated from time-dependent analyses adjusted by the competing risk of death. Balanced accuracy and sensitivity are binary metrics calculated using ten-year outcomes, with confidence intervals derived from bootstrapping and permutation testing with 1000 replicates. Calibration is not possible for CHADsVASc as this is an ordinal scale. CVD 1 = combined endpoint including non-fatal myocardial infarction, non-fatal stroke or cardiovascular mortality, where cardiovascular mortality is defined as any death with a primary cause from ICD10 I00-I80. CVD 2 = combined endpoint including everything from CVD 1 plus incident atrial fibrillation, heart failure, non-ischaemic cardiomyopathies and valvular heart disease

## Supplemental Table 17: Polycythemia vera

| **Outcome** | **Index** | **Group** | **Events within 10 years** | **Cumulative incidence** | **Difference in incidence between cancer and controls** | **Average risk score** | **Difference between observed incidence and risk score** | **AUC** | **Balanced accuracy** | **Sensitivity** |
| --- | --- | --- | --- | --- | --- | --- | --- | --- | --- | --- |
| **CVD 1** | QRISK3 | Cancer | 20 (11.0%) | 11.0 [7.0, 16.0] | 5.4 [-0.0, 10.7] | 15.7 [14.4, 17.0] | 4.7 [-0.5, 9.9] | 0.65 [0.55, 0.76] | 0.55 [0.41, 0.69] | 0.35 [0.08, 0.62] |
|  |  | Controls | 41 (5.6%) | 5.6 [4.1, 7.5] | 5.4 [-0.0, 10.7] | 15.3 [14.5, 16.1] | 9.7* [7.7, 11.7] | 0.73 [0.65, 0.81] | 0.69 [0.66, 0.73] | 0.61 [0.55, 0.67] |
|  | Framingham (BMI) | Cancer | 20 (11.0%) | 11.0 [7.0, 16.0] | 5.4 [-0.0, 10.7] | 25.5 [23.4, 27.7] | 14.5* [9.0, 20.0] | 0.64 [0.52, 0.76] | 0.60 [0.46, 0.74] | 0.50 [0.22, 0.78] |
|  |  | Controls | 41 (5.6%) | 5.6 [4.1, 7.5] | 5.4 [-0.0, 10.7] | 23.6 [22.5, 24.7] | 18.0* [15.8, 20.1] | 0.68 [0.59, 0.77] | 0.66 [0.63, 0.69] | 0.56 [0.50, 0.63] |
|  | Framingham (blood) | Cancer | 20 (11.0%) | 11.0 [7.0, 16.0] | 5.4 [-0.0, 10.7] | 20.5 [18.8, 22.3] | 9.6* [4.2, 14.9] | 0.63 [0.52, 0.74] | 0.56 [0.42, 0.70] | 0.35 [0.08, 0.62] |
|  |  | Controls | 41 (5.6%) | 5.6 [4.1, 7.5] | 5.4 [-0.0, 10.7] | 20.7 [19.7, 21.7] | 15.0* [12.9, 17.1] | 0.69 [0.61, 0.78] | 0.68 [0.64, 0.71] | 0.59 [0.52, 0.65] |
|  | SCORE2 / | Cancer | 20 (11.0%) | 11.0 [7.0, 16.0] | 5.4 [-0.0, 10.7] | 6.2 [5.8, 6.7] | -4.7 [-9.8, 0.3] | 0.66 [0.54, 0.77] | 0.55 [0.42, 0.68] | 0.25 [0.00, 0.50] |
|  | SCORE-OP | Controls | 41 (5.6%) | 5.6 [4.1, 7.5] | 5.4 [-0.0, 10.7] | 6.4 [6.1, 6.7] | 0.8 [-1.1, 2.6] | 0.70 [0.61, 0.79] | 0.67 [0.64, 0.70] | 0.54 [0.47, 0.60] |
| **CVD 2** | QRISK3 | Cancer | 43 (23.6%) | 23.6* [17.7, 30.0] | 12.6* [5.8, 19.5] | 15.7 [14.4, 17.0] | -8.0* [-14.5, -1.4] | 0.67 [0.58, 0.76] | 0.62 [0.55, 0.70] | 0.77 [0.64, 0.90] |
|  |  | Controls | 80 (11.0%) | 11.0* [8.8, 13.4] | 12.6* [5.8, 19.5] | 15.3 [14.5, 16.1] | 4.3* [1.8, 6.8] | 0.73 [0.67, 0.78] | 0.68 [0.66, 0.69] | 0.85 [0.82, 0.88] |
|  | Framingham (BMI) | Cancer | 43 (23.6%) | 23.6* [17.7, 30.0] | 12.6* [5.8, 19.5] | 25.5 [23.4, 27.7] | 1.9 [-4.9, 8.6] | 0.67 [0.58, 0.76] | 0.66 [0.57, 0.76] | 0.58 [0.41, 0.76] |
|  |  | Controls | 80 (11.0%) | 11.0* [8.8, 13.4] | 12.6* [5.8, 19.5] | 23.6 [22.5, 24.7] | 12.6* [10.0, 15.3] | 0.71 [0.65, 0.77] | 0.67 [0.65, 0.70] | 0.57 [0.53, 0.62] |
|  | Framingham (blood) | Cancer | 43 (23.6%) | 23.6* [17.7, 30.0] | 12.6* [5.8, 19.5] | 20.5 [18.8, 22.3] | -3.1 [-9.7, 3.5] | 0.66 [0.57, 0.75] | 0.64 [0.56, 0.73] | 0.70 [0.54, 0.86] |
|  |  | Controls | 80 (11.0%) | 11.0* [8.8, 13.4] | 12.6* [5.8, 19.5] | 20.7 [19.7, 21.7] | 9.7* [7.1, 12.3] | 0.70 [0.65, 0.76] | 0.66 [0.64, 0.68] | 0.74 [0.70, 0.78] |
|  | SCORE2 / | Cancer | 43 (23.6%) | 23.6* [17.7, 30.0] | 12.6* [5.8, 19.5] | 6.2 [5.8, 6.7] | -17.4* [-23.8, -11.0] | 0.67 [0.58, 0.77] | 0.63 [0.54, 0.71] | 0.65 [0.49, 0.81] |
|  | SCORE-OP | Controls | 80 (11.0%) | 11.0* [8.8, 13.4] | 12.6* [5.8, 19.5] | 6.4 [6.1, 6.7] | -4.6* [-7.0, -2.2] | 0.71 [0.65, 0.77] | 0.67 [0.65, 0.69] | 0.72 [0.69, 0.76] |
| **Atrial** | CHARGE-AF | Cancer | 18 (9.9%) | 9.9 [6.1, 14.8] | 4.9 [-0.2, 10.1] | 2.5 [2.2, 2.8] | -7.4* [-12.3, -2.5] | 0.72 [0.59, 0.85] | 0.68 [0.56, 0.79] | 0.72 [0.50, 0.94] |
| **fibrillation** |  | Controls | 36 (4.9%) | 4.9 [3.5, 6.7] | 4.9 [-0.2, 10.1] | 2.4 [2.2, 2.5] | -2.6* [-4.3, -0.8] | 0.80 [0.73, 0.86] | 0.76 [0.73, 0.79] | 0.86 [0.81, 0.91] |
| **Heart** | PCP-HF | Cancer | 16 (8.8%) | 8.8* [5.2, 13.5] | 6.6* [1.7, 11.4] | 5.2 [4.3, 6.0] | -3.6 [-8.4, 1.2] | 0.65 [0.49, 0.81] | 0.64 [0.46, 0.82] | 0.44 [0.08, 0.79] |
| **failure** |  | Controls | 16 (2.2%) | 2.2* [1.3, 3.5] | 6.6* [1.7, 11.4] | 4.5 [4.1, 4.8] | 2.3* [0.9, 3.6] | 0.66 [0.53, 0.79] | 0.67 [0.62, 0.71] | 0.50 [0.41, 0.59] |
| **Stroke** | QStroke | Cancer | 8 (4.4%) | 4.4 [2.1, 8.1] | 2.6 [-1.3, 6.5] | 5.5 [5.0, 6.0] | 1.1 [-2.6, 4.8] | 0.63 [0.44, 0.82] | 0.58 [0.38, 0.78] | 0.38 [0.00, 0.78] |
|  |  | Controls | 13 (1.8%) | 1.8 [1.0, 3.0] | 2.6 [-1.3, 6.5] | 5.2 [4.9, 5.4] | 3.4* [2.2, 4.6] | 0.75 [0.65, 0.86] | 0.75 [0.69, 0.80] | 0.69 [0.58, 0.80] |
|  | CHADsVASc | Cancer | 8 (4.4%) | 4.4 [2.1, 8.1] | 2.6 [-1.3, 6.5] | 1.5 [1.3, 1.6] |  | 0.66 [0.47, 0.86] | 0.65 [0.45, 0.86] | 0.75 [0.34, 1.00] |
|  |  | Controls | 13 (1.8%) | 1.8 [1.0, 3.0] | 2.6 [-1.3, 6.5] | 1.1 [1.1, 1.2] |  | 0.64 [0.49, 0.78] | 0.62 [0.56, 0.67] | 0.54 [0.43, 0.65] |

**Supplemental Table 17 footnote:** Table shows the event counts, ten-year cumulative incidence, average risk score and predictive performance statistics. Risk score means are given for each outcome-cancer-risk-score combination. Cumulative incidence and 95% confidence intervals are from Fine-Gray models accounting for the competing risk of death at ten years follow-up. The column marked in yellow indicates calibration differences between the risk score and the observed incidence, where the asterisk (*) indicates where this difference is significantly different from zero. All other asterisks indicate significant differences between cancer cases and controls. The final three columns reflect performance scoring of the candidate risk scores in cancer and control groups. Area under the receiver operating curve (AUC) is calculated from time-dependent analyses adjusted by the competing risk of death. Balanced accuracy and sensitivity are binary metrics calculated using ten-year outcomes, with confidence intervals derived from bootstrapping and permutation testing with 1000 replicates. Calibration is not possible for CHADsVASc as this is an ordinal scale. CVD 1 = combined endpoint including non-fatal myocardial infarction, non-fatal stroke or cardiovascular mortality, where cardiovascular mortality is defined as any death with a primary cause from ICD10 I00-I80. CVD 2 = combined endpoint including everything from CVD 1 plus incident atrial fibrillation, heart failure, non-ischaemic cardiomyopathies and valvular heart disease

## Supplemental Table 18: Myelodysplastic syndrome

| **Outcome** | **Index** | **Group** | **Events within 10 years** | **Cumulative incidence** | **Difference in incidence between cancer and controls** | **Average risk score** | **Difference between observed incidence and risk score** | **AUC** | **Balanced accuracy** | **Sensitivity** |
| --- | --- | --- | --- | --- | --- | --- | --- | --- | --- | --- |
| **CVD 1** | QRISK3 | Cancer | 11 (14.9%) | 14.9* [7.9, 24.0] | 12.2* [2.8, 21.6] | 14.3 [12.3, 16.2] | -0.6 [-9.9, 8.7] | 0.67 [0.51, 0.83] | 0.56 [0.36, 0.77] | 0.36 [0.00, 0.77] |
|  |  | Controls | 8 (2.7%) | 2.7* [1.3, 5.0] | 12.2* [2.8, 21.6] | 14.7 [13.6, 15.8] | 12.0* [9.4, 14.6] | 0.79 [0.67, 0.92] | 0.76 [0.69, 0.82] | 0.75 [0.63, 0.87] |
|  | Framingham (BMI) | Cancer | 11 (14.9%) | 14.9* [7.9, 24.0] | 12.2* [2.8, 21.6] | 20.7 [17.3, 24.1] | 5.8 [-3.9, 15.5] | 0.68 [0.52, 0.85] | 0.60 [0.37, 0.83] | 0.36 [0.00, 0.81] |
|  |  | Controls | 8 (2.7%) | 2.7* [1.3, 5.0] | 12.2* [2.8, 21.6] | 21.5 [19.9, 23.0] | 18.7* [15.9, 21.6] | 0.79 [0.66, 0.92] | 0.77 [0.71, 0.84] | 0.75 [0.62, 0.88] |
|  | Framingham (blood) | Cancer | 11 (14.9%) | 14.9* [7.9, 24.0] | 12.2* [2.8, 21.6] | 17.9 [15.1, 20.6] | 3.0 [-6.5, 12.5] | 0.68 [0.52, 0.85] | 0.57 [0.35, 0.78] | 0.55 [0.12, 0.97] |
|  |  | Controls | 8 (2.7%) | 2.7* [1.3, 5.0] | 12.2* [2.8, 21.6] | 18.8 [17.5, 20.2] | 16.1* [13.4, 18.8] | 0.77 [0.62, 0.91] | 0.72 [0.66, 0.78] | 0.88 [0.76, 0.99] |
|  | SCORE2 / | Cancer | 11 (14.9%) | 14.9* [7.9, 24.0] | 12.2* [2.8, 21.6] | 6.0 [5.3, 6.7] | -8.9 [-18.0, 0.2] | 0.74 [0.59, 0.89] | 0.60 [0.41, 0.79] | 0.27 [0.00, 0.65] |
|  | SCORE-OP | Controls | 8 (2.7%) | 2.7* [1.3, 5.0] | 12.2* [2.8, 21.6] | 6.1 [5.7, 6.5] | 3.4* [1.0, 5.8] | 0.78 [0.62, 0.93] | 0.74 [0.69, 0.80] | 0.62 [0.51, 0.74] |
| **CVD 2** | QRISK3 | Cancer | 18 (24.3%) | 24.3* [15.2, 34.6] | 13.2* [2.2, 24.2] | 14.3 [12.3, 16.2] | -10.1 [-20.5, 0.4] | 0.70 [0.58, 0.83] | 0.58* [0.43, 0.72] | 0.39* [0.12, 0.66] |
|  |  | Controls | 33 (11.1%) | 11.1* [7.9, 15.0] | 13.2* [2.2, 24.2] | 14.7 [13.6, 15.8] | 3.6 [-0.5, 7.6] | 0.78 [0.71, 0.85] | 0.75* [0.71, 0.79] | 0.76* [0.69, 0.83] |
|  | Framingham (BMI) | Cancer | 18 (24.3%) | 24.3* [15.2, 34.6] | 13.2* [2.2, 24.2] | 20.7 [17.3, 24.1] | -3.6 [-14.4, 7.2] | 0.68 [0.54, 0.83] | 0.63 [0.48, 0.79] | 0.50 [0.21, 0.79] |
|  |  | Controls | 33 (11.1%) | 11.1* [7.9, 15.0] | 13.2* [2.2, 24.2] | 21.5 [19.9, 23.0] | 10.3* [6.1, 14.5] | 0.79 [0.72, 0.86] | 0.73 [0.69, 0.76] | 0.70 [0.63, 0.77] |
|  | Framingham (blood) | Cancer | 18 (24.3%) | 24.3* [15.2, 34.6] | 13.2* [2.2, 24.2] | 17.9 [15.1, 20.6] | -6.5 [-17.1, 4.2] | 0.67 [0.53, 0.81] | 0.62 [0.48, 0.77] | 0.50 [0.22, 0.78] |
|  |  | Controls | 33 (11.1%) | 11.1* [7.9, 15.0] | 13.2* [2.2, 24.2] | 18.8 [17.5, 20.2] | 7.7* [3.5, 11.8] | 0.78 [0.71, 0.86] | 0.74 [0.71, 0.78] | 0.76 [0.69, 0.82] |
|  | SCORE2 / | Cancer | 18 (24.3%) | 24.3* [15.2, 34.6] | 13.2* [2.2, 24.2] | 6.0 [5.3, 6.7] | -18.4* [-28.7, -8.0] | 0.71 [0.57, 0.84] | 0.66 [0.55, 0.78] | 0.72 [0.52, 0.93] |
|  | SCORE-OP | Controls | 33 (11.1%) | 11.1* [7.9, 15.0] | 13.2* [2.2, 24.2] | 6.1 [5.7, 6.5] | -5.1* [-9.0, -1.1] | 0.78 [0.71, 0.85] | 0.76 [0.73, 0.79] | 0.91 [0.86, 0.96] |
| **Atrial** | CHARGE-AF | Cancer | 8 (10.8%) | 10.8 [5.0, 19.1] | 4.1 [-4.9, 13.0] | 2.4 [1.9, 2.9] | -8.4* [-16.7, -0.1] | 0.68 [0.56, 0.81] | 0.55* [0.36, 0.74] | 0.38* [0.01, 0.74] |
| **fibrillation** |  | Controls | 20 (6.8%) | 6.8 [4.3, 10.0] | 4.1 [-4.9, 13.0] | 2.4 [2.2, 2.6] | -4.4* [-7.6, -1.1] | 0.83 [0.74, 0.92] | 0.78* [0.73, 0.82] | 0.85* [0.76, 0.94] |
| **Heart** | PCP-HF | Cancer | 7 (9.5%) | 9.5 [4.1, 17.5] | 6.4 [-2.0, 14.8] | 4.5 [3.1, 6.0] | -4.9 [-13.1, 3.2] | 0.67 [0.48, 0.87] | 0.60 [0.39, 0.81] | 0.57 [0.15, 0.99] |
| **failure** |  | Controls | 9 (3.0%) | 3.0 [1.5, 5.5] | 6.4 [-2.0, 14.8] | 4.2 [3.8, 4.7] | 1.2 [-1.3, 3.7] | 0.79 [0.67, 0.90] | 0.75 [0.69, 0.81] | 0.89 [0.77, 1.00] |
| **Stroke** | QStroke | Cancer | 7 (9.5%) | 9.5* [4.1, 17.5] | 8.4* [0.2, 16.6] | 5.2 [4.5, 6.0] | -4.2 [-12.3, 3.8] | 0.63 [0.42, 0.84] | 0.60 [0.38, 0.81] | 0.71 [0.29, 1.00] |
|  |  | Controls | 3 (1.0%) | 1.0* [0.3, 2.8] | 8.4* [0.2, 16.6] | 5.2 [4.9, 5.6] | 4.2* [2.4, 6.0] | 0.73 [0.53, 0.94] | 0.74 [0.67, 0.82] | 1.00 [0.85, 1.00] |
|  | CHADSVASc | Cancer | 7 (9.5%) | 9.5* [4.1, 17.5] | 8.4* [0.2, 16.6] | 1.4 [1.2, 1.7] |  | 0.62 [0.37, 0.87] | 0.44 [0.23, 0.65] | 0.29 [0.00, 0.69] |
|  |  | Controls | 3 (1.0%) | 1.0* [0.3, 2.8] | 8.4* [0.2, 16.6] | 1.4 [1.3, 1.5] |  | 0.63 [0.45, 0.80] | 0.64 [0.55, 0.73] | 0.67 [0.49, 0.84] |

**Supplemental Table 18 footnote:** Table shows the event counts, ten-year cumulative incidence, average risk score and predictive performance statistics. Risk score means are given for each outcome-cancer-risk-score combination. Cumulative incidence and 95% confidence intervals are from Fine-Gray models accounting for the competing risk of death at ten years follow-up. The column marked in yellow indicates calibration differences between the risk score and the observed incidence, where the asterisk (*) indicates where this difference is significantly different from zero. All other asterisks indicate significant differences between cancer cases and controls. The final three columns reflect performance scoring of the candidate risk scores in cancer and control groups. Area under the receiver operating curve (AUC) is calculated from time-dependent analyses adjusted by the competing risk of death. Balanced accuracy and sensitivity are binary metrics calculated using ten-year outcomes, with confidence intervals derived from bootstrapping and permutation testing with 1000 replicates. Calibration is not possible for CHADsVASc as this is an ordinal scale. CVD 1 = combined endpoint including non-fatal myocardial infarction, non-fatal stroke or cardiovascular mortality, where cardiovascular mortality is defined as any death with a primary cause from ICD10 I00-I80. CVD 2 = combined endpoint including everything from CVD 1 plus incident atrial fibrillation, heart failure, non-ischaemic cardiomyopathies and valvular heart disease

## Supplemental Figure 6: Cumulative death risk curves by Cancer group


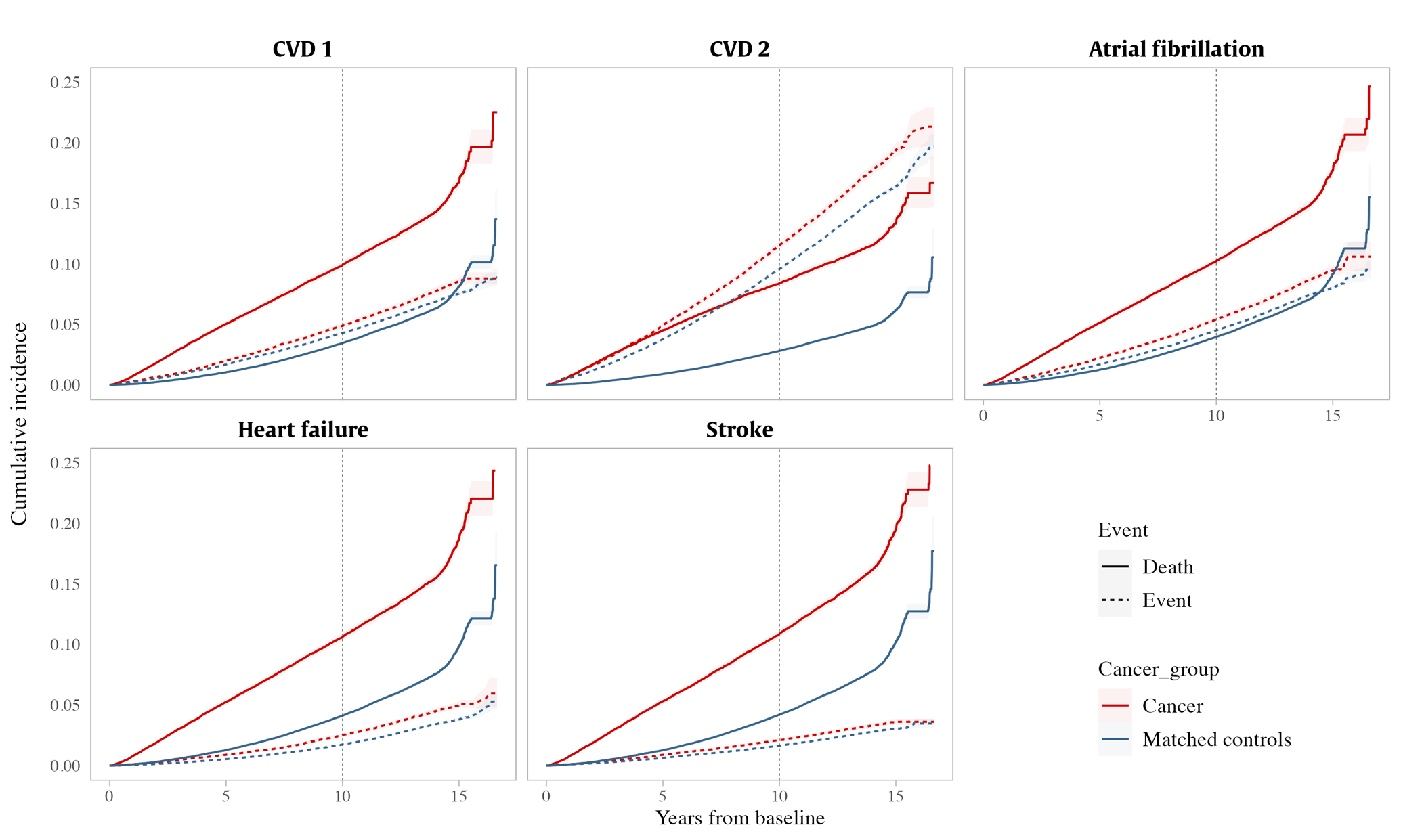


**Supplemental Figure 6 footnote:** Cumulative incidence functions and 95% confidence intervals across follow-up for Fine-Gray models showing competing risk of death (solid lines) along with event incidence rates (dotted lines). Cancer cases are shown in red, with Controls in dark blue. Where sample sizes are very large the shaded 95% confidence intervals may be too narrow to visualise clearly. The vertical dotted line indicates ten-year follow-up. Competing risk of death is slightly different between outcomes depending on whether death coincided with the outcome of interest, in which case the death would count as an outcome event rather than as a competing death to be adjusted for.
